# Plant-DTI: Extending the landscape of TF protein and DNA interaction in plants by a machine learning-based approach

**DOI:** 10.3389/fpls.2022.970018

**Published:** 2022-08-23

**Authors:** Bhukrit Ruengsrichaiya, Chakarida Nukoolkit, Saowalak Kalapanulak, Treenut Saithong

**Affiliations:** ^1^Bioinformatics and Systems Biology Program, School of Bioresources and Technology and School of Information Technology, King Mongkut’s University of Technology Thonburi (Bang KhunThian), Bangkok, Thailand; ^2^School of Information Technology, King Mongkut’s University of Technology Thonburi, Bangkok, Thailand; ^3^Center for Agricultural Systems Biology, Systems Biology and Bioinformatics Research Group, Pilot Plant Development and Training Institute, King Mongkut’s University of Technology Thonburi (Bang KhunThian), Bangkok, Thailand

**Keywords:** TF-TFBS interactions, transcription factor, transcription factor binding site, DNA binding domain, transcriptional regulation, machine-learning, plants

## Abstract

As a sessile organism, plants hold elaborate transcriptional regulatory systems that allow them to adapt to variable surrounding environments. Current understanding of plant regulatory mechanisms is greatly constrained by limited knowledge of transcription factor (TF)–DNA interactions. To mitigate this problem, a Plant-DTI predictor (**Plant D**BD-**T**FBS **I**nteraction) was developed here as the first machine-learning model that covered the largest experimental datasets of 30 plant TF families, including 7 plant-specific DNA binding domain (DBD) types, and their transcription factor binding sites (TFBSs). Plant-DTI introduced a novel TFBS feature construction, called TFBS base-preference, which enhanced the specificity of TFBS to DBD types. The proposed model showed better predictive performance with the TFBS base-preference than the simple binary representation. Plant-DTI was validated with 22 independent ChIP-seq datasets. It accurately predicted the measured DBD-TFBS pairs along with their TFBS motifs, and effectively predicted interactions of other TFs containing similar DBD types. Comparing to the existing state-of-art methods, Plant-DTI prediction showed a figure of merit in sensitivity and specificity with respect to the position weight matrix (PWM) and TSPTFBS methods. Finally, the proposed Plant-DTI model helped to fill the knowledge gap in the regulatory mechanisms of the cassava sucrose synthase 1 gene (MeSUS1). Plant-DTI predicted MeERF72 as a regulator of MeSUS1 in consistence with the yeast one-hybrid (Y1H) experiment. Taken together, Plant-DTI would help facilitate the prediction of TF-TFBS and TF-target gene (TG) interactions, thereby accelerating the study of transcriptional regulatory systems in plant species.

## Introduction

Transcriptional regulation controls the action and activity of genes by regulating the time and level of gene expression. A punctual expression pattern of genes is a regulatory code synchronizing individual biological processes under a particular condition, then modulating phenotypes as well as physiological responses of an organism (Li et al., 2015; Kumar et al., 2017). Extensive studies have been conducted to decipher the relationships behind the transcriptional regulatory process, and at most are based upon analysis of co-expression patterns (Chen et al., 2012; Yu et al., 2015) and DNA footprints (Neph et al., 2012; Franco-Zorrilla et al., 2014; Sullivan et al., 2014). The former approach globally analyzes the association of regulatory genes from their individual expression profiles measured by condition-specific transcriptome, whilst the latter infers the physical interaction of transcription factors (TFs) and regulated target genes (TGs) through the conserved binding sites. Advanced molecular techniques demonstrate that TFs through the DNA binding domains (DBDs) specifically bind onto the upstream sequence (promoter) of the regulated genes at transcription factor binding sites (TFBSs), which are 5–15 bp conserved regions in complement with a particular TF. The interactions of TFs *via* DBD and TFBSs have been investigated by both *in vitro* methods, such as electrophoretic mobility shift assay (EMSA), systematic evolution of ligands by exponential enrichment (SELEX), protein binding microarray (PBM; Berger and Bulyk, 2006), and *in vivo* methods, such as yeast-one-hybrid (Y1H; Ouwerkerk and Meijer, 2001; Ferraz et al., 2021), and ChIP-seq (Park, 2009). The plethora of data on TF-TFBS interactions generated and deposited in public databases, for example TRANSFAC (Wingender et al., 1996), CIS-BP (Weirauch et al., 2014), and Plant ChIP-seq Database (PCBase; Chow et al., 2019), has improved our knowledge of transcriptional regulation in a broad range of eukaryotes. However, they are mostly limited to a small group of well-studied organisms such as yeast (Monteiro et al., 2020), humans and mice (Kulakovskiy et al., 2018), and Arabidopsis (Yilmaz et al., 2011).

Computational approaches are widely introduced to exploit the wealth of known TF-TFBS interactions, from model organisms to the less studied ones. These methods are developed from diverse concepts, yet all rely on a universal hypothesis of evolutionary conservation. There are two main classes of prediction methods: pattern-matching based, and machine-learning based. Pattern matching-based methods predict TF-TFBS interactions based on sequence similarity, whereby the interaction of the TF and TFBS is predicted if the query DNA sequence and the known TFBS of the TF are alike. The position weight matrix (PWM) of interested TF are explored in promoter sequences to infer specific binding and regulation of target genes (Kel et al., 2003; Marinescu et al., 2005; Jayaram et al., 2016). Previous studies showed that this kind of pattern-matching based approach frequently employed and well applicable to explore TF-TFBS interactions in a broad range of organisms (Kel et al., 2003; Marinescu et al., 2005; Turatsinze et al., 2008; Chow et al., 2016; Jin et al., 2017); however, the predictions are always constrained by the number of data in the collection. Machine-learning based methods, on the other hand, make a prediction according to the built-on knowledge of the existing data. The prediction criteria are formulated from learning information of the available interactions of TF-TFBS, also DBD-TFBS, using various approaches. Qian et al. (2006) modeled the TF recognition sequences from binary-based patterns of short TFBS motifs and the TFs with corresponding gene ontology using the k-nearest neighbor classifier, with approximately 77% model accuracy. Lee et al. (2017) later introduced an SVM model with more features of TFs and TFBS properties, improving the accuracy of prediction to 82%. The model considered the global composition of residues in TFs and TFBSs sequences using the composition, transition, and distribution of residues in the sequences. A more recent study achieved 99% model prediction accuracy by incorporating the physicochemical properties of TF proteins, structural conformation and bonding potential of TFs and DNA (Khamis et al., 2018). Due to data availability, machine-learning based prediction is currently applied to human data at most.

Unlike a typical eukaryote, plant species contain exclusive TF families for their special organ development and the peculiar response to environmental perturbation (Yamasaki et al., 2013; Lehti-Shiu et al., 2017). The TF-TFBS interactions of those are relatively little explored, and most of which are identified from model species like Arabidopsis and based on pattern-matching based approaches. AthaMap is a major source of TF-TFBS interactions in Arabidopsis, consisting of 2,458,243 interacting pairs from 23 TFs in 13 TF families predicted by pattern matching (Steffens et al., 2004). PlantTFDB later extended the inference of TF-TG interaction to 132 plants species, including 50,850,582 interactions of 338 TFs in 45 TF families (Jin et al., 2017). The FunTFBS algorithm developed for PlantRegMap to refine the TFBS prediction identified 2,493,577 highly probable putative TF-TG interactions in 63 plants species (Tian et al., 2020). Machine-learning based prediction has been explored particularly for plant species since 2007 (Dai et al., 2007; Cui et al., 2014). Specifically, the SVM model was constructed to explore the potential TFBSs of auxin response factor (ARF) TFs in Arabidopsis using gene co-expression data and information on conserved binding site sequences. The model proved effective despite being based only on a single family of ARF TFs. The TSPTFBS was developed last year as a TFBS prediction tool particularly for plant species (Liu et al., 2021). This tool used deep learning to model 265 Arabidopsis TFs with their 201-bp nucleotide binding sequences. The model was outperformed, but could not predict candidate TFBSs with their typically size of 5–15 bp (Yu et al., 2016). While large numbers of interactions have been predicted during the past decade, many more are expected to remain unknown considering the variety of TFs and TF families identified in plant species and the complexity of transcriptional regulation in plants (Riechmann et al., 2000; Shiu et al., 2005; Lehti-Shiu et al., 2017). The general lack of information, especially on plant-specific TF-TFBS interactions when compared to other eukaryotes, represents a challenge in the attempt to unravel the complex transcriptional regulation in plant species.

Here, a machine-learning based predictor, namely Plant-DTI (**Plant D**NA binding domain (DBD) – **T**ranscription factor binding site (TFBS) **I**nteraction) was developed to extend the resource of TF-TFBS interactions in plant species. Plant-DTI provided a rigorous prediction according to three key strengths of the model: (i) containing large coverage of experimental DBD-TFBS interaction information in plant species, (ii) employing the novel proposed feature construction of TFBS, which enhanced the specificity of TFBS to DBD types, and (iii) proving high predictive performances over existing state-of-art methods. Particularly, Plant-DTI model was constructed from the DBD-TFBS information of at least 22 plant species in the literature, enabling the prediction to cover 30 of the 63 TF families in plants and 336 TFBS motifs (Jin et al., 2014; Weirauch et al., 2014). As a predictor for plant species, Plant-DTI has the advantage of including a large amount of training data compared to other existing plant models. In addition, the implemented TFBS base-preference feature is an improvement on the traditional binary sequence. The predictive performance of Plant-DTI was rewarding, with high accuracy, sensitivity, precision, and F1-score. The proposed model was finally validated and compared to other state-of-art methods using independent ChIP-seq, DAP-seq and negative data to consolidate its prediction power. At last, Plant-DTI was applied to explore the putative TF regulators of sucrose synthase 1 (MeSUS1) gene in cassava. MeSUS1 is a crucial sucrolytic enzyme that is believed to determine crop yield; however, its regulation, especially at the transcriptional level, is little understood compared to other key enzymes. Application of Plant-DTI revealed 150 interactions (136 novel interactions) of TFs linked to the MeSUS1 gene, which connected the transcriptional regulation of this gene to the exposed environments. MeSUS1gene expression was predicted to be regulated by various stress-responsive TFs, especially TFs in the ERF family which possibly key regulating TFs of MeSUS1 in response to stress conditions. Plant-DTI, in summary, would help narrow down the knowledge gap on TF-TFBS interaction and improve our understanding of transcriptional regulation in plant species, especially in less-studied but strategic staple crops (Lai et al., 2019). The Plant-DTI^1^ is implemented as a web application tool and freely.

## Materials and methods

### Plant DBD-TFBS interactions datasets

The DBD-TFBS interaction were collected from CIS-BP database version 1.02 (Weirauch et al., 2014). It was first retrieved 786 interactions of 611 TFs and 719 TFBSs based on experimental measurement in plant species. The numbers were reduced to 424 interactions of 388 TFs and 424 TFBSs, after removing the redundant interactions. Considering only monotypic-DBD TFs (TFs containing only one DBD type) and TFBS with 7–15 bp in length, it finally obtained 343 DBD-TFBS interactions (325 TFs, 343 TFBSs) from 22 plant species. Since CIS-BP did not provide exact TFBS sequences, here, all possible TFBS motif patterns were determined from IUPAC TFBS sequences. In total, 3,287,619 non-redundant interactions were conjectured for 325 TFs and 343 TFBSs (Supplementary File 2).

### Modeling DBD-TFBS interactions

#### TFBS feature representation

In this work, TFBS sequences were represented by two different bases: (i) traditional binary representation and (ii) the newly proposed ‘TFBS base-preference’ representation.

##### Binary representation

The TFBS sequences were simply denoted by a series of binary numbers given specifically to each nucleotide base variety, A – 1000, T – 0100, C – 0010, and G – 0001. For example, the ‘CAGCCG’ sequence was represented by ‘001010000001001000100001’. A feature vector of 343 TFBS motif sequences was constructed according to their lengths (*L* ∈ {7, 8, 9, …, 15}), then yielded vectors in range of 28 to 60 features (4 × *L*).

##### TFBS base-preference

The ‘TFBS base-preference’ was developed, herein, as an information-contentive representation. Each TFBS motif was represented by the probability of having the nucleotide base (A, T, C, and G) at a particular position in the TFBS sequence. The probability $P_{j}\left(X\right)$ was calculated for each particular DBD type and TFBS length from the existing data of the 343 DBD-TFBS interactions in plants (Supplementary Table S3). The number of TFBS features for a DBD type ranged from 28 to 60 (4*L) depending on their TFBS length. Supplementary Figure S1 showed an example of TFBS feature construction and representation of TFBS base-preference. The probability of the nucleotide base *X* ∈ {*A*, *T*, *C*, *G*} at *j*th position was determined by the following equation, and the resulting $P_{j}\left(X\right)$ was demonstrated in box plots (Supplementary Table S3).


(1) $$P_{j}\left(X\right)=\frac{\sum_{i=1}^{N}P_{j}{\left(X\right)}_{i}}{N},$$

where *i* is the number of DBD-TFBS interactions (= {1, 2, …, *N*}), and *N* is the total number of DBD-TFBS interactions for a DBD type.

#### DBD feature representation

Each DBD sequence was represented by the amino acid binding mode preference to DNA from Luscombe (2001) and Khamis et al. (2018). The amino acid sequences of DBD were denoted by three features according to (1) *hb* – hydrogen bonding interaction, (2) *dw* – van Der Waal binding interaction, and (3) *wb* – weakly binding interaction. The binding mode preference of feature *k* ∈ {*hb*, *dw*, *wb*} was determined as below.


(2) $$Feature_{k}=\frac{n_{k}}{l}\;,$$

where *n* is the number of amino acids in feature class *k* contained in DBD length *l*. The amino acids in each class were as follows; *hb* – consisting of Arg, Lys, His, Ser, Asn, Gln, Asp., and Glu, *dw* consisting of Phe, Pro, Thr, Gly, Ala, Val, Leu, Iso, and Tyr, and *wb* -- consisting of Cys, Met, and Trp.

#### Combined DBD and TFBS features information

**Figure 1:**
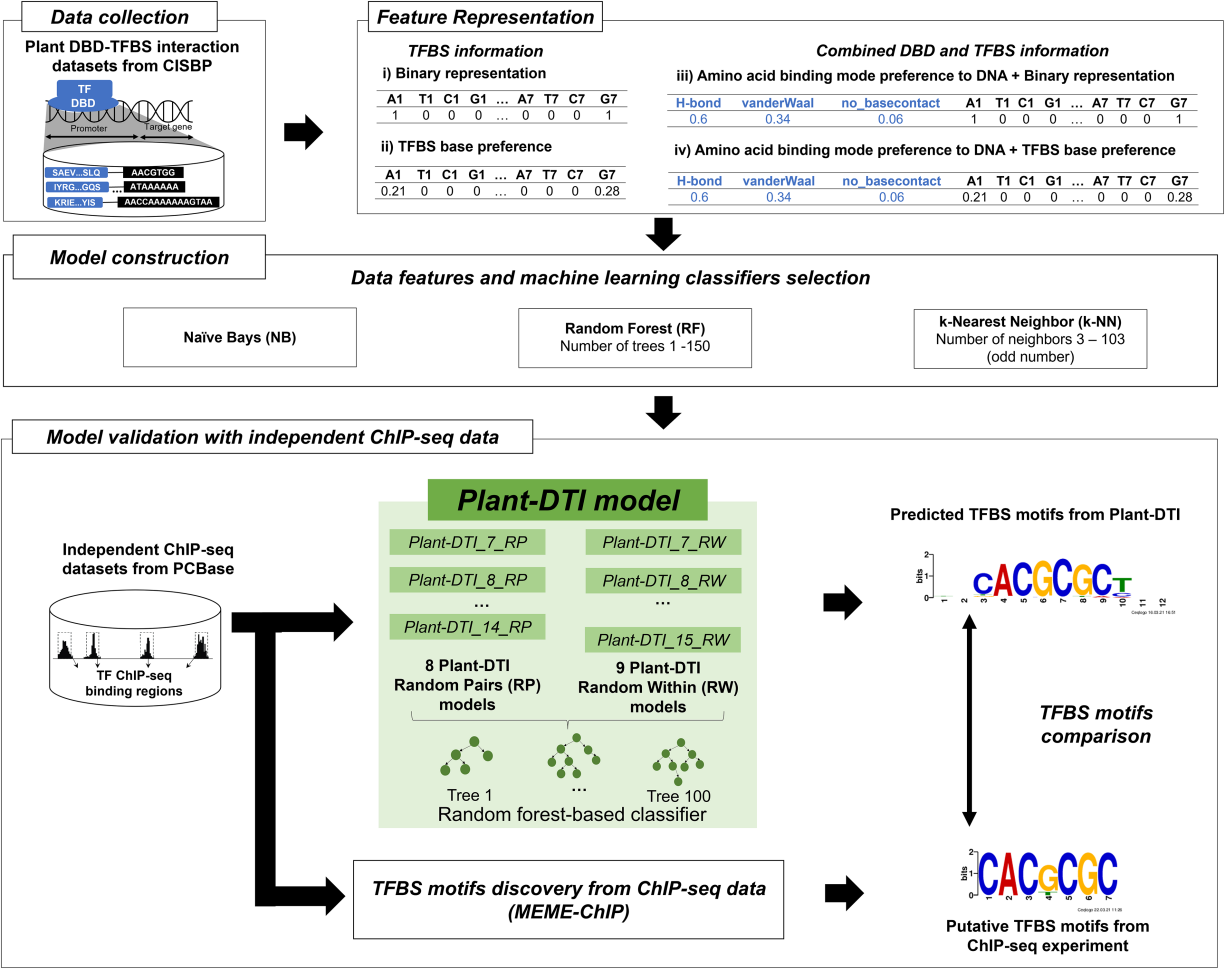
Schematic overview of Plant-DTI model framework.

The individual DBD and TFBS features were combined into a DBD-TFBS interaction feature vector as shown in Figure 1 (feature representation box). There were two types of interaction feature vectors, (i) a combined feature of amino acid binding mode preference of DBD and binary representation of TFBS, and (ii) a combined feature of amino acid binding mode preference of DBD and TFBS base preference. The number of features for each DBD-TFBS interaction was a summation of those for a paired DBD and TFBS.

### Positive and negative data

#### Positive data

The existing experimental data of DBD-TFBS interaction in CIS-BP database was exploited here as a positive dataset. It included 3,287,619 non-redundant DBD-TFBS interactions of 325 TFs. The positive data were separated into nine groups based on the TFBS length (7–15 bp).

#### Negative data

The negative data of DBD-TFBS interaction was generated based on two conceptual bases, (i) random-pair (RP) – random permutation of the interacting DBD and TFBS pairs, and (ii) random-within (RW) – random permutation of nucleotide bases within the TFBS sequence. All generated negative data of DBD-TFBS interactions must exclude any known DBD-TFBS interactions (positive data).

##### Random DBD-TFBS pairs (RP)

For RP basis, TF proteins were presumed to recognize their TFBSs as the interacting pairs. The negative data set was constructed by shuffling pairs of known DBD-TFBS interactions in CIS-BP database. The generated negative data was assured no positive data contained, while maintained similar data proportion to the positive dataset. In this process, 13 TFBSs were excluded because their generated negative datasets were not satisfied the setting criteria. The RP model, finally, included 330 TFBSs and 313 TFs (Supplementary Table S1).

##### Random nucleotides within TFBS sequences (RW)

For RW, TF proteins were presumed to interact with TFBS by a recognitive pattern of TFBS sequence. The negative data of DBD-TFBS interactions were constructed by a random permutation of nucleotide bases within TFBS motif sequence. All generated negative data were lastly checked against the known DBD-TFBS interactions in CIS-BP database to ensure no positive data included and have the same proportion as positive data. There were 15 TFBSs removed from the model according to their weak negative datasets. In total, the RW model included 328 TFBSs and 310 TFs (Supplementary Table S1).

#### Training and test datasets

In overall, the training and test datasets for Plant-DTI model construction were consisted of 336 TFBSs and 318 TFs from 22 plant species. The total modelled data were 3,931,070 and 2,482,629 interactions for RP and RW models, respectively. The model was constructed for each particular TFBS length, where the training to test data proportion was set to 70:30 of all data entity (see more details in Supplementary Tables S1, S2).

### Plant-DTI model construction

To develop Plant-DTI, various combination of model components from different TFBS feature representations (binary and TFBS base-preference for TFBS feature), inclusion of DBD features to TFBS features (combined amino acid binding mode preference for DBD and TFBS features), and a variety of machine learning classifiers [Naïve Bayes (NB), Random Forest (RF), and k-Nearest Neighbor (k-NN)] were primarily tested for the optimal construction. All preliminary models were evaluated using F1-score (Figure 1). The classifier and feature dataset that offered the best performance based on the F1-score were selected to construct the Plant-DTI model.

The NB model was the simplest probabilistic classifier selected as to accommodate a massive modelled data (Zhang, 2004; Han et al., 2012). RF model is an ensemble learning classifier, whose performance is basically dependent on number of decision trees. The model was first constructed based on a default setting of scikit-learn (Pedregosa et al., 2011). Hyperparameter optimization of RF was later performed through 10-fold cross-validation, where the tree numbers were parameterized from 1 to 150. The F1-score based performance of RF model was stable with tree numbers ≥100 (Supplementary Tables S4, S5), thereby this number being used as a final model setting. For k-NN classifier model, 10-fold cross-validation was performed to investigate the optimal number of neighbors from 3 to 103 (only odd numbers; Supplementary Tables S4, S5) by considering the best F1-score given by the model.

### Model performance evaluation metrics

The performance of models was measured by various predictive indices, including accuracy, sensitivity, precision, specificity, and F1-score.


(3) $$\mathrm{Accuracy}=\frac{\mathrm{TP}+\mathrm{TN}}{\mathrm{TP}+\mathrm{TN}+\mathrm{FP}+\mathrm{FN}}$$

(4) $$\mathrm{Sensitivity}=\frac{\mathrm{TP}}{\mathrm{TP}+\mathrm{FN}}$$

(5) $$\mathrm{Precision}=\frac{\mathrm{TP}}{\mathrm{TP}+\mathrm{FP}}$$

(6) $$\mathrm{Specificity}=\frac{\mathrm{TN}}{\mathrm{TN}+\mathrm{FP}}$$

(7) $$F1-\mathrm{score}=\frac{2*\mathrm{Precision}*\mathrm{Sensitivity}}{\mathrm{Precision}+\mathrm{Sensitivity}}$$

*TP* (true positive) is the correct prediction of the interacted DBD and TFBS. *TN* (true negative) is the correct prediction of non-interacted DBD and TFBS. *FP* (false positive) represents the mis-prediction of non-interacted DBD and TFBS. *FN* (false negative) represents the mis-prediction of the interacted DBD and TFBS.

### Model validation with independent ChIP-seq datasets

The constructed Plant-DTI model was validated its predictive performance with 22 independent ChIP-seq data. The TF-TFBS interactions measured in *Arabidopsis thaliana* were retrieved from PCBase (Chow et al., 2019) and used as the gold standard for our test experiment (Supplementary Data 1.1). The consistency of DBD-TFBS interactions from model prediction and ChIP-seq measurement was evaluated. For each positive prediction, similarity of the interacting TFBS motif predicted from Plant-DTI model was examined against the putative TFBS motifs from ChIP-seq experiment and MEME-ChIP (Machanick and Bailey, 2011) using TOMTOM (Gupta et al., 2007).

### Comparison of Plant-DTI performance with PWM (PlantTFDB) and TSPTFBS models

Predictive performance of Plant-DTI model was compared with the other state-of-art methods based on the identical gold standard datasets (10 ChIP-seq and 57 DAP-seq datasets), and the negative data that generated from the low probable TF binding regions in *Arabidopsis thaliana* genome (Supplementary Data 1.2). The two state-of-art methods under investigation were (i) PWM models from PlantTFDB database and (ii) TSPTFBS, the recently published TFBS prediction tool for Plant developed based on Deep learning of Arabidopsis DAP-seq data (Liu et al., 2021). All models were assessed the prediction capability based on sensitivity and specificity indices.

### Prediction of MeSUS1 regulators by Plant-DTI model

To demonstrate the application of Plant-DTI for predicting TF-TG interactions in non-model plant, the promoter sequence of cassava sucrose synthase 1 gene (MeSUS1) was retrieved from the translation start site (TLS) for 2,000 base pairs. The promoter sequence was aligned in a sliding window of 7–15 bp in length and reversed to obtain the complementary strand. These sequences were used as TFBS queries of MeSUS1. DBD amino acid sequences of 1,751 cassava monotypic DBD TF proteins (1,400 genes) were retrieved from PlantTFDB database version 4 (Jin et al., 2017). All possible DNA sequences of MeSUS1 TFBS and amino acid sequence of cassava monotypic DBD TF proteins were paired up together and used as input in the Plant-DTI model for predicting candidate TFs controlling MeSUS1. To obtain high confidence prediction, only the predicted DBD-TFBS interactions with a probability value equal to 1 and found in both RP and RW models were selected as predicted TFs of MeSUS1. Secondly, TF-TFBS interactions of MeSUS1 were predicted relying on the TFBS scan method using MEME FIMO (Grant et al., 2011). The 338 PWMs of cassava TFs from the PlantTFDB database were scanned on the same MeSUS1 gene promoter sequence used as the input of Plant-DTI, with a *q*-value ≤0.05.

## Results

### Overview of Plant-DTI framework

Plant DBD-TFBS Interaction (Plant-DTI) model was a TF-TFBS predictor developed based upon the association of DBD and TFBS in plant species. In the model, TFBS base-preference was created to enhance specificity of the traditional sequence representation in form of binary numbers. The new representation was more information-contented developed based on the probability of having the nucleotide-base at a specific sequential position in the TFBS motif (Supplementary Table S3). The model was preliminary constructed by various combinations of TFBS feature representations (binary and TFBS base-preference for TFBS feature), inclusion of DBD features to TFBS features (combined amino acid binding mode preference for DBD and TFBS features), and a variety of machine learning classifiers with their optimal parameter setting. The final Plant-DTI configuration was proposed according to the outperformed model combination (Supplementary Table S6). In conclusion, the model was constructed by random forest classifier and exhaustive features of both TFBS base-preference and amino acid binding mode preference to varying TFBS lengths from 7 to 15 bp (Figure 1). The performance of Plant-DTI model was further measured against the independent test dataset and was finally assessed with respect to two state-of-art methods based on the same ChIP-seq, DAP-seq, and negative datasets.

### Overall characteristics and performance of Plant-DTI

**Figure 2:**
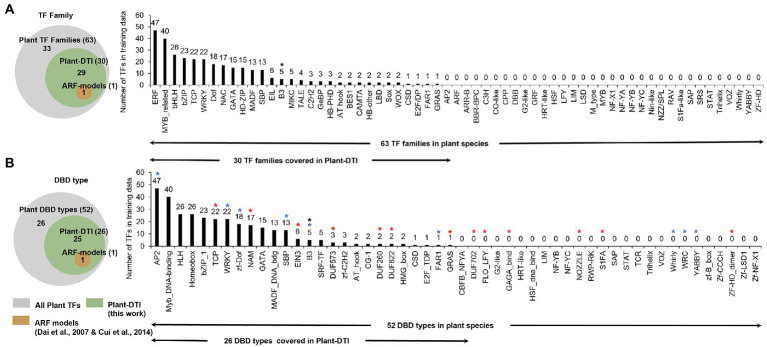
Characteristics of Plant-DTI model based on TF data coverage of 318 TFs in 30 TF families present in 22 plant species **(A)** TF families and **(B)** DNA binding domains (DBDs), red asterisks are plant-specific DBD types (Yamasaki et al., 2013; Lehti-Shiu et al., 2017), blue asterisks are plant-dominant DBD types (Yamasaki et al., 2013; Lehti-Shiu et al., 2017), and black asterisk is the DBD type covered by ARF-models (Dai et al., 2007; Cui et al., 2014).

Plant-DTI model was developed to boost TF-TFBS interaction prediction in plant species. The model was constructed by taking into account experimentally generated interaction data on DBDs of TF proteins and TFBSs in promoters of target genes deposited in CIS-BP database version 1.02 (Weirauch et al., 2014). It covered 318 TFs in 30 TF families found in 22 plant species (Figure 2) with 336 corresponding TFBSs between 7 and 15 base pairs (bp) in length (Supplementary Tables S1, S2). Figure 2 shows that Plant-DTI covers about half of entire TF families (30/63) and DBD types (26/52) observed in plant species. Moreover, Plant-DTI has 7 plant-specific DBD types, TCP, NAM, EIN3, DUF573, DUF260, DUF822, and GRAS (marked by red asterisks in Figure 2), and 6 plant-dominant DBD types, AP2, WRKY, zf-Dof, SBP, B3, FAR1 (marked by blue asterisks in Figure 2; Yamasaki et al., 2013; Lehti-Shiu et al., 2017), enabling the prediction of their specific interaction with promoter sequences of the regulated genes. Thus, Plant-DTI has a broader predictive range over other existing models, which focus mainly on ARF transcription factors in the B3 family (Dai et al., 2007; Cui et al., 2014). The extensive data coverage makes it a promising TF-TFBS predictor for any plant species.

**Figure 3:**
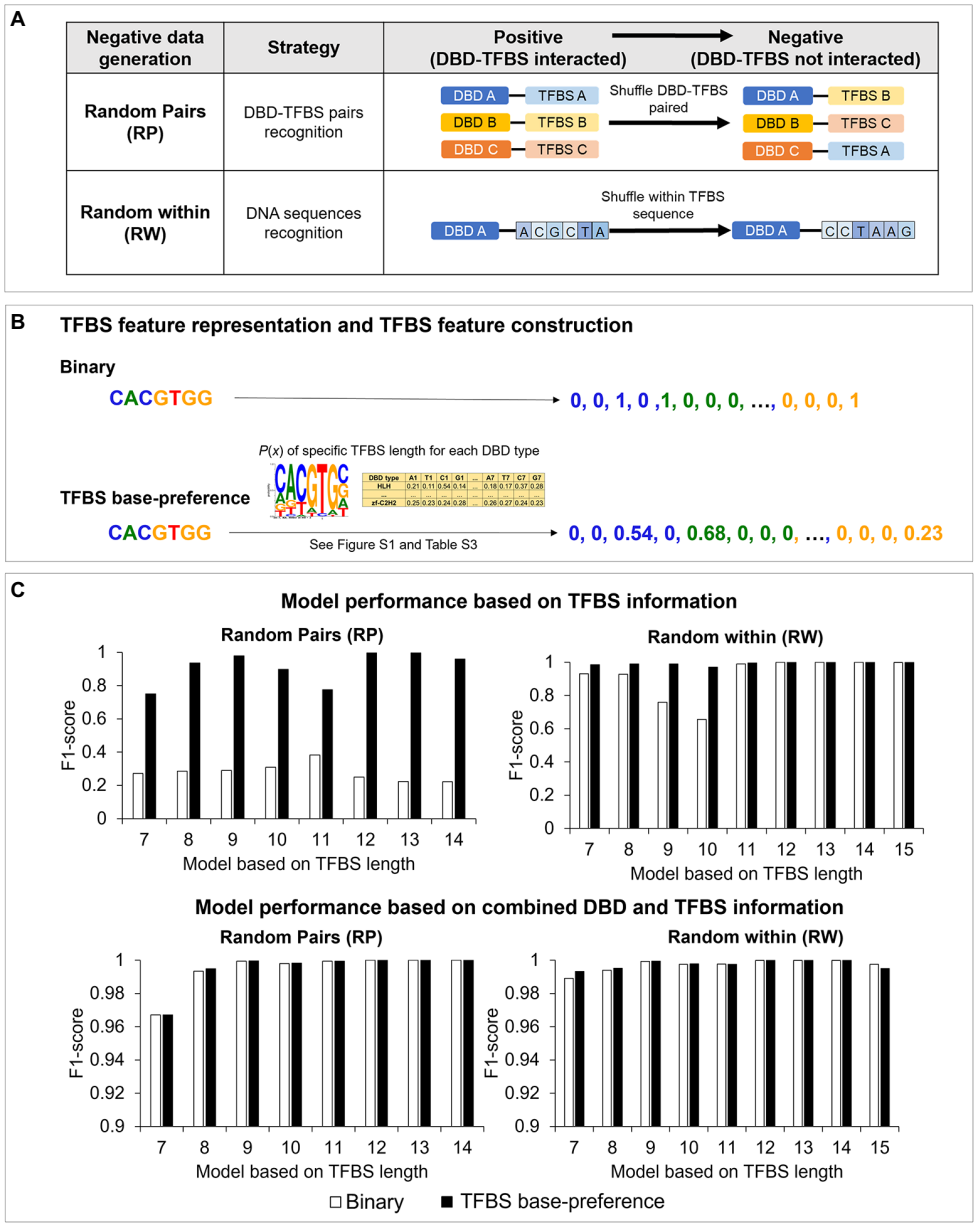
Negative data formation and TFBS feature representations in Plant-DTI model; (A) types of negative data formation: random pairs (RP) and random within (RW), (B) TFBS feature representation: binary (white bar graph) and TFBS base-preference (black bar graph), and (C) the effect of TFBS feature representations on model performance: (top panel) models based on TFBS information and (bottom panel) models based on combined DBD and TFBS information. The study was based on random forest model (RF) algorithm.

The negative training data of Plant-DTI were generated using two different approaches, random pairs (RP) and random within (RW; Supplementary Tables S1, S2). The RP sets were simulated by shuffling known DBD-TFBS pairs to measure their recognition, whereas the RW sets were generated by random permutation of nucleotide base sequences within TFBS to assess cognitive binding of DBDs to specific DNA patterns (Figure 3A). The TFBS sequences were represented by both simple *binary representation* – a series of binary numbers conventionally employed in previously published models (Qian et al., 2006, 2007; Cai et al., 2009; Khamis et al., 2018), and an informative representation – *TFBS base-preference* (Figure 3B). The TFBS base-preference, initiated in this work, proved an improvement on the basic binary representation with respect to the model sensitivity and specificity. The formulated sequence representation incorporated peculiarity of the TFBS motifs that makes it more favored to the DBD than elsewhere. In this regards, nitrogenous bases [adenine (A), cytosine (C), guanine (G), and thymine (T)] in the TFBS sequence were converted into positional probability given by the analysis of base dominance among the known interacting TFBS of each DBD type (see section “Materials and Methods,” TFBS feature representation; Supplementary Figure S1; Supplementary Table S3).

Interestingly, the models with the TFBS base-preference outperformed those with only binary representation in all studied classifiers, Naïve Bayes, Random Forest, and k-nearest neighbor (Figure 3C; Supplementary Figure S2). By training the models with only TFBS information, the models with TFBS base-preference showed much better performances measured in this study (Supplementary Figure S2), especially with the RP negative training dataset (Figure 3C, top-left panel), which showed an F1-score range of 0.7385–0.9971 depending on the TFBS length, compared to the range of 0.223–0.3974 with the binary representation. For the RW negative training dataset, the difference in model performance was more explicit in models with a short TFBS length (<10), and models with the TFBS base-preference still performed better (Figure 3C, top-right panel). The results may be, in effect, the impact of higher information amounts in models having a short TFBS length, especially lengths of 9 and 10 (Supplementary Table S1). It was shown in all predictions that the model with TFBS base-preference had less false positive prediction, while increased model sensitivity compared to those with binary representation. By training the models with combined TFBS and DBD information, performance of models with binary representation was immensely improved, with comparable performances as the TFBS base-preference models in all classifiers used in this study (Figure 3C, bottom panel; Supplementary Figure S2). The models were further optimized based on the hyperparameter of classifier types as presented in Supplementary Tables S4, S5. Supplementary Figure S3 shows that the models based on random forest (RF) are superior to Naïve Bayes (NB) and k-nearest neighbor (k-NN) in terms of accuracy, sensitivity, specificity, and precision in all cases, thereby finally selected for Plant-DTI model.

**Figure 4:**
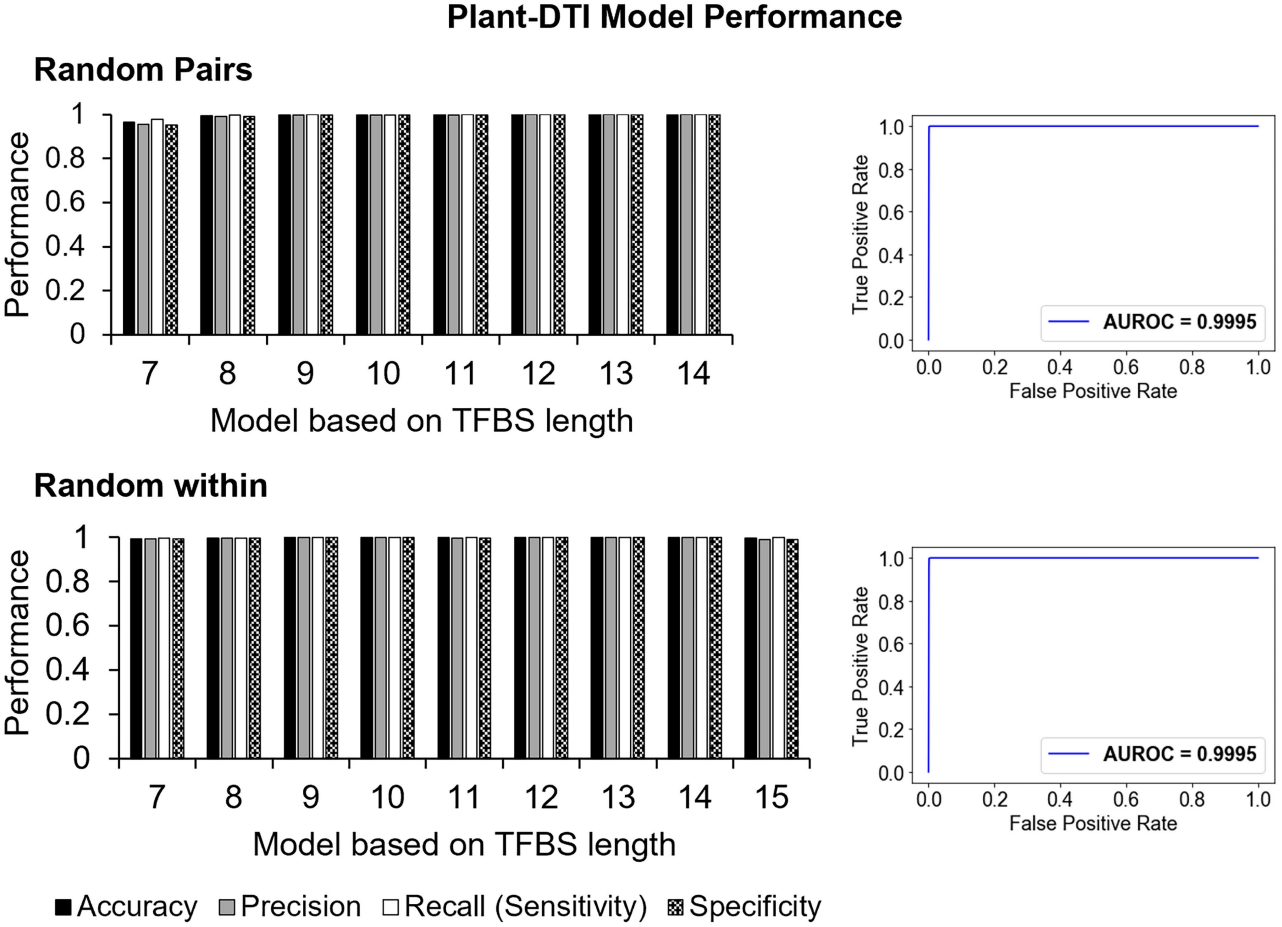
The performance of Plant-DTI model based on accuracy, sensitivity, precision, and ROC curve on 30% hold-out test set for two types of negative data formation: random pairs (RP; top) and random within (RW; bottom).

In summary, the Plant-DTI model was finally configured using the TFBS base-preference representation for TFBS sequences, amino acid binding mode preference to DNA for DBD sequences and RF classifier. The overall process of Plant-DTI model prediction was schematized as in Supplementary Figure S4. Overall performance of the Plant-DTI predictor was assessed through a hold-out method; the results showed that Plant-DTI accurately predicted DBD-TFBS interactions in both RP and RW negative data, with an average accuracy of 99.63% (99.48% for the RP set and 99.75% for the RW set) and average area under receiver operating characteristic (AUROC) curve of 99.95% (Figure 4). Besides the high accuracy, Plant-DTI also showed superior performance on sensitivity (99.79%), precision (99.47%), and specificity (99.47%) with a minimal false-positive rate of 0.54% on average (Figure 4; Supplementary Table S6), suggesting great promise for predicting putative DBD-TFBS interactions.

### Validation of Plant-DTI by ChIP-seq data

**Figure 5:**
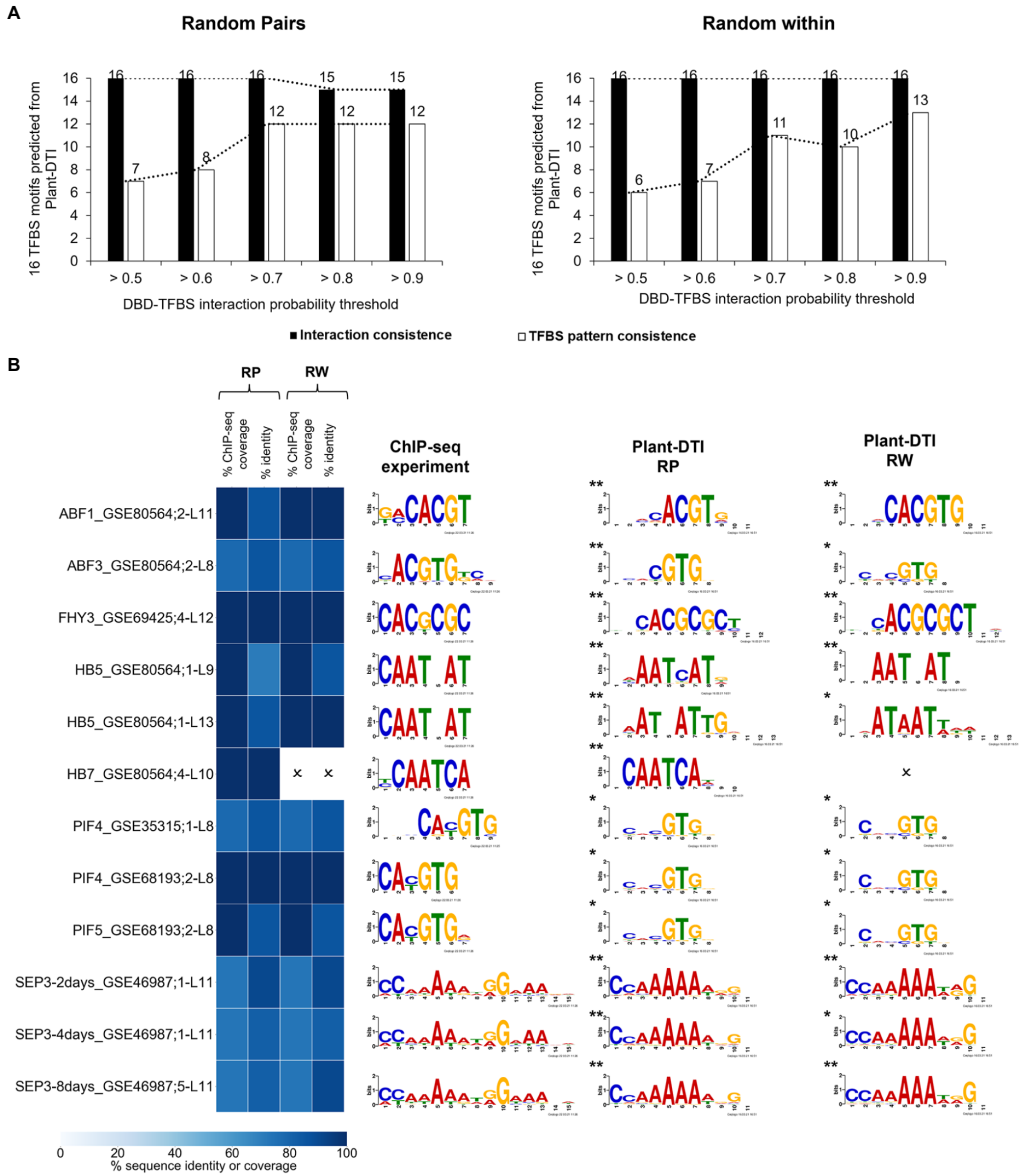
Plant-DTI validation by testing for consistency with experimental DBD-TFBS interactions from independent ChIP-seq data containing same TFs as model training datasets. **(A)** The effect of different probability thresholds on the prediction consistency of the interactions (black bar graph) and TFBS motif patterns (white bar graph). **(B)** Comparison of TFBS motifs predicted at DBD-TFBS interaction probability > 0.7 and TFBS motifs from the ChIP-seq experiment. Asterisks show the statistical significance level of matched motifs from the experimental DBD-TFBS interactions and Plant-DTI predictions, * represents q-value ≤ 0.05 and ** represents q-value ≤ 0.01. Each row shows the information on ChIP-seq motif name (Supplementary Table S7), and L shows model length. Heatmap represents the motif sequence coverage and identity of motifs from ChIP-seq data and Plant-DTI. Cross (O) represents the predicted TFBSs from Plant-DTI are not consistent with ChIP-seq experiment based on criteria; i) percent ChIP-seq coverage < 70 %, ii) percent identity < 70 %, and iii) q-value > 0.05.

Predictability of Plant-DTI was evaluated against DBD-TFBS interactions measured by independent ChIP-seq experiments in the Plant ChIP-seq database (PCBase; Chow et al., 2019). PCBase contains data on 53 TFs including the 14 TFs in Plant-DTI model. According to the data availability, 14 DBD-TFBS interactions (16 interacting pairs, Supplementary Figure S5) of 11 TFs were employed for Plant-DTI validation. Given the identical set of DBD and TFBS sequences, the model could predict 16 corresponding interactions at almost all studied thresholds (ranging from >0.5 to >0.9) in both the RP and RW negative-data models (Figure 5A, black bar). In addition, we also considered the TFBS motifs pattern consistency (Figure 5A, white bar). The 32 putative TFBS motifs obtained along with the 32 TFBS-DBD interactions predicted by the RP and RW models were compared with the 84 patterns of 14 interactions from ChIP-seq data (see section “Materials and Methods”; Supplementary Figure S5). At a probability threshold >0.7, Plant-DTI was able to predict 23 (of the total 32, ~70%) putative TFBS motifs of DBDs that were highly consistent with patterns from the ChIP-seq experiment (identity percentage ≥ 70, coverage percentage ≥ 70, *q*-value ≤0.05; Figure 5B). Higher prediction accuracy and precision could be obtained by increasing the probability threshold, but the improvement was minor at thresholds above 0.7 (Figure 5A; Supplementary Figure S6). Similar results were observed in both the RP and RW negative data models, so 0.7 was set as the default probability threshold for Plant-DTI.

**Figure 6:**
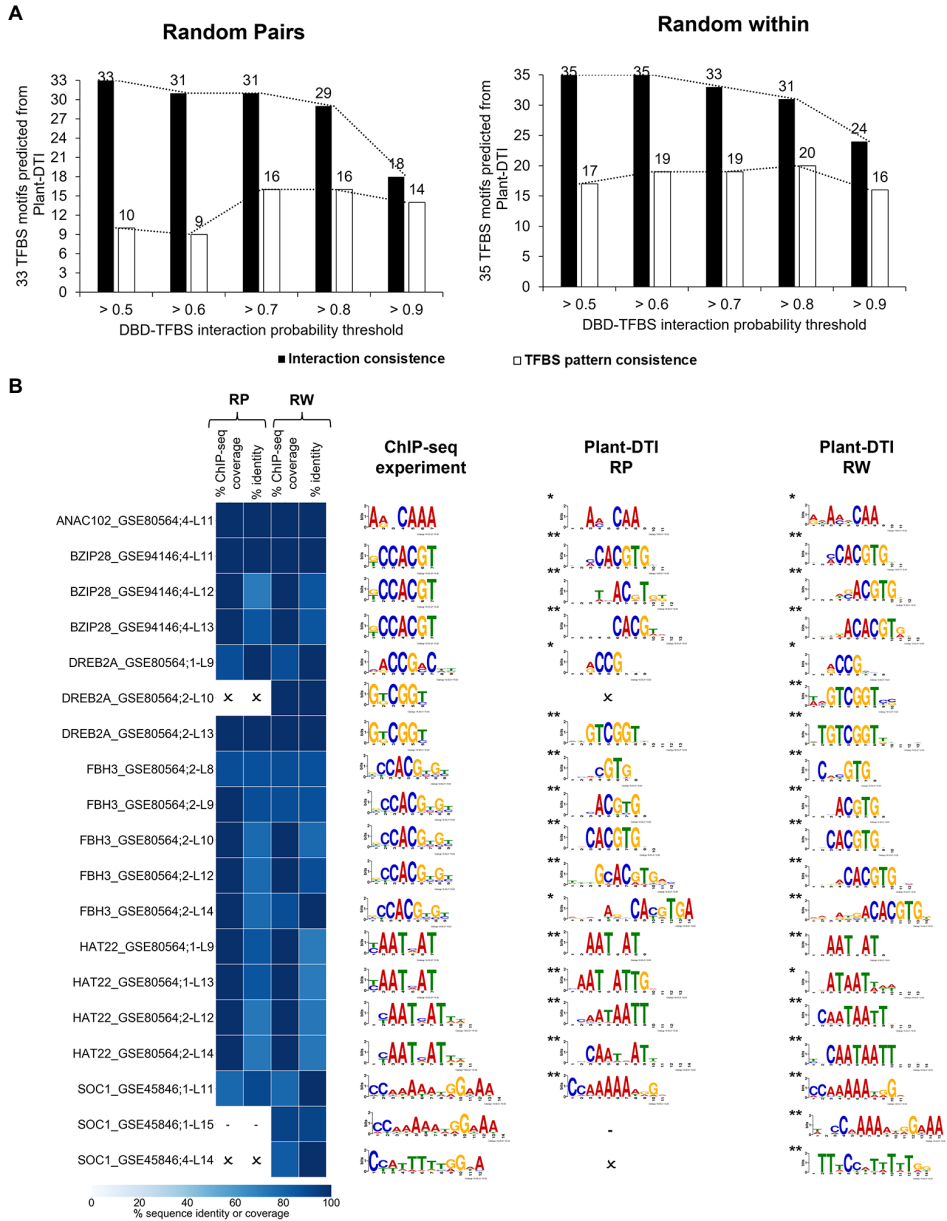
Plant-DTI validation by testing for consistency with experimental DBD-TFBS interactions from independent ChIP-seq data containing same TFs as model training datasets. **(A)** The effect of different probability thresholds on the prediction consistency of the interactions (black bar graph) and TFBS motif patterns (white bar graph). **(B)** Comparison of TFBS motifs predicted at DBD-TFBS interaction probability >0.7 and TFBS motifs from the ChIP-seq experiment. Asterisks show the statistical significance level of matched motifs from the experimental DBD-TFBS interactions and Plant-DTI predictions, * represents *q*-value ≤0.05 and ** represents *q*-value ≤0.01. Each row shows the information on ChIP-seq motif name (Supplementary Table S7), and L shows model length. Heatmap represents the motif sequence coverage and identity of motifs from ChIP-seq data and Plant-DTI. Cross (ϰ) represents the predicted TFBSs from Plant-DTI are not consistent with ChIP-seq experiment based on criteria; (i) percent ChIP-seq coverage <70%, (ii) percent identity <70%, and (iii) *q*-value >0.05. dash (-) represents no predicted TFBSs since out of scope of Plant-DTI.

Furthermore, Plant-DTI was tested to assess its ability to predict DBD-TFBS interactions specific to DBD types. Eight model-exclusive TFs from the ChIP-seq experiment with an identical DBD range as Plant-DTI were employed in this study (Supplementary Figure S7). The putative TFBS motifs for these TFs (8 DBD types consisting of NAM, zf-C2H2, bZIP_1, AP2, HLH, Homeobox, GRAS, and SRF-TF) were inferred based upon the known interaction of TFBS motifs and DBDs in each type. With the same set of DBD and TFBS sequences from the ChIP-seq experiment, the RP model predicted 33 interactions, while the RW model predicted 35 at probability thresholds >0.5. The prediction of DBD-TFBS interactions was highly consistent with the ChIP-seq measurement when using a probability threshold from >0.5 to >0.8 and dramatically declined as the model stringency increased over 0.9, with only 18/33 and 24/35 motifs successfully predicted by the RP and RW models, respectively (Figure 6A, black bar). The 68 putative TFBS motifs predicted from Plant-DTI were compared with the 37 identified in 8 DBD-TFBS interactions by the ChIP-seq data (see section “Materials and Methods”; Supplementary Figure S7). Figure 6B showed that the prediction at probability threshold >0.7 giving optimal results both in terms of DBD-TFBS interactions and the consistency in the associated patterns. This analysis demonstrated that Plant-DTI could be exploited to extrapolate the interaction of TFs and putative TFBS motifs using their DBD-TFBS information. To this end, the contribution of Plant-DTI was highlighted by its potential to predict the interaction of TFs in boarder range. Supplementary Figure S8 shows that up to 70% of TFs in Arabidopsis, rice, maize and cassava were in prediction range of Plant-DTI model, allowing the accessibility to their relationship to TGs.

### Comparison of Plant-DTI performances with state-of-art methods

Predictive performance of Plant-DTI model was contrasted with PWM and TSPTFBS models based on 10 TFs from ChIP-seq and 57 TFs from DAP-seq experiments. These data were obtained from study in a model plant, *Arabidopsis thaliana*. Table 1 shows that Plant-DTI has a merit by having high specificity and sensitivity. In the prediction of ChIP-seq data, Plant-DTI has highest average specificity (99.54%) among the three methods, while showed comparatively high average sensitivity to TSPTFBS (94.75%). The TSPTFBS outperformed in model sensitivity but has weak specificity. PWM was more balanced optimized between sensitivity and specificity, as similar to Plant-DTI, but ours showed more superior performance in both terms. The merit of Plant-DTI was further ensured by comparative analysis of more extended numbers of TFs from DAP-seq experiment. In the prediction of 57 TFs, Plant-DTI still showed upmost average specificity (99.84%) with comparable high average sensitivity (95.36%) with TSPTFBS. The results have supported Plant-DTI as a promising TF-TFBS predictor, especially for plant species.

**Table 1:** Comparison of Plant-DTI, TSPTFBS, and PWM model prediction performance on independence ChIP-seq and DAP-seq dataset.

Experimental data	Performance	Method	Plant-DTI	TSPTFBS	PWM
**Independent ChIP-seq data**	Average sensitivity	Plant-DTI/TSPTFBS/PWM (4 TFs)	94.75	99.97	45.48
		Plant-DTI/PWM (10 TFs)	91.71	–	44.05
	Average specificity	Plant-DTI/TSPTFBS/PWM (4 TFs)	99.54	5.18	95.61
		Plant-DTI/PWM (10 TFs)	99.4	–	97.1
**DAP-seq data**	Average sensitivity	Plant-DTI/TSPTFBS/PWM (57 TFs)	95.36	99.96	70.15
	Average specificity	Plant-DTI/TSPTFBS/PWM (57 TFs)	99.84	10.08	98.13

### Utilization of Plant-DTI to search for putative TFs controlling sucrose synthase 1 gene in cassava

Plant-DTI assisted in filling the gap of knowledge on gene transcriptional regulation. It was employed to predict putative TFs of the cassava sucrose synthase 1 gene (MeSUS1). In the starch biosynthesis pathway, sucrose synthase (SUS) is one of the key sucrolytic enzymes in the reversible conversion of sucrose and uridine diphosphate (UDP) into uridine diphosphate glucose (UDPG) and fructose. These products are essential carbon substrates and intermediate metabolites in a broad range of metabolic pathways in plants (Stein and Granot, 2019). Manipulation of SUS genes is found to affect the yield of storage organs in various plants; for example, overexpression of SUS gene in potato leads to an increase in starch accumulation, ADP-glucose (ADPG) and UDP-glucose (UDPG) contents, and total yield (Baroja-Fernández et al., 2009). Enhancement of SUS activity in maize results in increased ADPG and starch in seed endosperm (Bahaji et al., 2013). In carrot, SUS gene repression decreases UDPG, glucose, fructose, starch, and cellulose contents in taproots (Tang and Sturm, 1999). While the importance of the SUS gene to crop yields is obvious, the underlying transcriptional regulatory mechanism is poorly understood, especially in cassava.

**Figure 7:**
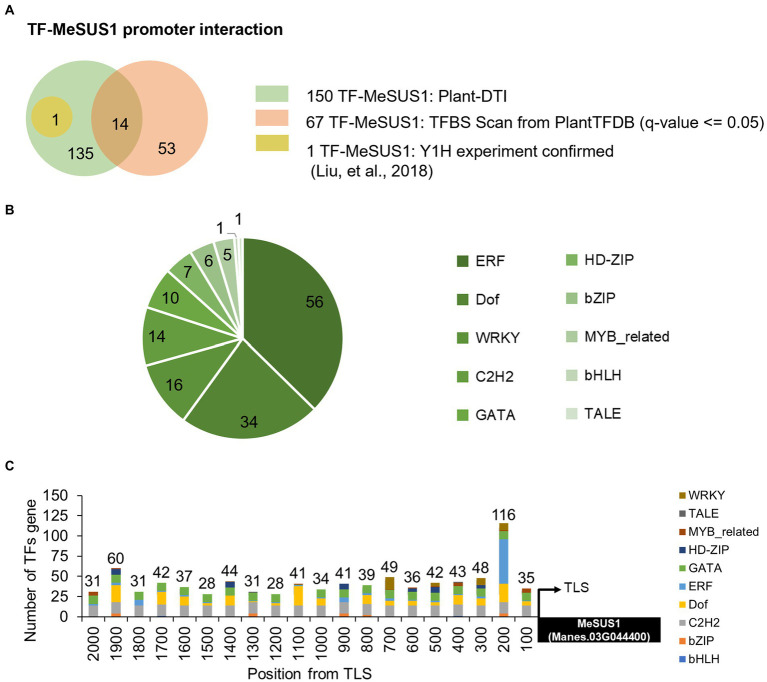
Prediction of cassava SUS1 gene TFs using Plant-DTI model. **(A)** Comparison of TFs of MeSUS1 from Plant-DTI prediction (shown in green), pattern matching-based TFBS scan from PlantTFDB database (shown in orange), and experimentally verified MeERF1 physical binding to MeSUS1 gene (shown in yellow; Liu et al., 2018). **(B)** Pie chart showing the number of predicted TFs categorized by TF family. **(C)** Distribution of TFs and TF families of MeSUS1 promoters predicted by Plant-DTI model.

Cassava (*Manihot esculenta* Crantz) is a well-known staple crop whose starchy roots are the main diets for billions of people (Howeler et al., 2013). As an easy growing plant loaded with carbohydrates, it is a crucial crop for securing food sufficiency by 2050 (Burns et al., 2010). Sucrose synthase enzymes in cassava were encoded by seven MeSUS genes, but only MeSUS1 (Manes.03G044400), MeSUS2 (Manes.01G221900), and MeSUS4 (Manes.16G090600) were found to be highly expressed in storage roots (Huang et al., 2021). MeSUS1 was expressed the most in cassava storage roots among these MeSUS genes (Liu et al., 2018). Plant-DTI predicted 150 cassava TFs belonging to 10 families (WRKY, TALE, MYB-related, HD-ZIP GATA, ERF, Dof, C2H2, bZIP, and bHLH) to interact with the MeSUS1 promoter (Figure 7). The prediction greatly extended the number of putative TFs of MeSUS1 compared to those proposed by the traditional TFBS scan method. About 90% of the predicted TFs (136 of 150 TFs) here were unable to be identified by the TFBS scan (Figure 7A). Among them, MeERF72 TF (Manes.15G009900) was experimentally verified by the yeast-one-hybrid experiment (Liu et al., 2018). Figure 7B shows that MeSUS1 was likely regulated by a large group of the ERF TF family as compared to the others. The ERF TFs are typically involved in various kinds of stress responses, which may help describe the changes in MeSUS1 expression in response to different stress conditions (Zhao et al., 2015; Liu et al., 2018). In addition, the putative binding positions of the predicted TFs (by TF family) were aligned on the MeSUS1 promoter. The results suggested that 200 bp upstream of the translation start site (TLS) was enriched with various TFBS motifs, especially for the ERF family (Figure 7C).

## Discussion

Plants are another domain of life that is of equal importance to microbes and human, but the comprehensive information on transcriptional regulation is lacking. The transcriptional regulatory system of plant species is believed to be rather complicated and contains peculiar elements required for sessile organisms to survive under variable surrounding conditions and perturbations (Shiu et al., 2005; Sullivan et al., 2014; Lehti-Shiu et al., 2017; López-González et al., 2019). In the past decades, computational approaches have helped advance the understanding of transcriptional regulatory systems through the inference of transcriptional regulatory elements and their associations in the mechanistic cascade. Despite this, the findings were mostly confined by the available data in well-studied organisms according to the limitation of the pattern matching-based methods (Chow et al., 2016, 2019; Jin et al., 2017; Puig et al., 2021). Our Plant-DTI predictor resolved the problematic constraint by using a machine learning technique to combine the current knowledge of transcriptional regulation and available data to predict TF-TFBS interactions. The Plant-DTI model covered information on DBD types in plant species and large numbers of DBD-TFBS interactions from experiments, predicting TF-TFBS interactions in roughly half of the TF families (30/63) in plants, including 7 plant-specific DBD (Figure 2). With this data coverage, Plant-DTI represents a promising tool for tackling the lack of TF-TFBS resources in the study of transcriptional regulation in plants.

Plant-DTI was rigorously constructed using various classes of machine learning algorithms. Hyperparameter optimization was performed to ensure optimal predictive performance for each method, and the best model was selected for Plant-DTI (Supplementary Tables S4, S5). DBD information in Plant-DTI model was represented in the form of amino acid mode preference to DNA, which was proven an effective format for predicting DBD-TFBS interaction in humans (Khamis et al., 2018). For TFBS information, we introduced TFBS base-preference as a novel feature reconstruction of the TFBS sequence, which helped increase the prediction power of the model developed based on binary feature representation (Figure 3C). The result highlighted the effectiveness of the TFBS base-preference proposed herein from the probability of each nucleotide in the TFBS motif found specifically binding to a particular DBD type. According to the model configuration, Plant-DTI excelled for all evaluating indices (Figure 4). Validation of Plant-DTI with independent ChIP-seq datasets confirmed its performance in accurately predicting DBD-TFBS interactions and their putative TFBS motifs (Figure 5). Intriguingly, Plant-DTI showed the capability to predict putative TFBSs for any TF members of the modeled DBD types (Figure 6). Moreover, Plant-DTI showed great merit for being a promising low false-positive TF-TFBS predictor. The model had high sensitivity with superior specificity than other state-of-art methods based on prediction of TFs from ChIP-seq and DAP-seq experiment (Table 1).

Application of Plant-DTI model was demonstrated in a study of MeSUS gene transcriptional regulation. It has been reported that the expression of SUS is highly influenced by the exposed environments (Stein and Granot, 2019), especially under stress conditions (Zhao et al., 2015; Sheshadri et al., 2016; Liu et al., 2018). However, the transcriptional regulators responsible for connecting environmental signals to the gene expression remain incompletely known, and almost of all knowledge relies on findings in model organisms such as Arabidopsis. In this work, Plant-DTI proposed at least 150 putative TFs (10 families) regulating MeSUS1 gene expression. There are 136 TFs predicted in addition to the available data from the pattern matching-based method, one of which was previously verified by the Y1H experiment (Liu et al., 2018; Figure 7A). Our result shows that several stress-responsive TF families can regulate MeSUS1, including ERF, WRKY, MYB_related, bHLH (Wang et al., 2016; Figure 7B). TFs in the ERF family that play vital roles as key regulators in responses to many biotic and abiotic stresses (Müller and Munné-Bosch, 2015; Fan et al., 2016; Xie et al., 2019) were mainly predicted. This result may provide insight into the changes in SUS expression in response to stress as reported in plants (Tang et al., 2009; Zhao et al., 2015; Sheshadri et al., 2016). Besides MeSUS1, Plant-DTI helped reduce ambiguous TF-TFBS relationships in plant genomes and was capable of predicting at least 70% of associated TFBSs in rice, maize, and cassava (Supplementary Figure S8).

While Plant-DTI took into account extensive DBD and TFBS information of plant species compared to other models, it is currently limited to monotypic DBD TFs. Incorporating more types of data, features and domains of knowledge, for example, homotypic and heterotypic DBD TFs and structural and physicochemical features of TF proteins, would increase the predictive power of the model (Cai et al., 2009; Khamis et al., 2018). With larger amounts of data, the model may also be upgraded with proficient computing techniques for dealing with high complexity data such as deep learning to conduct robust model prediction. Plant-DTI is expected to provide a promising set of putative TF-TFBS interactions according to its high specificity, nonetheless, experimental verification is still needed to reduce the possible false positive prediction. Recently, a few studies have refined the prediction of TF-TFBS and TF-TG interactions in plants by integrating many types of omics data to gain high confidence in these interactions. However, this approach is constrained by the availability of omics data mostly only in well-studied plants (Brooks et al., 2021; Puig et al., 2021). Once data is available, intensive validation with experiments in plants would leap forward the model capability.

## Conclusion

The Plant-DTI model was developed to predict TF-TFBS interactions in plant species and ultimately help advance the current understanding of transcriptional regulation in major crop plants. It was constructed based on a machine learning approach using combined information on DBD and TFBS from experimentally derived DBD-TFBS interactions in plants, which covered up to half of the plant DBD types, including 7 plant-specific DBD types. In addition, we presented the novel feature construction for TFBS sequence motifs denoted as TFBS base-preference, which proved an improvement on the traditional binary representation. The average accuracy of the model was 99.63%, and validation was done using 22 independent ChIP-seq datasets. Moreover, in comparison with the recent state-of-art methods, Plant-DTI showed the great merit through the high model sensitivity with superior specificity. In addition, Plant-DTI has adjustable DBD-TFBS interaction probability thresholds. This allows users to adjust the prediction result based on the preferred confidence level. Finally, Plant-DTI was used to explore the TFs for MeSUS1, highlighting its prospects for extending the information on TF-TFBS interactions and bolstering the knowledge of transcriptional regulatory systems, especially in crop plants.

## Data availability statement

The datasets presented in this study can be found in online repositories. The names of the repository/repositories and accession number(s) can be found in the article/Supplementary material.

## Author contributions

TS and SK conceived and designed the study. TS, SK, and CN supervised the project. BR analyzed all data and constructed the web application. TS, SK, and BR interpreted, discussed the results, and drafted the manuscript. All authors contributed to the article and approved the submitted version.

## Funding

The research on Plant-DTI development was funded by National Research Council of Thailand [NRCT, Mid-Career research grant (NRCT5-RSA63006-02)], Thailand Science Research and Innovation (TSRI), Basic Research Fund: Fiscal year 2021 under project number FRB640008, and Center for Agricultural Systems Biology, King Mongkut’s University of Technology Thonburi (KMUTT).

## Conflict of interest

The authors declare that the research was conducted in the absence of any commercial or financial relationships that could be construed as a potential conflict of interest.

## Publisher’s note

All claims expressed in this article are solely those of the authors and do not necessarily represent those of their affiliated organizations, or those of the publisher, the editors and the reviewers. Any product that may be evaluated in this article, or claim that may be made by its manufacturer, is not guaranteed or endorsed by the publisher.

## Footnotes

^1^https://bml.kmutt.ac.th/Plant-DTI (Accessed August 05, 2022).

## References

[ref1] Bahaji A. Jose F. Ovecka M. Li J. Baroja-ferna E. Sesma T.. (2013). Enhancing sucrose synthase activity results in increased levels of starch and ADP-glucose in maize (Zea mays L.) seed endosperms. Plant Cell Physiol. 54, 282–294. doi: 10.1093/pcp/pcs180, PMID: 23292602

[ref2] Baroja-Fernández E. Muñoz F. J. Montero M. Etxeberria E. Sesma M. T. Ovecka M.. (2009). Enhancing sucrose synthase activity in transgenic potato (Solanum tuberosum L.) tubers results in increased levels of starch, ADPglucose and UDPglucose and total yield. Plant Cell Physiol. 50, 1651–1662. doi: 10.1093/pcp/pcp108, PMID: 19608713

[ref4] Berger M. F. Bulyk M. L. (2006). “Protein binding microarrays (PBMs) for rapid, high-throughput characterization of the sequence specificities of DNA binding proteins,” in Gene Mapping, Discovery, and Expression. ed. Bina M. (New Jersey: Humana Press), 245–260. 10.1385/1-59745-097-9:245 PMC2690637 16888363

[ref5] Brooks M. D. Juang C. L. Katari M. S. Alvarez J. M. Pasquino A. Shih H. J.. (2021). ConnecTF: a platform to integrate transcription factor-gene interactions and validate regulatory networks. Plant Physiol. 185, 49–66. doi: 10.1093/PLPHYS/KIAA012, PMID: 33631799 PMC8133578

[ref6] Burns A. Gleadow R. Cliff J. Zacarias A. Cavagnaro T. (2010). Cassava: the drought, war and famine crop in a changing world. Sustainability 2, 3572–3607. doi: 10.3390/su2113572

[ref7] Cai Y. He J. Li X. Lu L. Yang X. Feng K.. (2009). A novel computational approach to predict transcription factor DNA binding preference. J. Proteome Res. 8, 999–1003. doi: 10.1021/pr800717y, PMID: 19099508

[ref8] Chen Y. A. Wen Y. C. Chang W. C. (2012). AtPAN: an integrated system for reconstructing transcriptional regulatory networks in Arabidopsis thaliana. BMC Genomics 13, 85. doi: 10.1186/1471-2164-13-85, PMID: 22397531 PMC3314555

[ref9] Chow C. N. Lee T. Y. Hung Y. C. Li G. Tseng K. C. Liu Y. H.. (2019). PlantPAN3.0: a new and updated resource for reconstructing transcriptional regulatory networks from ChIP-seq experiments in plants. Nucleic Acids Res. 47, D1155–D1163. doi: 10.1093/nar/gky1081, PMID: 30395277 PMC6323957

[ref10] Chow C. N. Zheng H. Q. Wu N. Y. Chien C. H. Huang H. Da Lee T. Y.. (2016). PlantPAN 2.0: an update of plant promoter analysis navigator for reconstructing transcriptional regulatory networks in plants. Nucleic Acids Res. 44, D1154–D1160. doi: 10.1093/nar/gkv1035, PMID: 26476450 PMC4702776

[ref11] Cui S. Youn E. Lee J. Maas S. J. (2014). An improved systematic approach to predicting transcription factor target genes using support vector machine. PLoS One 9:e94519. doi: 10.1371/journal.pone.0094519, PMID: 24743548 PMC3990533

[ref12] Dai X. He J. Zhao X. (2007). A new systematic computational approach to predicting target genes of transcription factors. Nucleic Acids Res. 35, 4433–4440. doi: 10.1093/nar/gkm454, PMID: 17576669 PMC1935008

[ref13] Fan W. Hai M. Guo Y. Ding Z. Tie W. Ding X.. (2016). The ERF transcription factor family in cassava: genome-wide characterization and expression analyses against drought stress. Sci. Rep. 6:37379. doi: 10.1038/srep37379, PMID: 27869212 PMC5116755

[ref14] Ferraz R. A. C. Lopes A. L. G. da Silva J. A. F. Moreira D. F. V. Ferreira M. J. N. de Almeida Coimbra S. V. (2021). DNA–protein interaction studies: a historical and comparative analysis. Plant Methods 17:82. doi: 10.1186/s13007-021-00780-z, PMID: 34301293 PMC8299673

[ref15] Franco-Zorrilla J. M. López-Vidriero I. Carrasco J. L. Godoy M. Vera P. Solano R. (2014). DNA-binding specificities of plant transcription factors and their potential to define target genes. Proc. Natl. Acad. Sci. 111, 2367–2372. doi: 10.1073/pnas.1316278111, PMID: 24477691 PMC3926073

[ref16] Grant C. E. Bailey T. L. Noble W. S. (2011). FIMO: scanning for occurrences of a given motif. Bioinformatics 27, 1017–1018. doi: 10.1093/bioinformatics/btr064, PMID: 21330290 PMC3065696

[ref17] Gupta S. Stamatoyannopoulos J. A. Bailey T. L. Noble W. (2007). Quantifying similarity between motifs. Genome Biol. 8:r24. doi: 10.1186/gb-2007-8-2-r24, PMID: 17324271 PMC1852410

[ref18] Han J. Kamber M. Pei J. (2012). Data Mining: Concepts and Techniques, 3rd Edn. Burlington, MA: Morgan Kaufmann Publishers.

[ref19] Howeler R. Lutaladio R. Thomas G. (2013). Save and Grow: Cassava, a Guide to Sustainable Production Intensification. Rome: Food and Agriculture Organization of the United Nations. Available at: http://www.fao.org/ag/save-and-grow/cassava/en/1/index.html (Accessed August 5, 2022).

[ref20] Huang T. Luo X. Fan Z. Yang Y. Wan W. (2021). Genome-wide identification and analysis of the sucrose synthase gene family in cassava (Manihot esculenta Crantz). Gene 769:145191. doi: 10.1016/j.gene.2020.145191, PMID: 33007377

[ref21] Jayaram N. Usvyat D. Martin A. C. R. (2016). Evaluating tools for transcription factor binding site prediction. BMC Bioinformatics 17:547. doi: 10.1186/s12859-016-1298-9, PMID: 27806697 PMC6889335

[ref22] Jin J. Tian F. Yang D. C. Meng Y. Q. Kong L. Luo J.. (2017). PlantTFDB 4.0: toward a central hub for transcription factors and regulatory interactions in plants. Nucleic Acids Res. 45, D1040–D1045. doi: 10.1093/nar/gkw982, PMID: 27924042 PMC5210657

[ref23] Jin J. Zhang H. Kong L. Gao G. Luo J. (2014). PlantTFDB 3.0: a portal for the functional and evolutionary study of plant transcription factors. Nucleic Acids Res. 42, D1182–D1187. doi: 10.1093/nar/gkt1016, PMID: 24174544 PMC3965000

[ref24] Kel A. E. Gößling E. Reuter I. Cheremushkin E. Kel-Margoulis O. V. Wingender E. (2003). MATCH™: a tool for searching transcription factor binding sites in DNA sequences. Nucleic Acids Res. 31, 3576–3579. doi: 10.1093/nar/gkg585, PMID: 12824369 PMC169193

[ref25] Khamis A. M. Motwalli O. Oliva R. Jankovic B. R. Medvedeva Y. A. Ashoor H.. (2018). A novel method for improved accuracy of transcription factor binding site prediction. Nucleic Acids Res. 46:e72. doi: 10.1093/nar/gky237, PMID: 29617876 PMC6037060

[ref26] Kulakovskiy I. V. Vorontsov I. E. Yevshin I. S. Sharipov R. N. Fedorova A. D. Rumynskiy E. I.. (2018). HOCOMOCO: towards a complete collection of transcription factor binding models for human and mouse via large-scale ChIP-Seq analysis. Nucleic Acids Res. 46, D252–D259. doi: 10.1093/nar/gkx1106, PMID: 29140464 PMC5753240

[ref27] Kumar J. Singh S. Singh M. Srivastava P. K. Mishra R. K. Singh V. P.. (2017). Transcriptional regulation of salinity stress in plants: a short review. Plant Gene 11, 160–169. doi: 10.1016/j.plgene.2017.04.001

[ref28] Lai X. Stigliani A. Vachon G. Carles C. Smaczniak C. Zubieta C.. (2019). Building transcription factor binding site models to understand gene regulation in plants. Mol. Plant 12, 743–763. doi: 10.1016/j.molp.2018.10.010, PMID: 30447332

[ref29] Lee W. Park B. Han K. (2017). Sequence-based prediction of putative transcription factor binding sites in DNA sequences of any length. IEEE/ACM Trans. Comput. Biol. Bioinform. 5963, 1461–1469. doi: 10.1109/TCBB.2017.2773075, PMID: 29990126

[ref30] Lehti-Shiu M. D. Panchy N. Wang P. Uygun S. Shiu S. H. (2017). Diversity, expansion, and evolutionary novelty of plant DNA-binding transcription factor families. Biochim. Biophys. Acta. Gene Regul. Mech. 1860, 3–20. doi: 10.1016/j.bbagrm.2016.08.005, PMID: 27522016

[ref31] Li Y. Varala K. Coruzzi G. M. (2015). From milliseconds to lifetimes: tracking the dynamic behavior of transcription factors in gene networks. Trends Genet. 31, 509–515. doi: 10.1016/j.tig.2015.05.005, PMID: 26072453 PMC4558309

[ref32] Liu C. Chen X. Ma P. Zhang S. Zeng C. Jiang X.. (2018). Ethylene responsive factor MeERF72 negatively regulates sucrose synthase 1 gene in cassava. Int. J. Mol. Sci. 19, 1281. doi: 10.3390/ijms19051281, PMID: 29693589 PMC5983797

[ref33] Liu L. Zhang G. He S. Hu X. (2021). TSPTFBS: a Docker image for trans-species prediction of transcription factor binding sites in plants. Bioinformatics 37, 260–262. doi: 10.1093/bioinformatics/btaa1100, PMID: 33416862

[ref34] López-González C. Juárez-Colunga S. Morales-Elías N. C. Tiessen A. (2019). Exploring regulatory networks in plants: transcription factors of starch metabolism. PeerJ 7:e6841. doi: 10.7717/peerj.6841, PMID: 31328026 PMC6625501

[ref35] Luscombe N. M. (2001). Amino acid-base interactions: a three-dimensional analysis of protein-DNA interactions at an atomic level. Nucleic Acids Res. 29, 2860–2874. doi: 10.1093/nar/29.13.2860, PMID: 11433033 PMC55782

[ref36] Machanick P. Bailey T. L. (2011). MEME-ChIP: motif analysis of large DNA datasets. Bioinformatics 27, 1696–1697. doi: 10.1093/bioinformatics/btr189, PMID: 21486936 PMC3106185

[ref37] Marinescu V. D. Kohane I. S. Riva A. (2005). The MAPPER database: a multi-genome catalog of putative transcription factor binding sites. Nucleic Acids Res. 33, D91–D97. doi: 10.1093/nar/gki103, PMID: 15608292 PMC540057

[ref38] Monteiro P. T. Oliveira J. Pais P. Antunes M. Palma M. Cavalheiro M.. (2020). YEASTRACT+: a portal for cross-species comparative genomics of transcription regulation in yeasts. Nucleic Acids Res. 48, D642–D649. doi: 10.1093/nar/gkz859, PMID: 31586406 PMC6943032

[ref39] Müller M. Munné-Bosch S. (2015). Ethylene response factors: a key regulatory hub in hormone and stress signaling. Plant Physiol. 169, 32–41. doi: 10.1104/pp.15.00677, PMID: 26103991 PMC4577411

[ref40] Neph S. Vierstra J. Stergachis A. B. Reynolds A. P. Haugen E. Vernot B.. (2012). An expansive human regulatory lexicon encoded in transcription factor footprints. Nature 489, 83–90. doi: 10.1038/nature11212, PMID: 22955618 PMC3736582

[ref42] Ouwerkerk P. B. F. Meijer A. H. (2001). “Yeast one-hybrid screening for DNA-protein interactions,” in Current Protocols in Molecular Biology Vol. 55. Chichester, UK: Wiley, 12.12.1–12.12.22. 10.1002/0471142727.mb1212s55 18265084

[ref43] Park P. J. (2009). ChIP–seq: advantages and challenges of a maturing technology. Nat. Rev. Genet. 10, 669–680. doi: 10.1038/nrg2641, PMID: 19736561 PMC3191340

[ref44] Pedregosa F. Varoquaux G. Gramfort A. Michel V. Thirion B. Grisel O.. (2011). Scikit-learn: machine learning in python. J. Mach. Learn. Res. 12, 2825–2830. Available at: https://dl.acm.org/doi/10.1145/2786984.2786995 (Accessed August 5, 2022).

[ref45] Puig R. R. Boddie P. Khan A. Castro-Mondragon J. A. Mathelier A. (2021). UniBind: maps of high-confidence direct TF-DNA interactions across nine species. BMC Genomics 22, 482. doi: 10.1186/s12864-021-07760-6, PMID: 34174819 PMC8236138

[ref46] Qian Z. Cai Y. D. Li Y. (2006). A novel computational method to predict transcription factor DNA binding preference. Biochem. Biophys. Res. Commun. 348, 1034–1037. doi: 10.1016/j.bbrc.2006.07.149, PMID: 16899225

[ref47] Qian Z. Lu L. Liu X. J. Cai Y. D. Li Y. (2007). An approach to predict transcription factor DNA binding site specificity based upon gene and transcription factor functional categorization. Bioinformatics 23, 2449–2454. doi: 10.1093/bioinformatics/btm348, PMID: 17623704

[ref48] Riechmann J. L. Heard J. Martin G. Reuber L. -Z. C. Jiang. (2000). Arabidopsis transcription factors: genome-wide comparative analysis among eukaryotes. Science 290, 2105–2110. doi: 10.1126/science.290.5499.2105 11118137

[ref49] Sheshadri S. A. Nishanth M. J. Simon B. (2016). Stress-mediated cis-element transcription factor interactions interconnecting primary and specialized metabolism in planta. Front. Plant Sci. 7:1725. doi: 10.3389/fpls.2016.01725, PMID: 27933071 PMC5122738

[ref50] Shiu S. H. Shih M. C. Li W. H. (2005). Transcription factor families have much higher expansion rates in plants than in animals. Plant Physiol. 139, 18–26. doi: 10.1104/pp.105.065110, PMID: 16166257 PMC1203354

[ref51] Steffens N. O. Galuschka C. Schindler M. Lorenz B. Hehl R. (2004). AthaMap: an online resource for in silico transcription factor binding sites in the Arabidopsis thaliana genome. Nucleic Acids Res. 32, 368D–3372D. doi: 10.1093/nar/gkh017, PMID: 14681436 PMC308752

[ref52] Stein O. Granot D. (2019). An overview of sucrose synthases in plants. Front. Plant Sci. 10:95. doi: 10.3389/fpls.2019.00095, PMID: 30800137 PMC6375876

[ref54] Sullivan A. M. Arsovski A. A. Lempe J. Bubb K. L. Weirauch M. T. Sabo P. J.. (2014). Mapping and dynamics of regulatory DNA and transcription factor networks in *A. thaliana*. Cell Rep. 8, 2015–2030. doi: 10.1016/j.celrep.2014.08.019, PMID: 25220462

[ref55] Tang G. Q. Sturm A. (1999). Antisense repression of sucrose synthase in carrot (Daucus carota L.) affects growth rather than sucrose partitioning. Plant Mol. Biol. 41, 465–479. doi: 10.1023/a:1006327606696, PMID: 10608657

[ref56] Tang T. Xie H. Wang Y. Lu B. Liang J. (2009). The effect of sucrose and abscisic acid interaction on sucrose synthase and its relationship to grain filling of rice (*Oryza sativa* L.). J. Exp. Bot. 60, 2641–2652. doi: 10.1093/jxb/erp114, PMID: 19401410

[ref57] Tian F. Yang D. C. Meng Y. Q. Jin J. Gao G. (2020). PlantRegMap: charting functional regulatory maps in plants. Nucleic Acids Res. 48, D1104–D1113. doi: 10.1093/nar/gkz1020, PMID: 31701126 PMC7145545

[ref58] Turatsinze J. V. Thomas-Chollier M. Defrance M. van Helden J. (2008). Using RSAT to scan genome sequences for transcription factor binding sites and cis-regulatory modules. Nat. Protoc. 3, 1578–1588. doi: 10.1038/nprot.2008.97, PMID: 18802439

[ref59] Wang H. Wang H. Shao H. Tang X. (2016). Recent advances in utilizing transcription factors to improve plant abiotic stress tolerance by transgenic technology. Front. Plant Sci. 7, 67. doi: 10.3389/fpls.2016.00067, PMID: 26904044 PMC4746321

[ref60] Weirauch M. T. Yang A. Albu M. Cote A. G. Montenegro-Montero A. Drewe P.. (2014). Determination and inference of eukaryotic transcription factor sequence specificity. Cell 158, 1431–1443. doi: 10.1016/j.cell.2014.08.009, PMID: 25215497 PMC4163041

[ref61] Wingender E. Dietze P. Karas H. Knüppel R. (1996). TRANSFAC: a database on transcription factors and their DNA binding sites. Nucleic Acids Res. 24, 238–241. doi: 10.1093/nar/24.1.238, PMID: 8594589 PMC145586

[ref62] Xie Z. Nolan T. M. Jiang H. Yin Y. (2019). AP2/ERF transcription factor regulatory networks in hormone and abiotic stress responses in Arabidopsis. Front. Plant Sci. 10, 228. doi: 10.3389/fpls.2019.00228, PMID: 30873200 PMC6403161

[ref63] Yamasaki K. Kigawa T. Seki M. Shinozaki K. Yokoyama S. (2013). DNA-binding domains of plant-specific transcription factors: structure, function, and evolution. Trends Plant Sci. 18, 267–276. doi: 10.1016/j.tplants.2012.09.001, PMID: 23040085

[ref64] Yilmaz A. Mejia-guerra M. K. Kurz K. Liang X. Welch L. Grotewold E. (2011). AGRIS: the Arabidopsis gene regulatory information server, an update. Nucleic Acids Res. 39, D1118–D1122. doi: 10.1093/nar/gkq1120, PMID: 21059685 PMC3013708

[ref65] Yu C. P. Chen S. C. C. Chang Y. M. Liu W. Y. Lin H. H. Lin J. J.. (2015). Transcriptome dynamics of developing maize leaves and genomewide prediction of cis elements and their cognate transcription factors. Proc. Natl. Acad. Sci. 112, E2477–E2486. doi: 10.1073/pnas.1500605112, PMID: 25918418 PMC4434728

[ref66] Yu C. P. Lin J. J. Li W. H. (2016). Positional distribution of transcription factor binding sites in Arabidopsis thaliana. Sci. Rep. 6, 1–7. doi: 10.1038/srep25164, PMID: 27117388 PMC4846880

[ref67] Zhang H. (2004). The optimality of Naive Bayes. In *Proceedings of the Seventeenth International Florida Artificial Intelligence Research Society Conference*, FLAIRS 2004, 562–567.

[ref68] Zhao P. Liu P. Shao J. Li C. Wang B. Guo X.. (2015). Analysis of different strategies adapted by two cassava cultivars in response to drought stress: ensuring survival or continuing growth. J. Exp. Bot. 66, 1477–1488. doi: 10.1093/jxb/eru507, PMID: 25547914 PMC4438449

