# Supplementary material for: Plant-DTI: Extending the landscape of TF protein and DNA interaction in plants by a machine learning-based approach
Source: Front Plant Sci. 2022 Aug 23;13:970018. doi: 10.3389/fpls.2022.970018 (PMC9445498; doi:10.3389/fpls.2022.970018)
Supplement: Supplementary file 1 [file Data_Sheet_1.PDF]

Supplementary Material

for

“Plant-DTI: Extending the landscape of TF protein and DNA interaction in plants by a machine learning-based approach”

Bhukrit Ruengsrichaiya[1], Chakarida Nukoolkit[1,2], Saowalak Kalapanulak[1,3*] and Treenut Saithong[1,3*]

[1] Bioinformatics and Systems Biology Program, School of Bioresources and Technology and School of Information Technology, King Mongkut’s University of Technology Thonburi (Bang KhunThian), Bangkok 10150, Thailand.
[2] School of Information Technology, King Mongkut’s University of Technology Thonburi, ThungKhru, Bangkok 10140, Thailand.
[3]Center for Agricultural Systems Biology, Systems Biology and Bioinformatics Research Group, Pilot Plant Development and Training Institute, King Mongkut’s University of Technology Thonburi (Bang KhunThian), Bangkok 10150, Thailand.

1 Supplementary Data

1.1 Model validation with independent ChIP-seq datasets

1.1.1 ChIP-seq data

The model validation was performed with two independent datasets, (i) ChIP-seq experiments of the same TF gene as training data used for model construction and (ii) ChIP-seq experiments of different TF genes but containing same DBD types as Plant-DTI model. Since TFs containing similar DBDs tend to have similar TFBS motifs, we also validated the model with the later datasets.

For the first case, the 53 TFs corresponded to 66 ChIP-seq experiments were compared to TFs in the training data of Plant-DTI. Among them, there were 14 TFs from 17 independent ChIP-seq experiments in PCBase had the same TF genes as the training data used in Plant-DTI. However, three ChIP-seq experiments corresponded to 3 TFs could not identify over-representative sequences via ChIP-seq data analysis. Thus, there were 11 TFs from 14 independent ChIP-seq experiments in PCBase were selected to predict DBD-TFBS interactions (Figure S5).

For the second case, the 16 DBD types of TFs in PCBase were compared with TF DBD types in Plant-DTI and resulted in 11 consistent DBD types corresponded to 11 ChIP-seq experiments. Among these ChIP-seq experiments, one ChIP-seq experiment was removed out because of non-identification of over-representative sequences via ChIP-seq data analysis. Two ChIP-seq experiments were used in training dataset of Plant-DTI. Thus, the other 8 TFs that were different but contained the same DBDs as the training data of Plant-DTI were also used to predict DBD-TFBS interactions (Figure S7).

1.1.2 TFBS extraction from ChIP-seq data using Plant-DTI model

To predict TFBS motif (in PWM format) of each TF from ChIP-seq data using Plant-DTI, ChIP-seq binding regions were filtered for only high-quality binding regions (q-value <= 0.05) (Chow et al., 2019). High-quality ChIP-seq binding regions were merged if they had replicates. Then, DNA sequences of qualified ChIP-seq binding regions were pre-processed using sliding windows of length L to extract sequences as the query of Plant-DTI model. From the first position of the binding region sequence, windows were moved one nucleotide at a time until the binding region sequence was completed. The number of queries TFBS sequences was resulted as (N-L+1) while N is length of considered binding region sequence and L is TFBS length as the model will test. Both DNA strands were collected, thus, the total number of queries TFBS sequences is 2*(N-L+1). All pre-processed query TFBS sequences were combined with its DBD of TF and represented as feature vectors. These features vectors were used as a query of Plant-DTI model. The model predicted the interactions with various probabilistic thresholds from more than 0.5 to more than 0.9 (> 0.5, > 0.6, …, > 0.9). Finally, the predicted TFBS sequences of each TF were combined to represent PWM of predicted TFBS motifs.

1.1.3 TFBS motifs discovery from ChIP-seq data

Qualified ChIP-seq binding regions were explored for discovering the TFBS motifs using MEME-ChIP (Machanick and Bailey, 2011). The top three PWMs from MEME and top three PWMs from DREME motif discovery algorithms in MEME-ChIP with E-value <= 0.05 were obtained (Chow et al., 2019). From the 11 TFs of 14 independent ChIP-seq experiments in PCBase with the same TF genes as the training data of Plant-DTI, we gained 84 putative TFBS motifs ((3 PWMs from MEME + 3 PWMs from DREME) * 14 ChIP-seq experiments)

(Table S7). For the other ChIP-seq datasets containing eight TF genes that were different from TFs in the training data of Plant-DTI but contained same DBDs as Plant-DTI, 37 putative TFBS motifs were identified and reported in Table S8.

1.1.4 TFBS motifs comparison between putative TFBS motifs discovered from ChIP-seq experiment and predicted TFBS motif from Plant-DTI

The predicted TFBS motifs from Plant-DTI were compared to putative TFBS motifs from the ChIP-seq experiment using TOMTOM (Gupta et al., 2007). The TFBS motifs were considered as significant similarity if they passed three criteria: i) percent coverage ≥ 70 %, ii) percent identity ≥ 70 %, and iii) q-value ≤ 0.05.

1.2 Comparison of Plant-DTI performance with PWM (PlantTFDB) and TSPTFBS models

1.2.1 ChIP-seq and DAP-seq datasets

The 14 independent ChIP-seq datasets corresponded to 11 TFs in PCBase used in Plant-DTI model validation were compared to 619 TFs motifs of *Arabidopsis thaliana* available in PlantTFDB and 265 *Arabidopsis thaliana* TFs in TSPTFBS model. There were four TFs from four independent ChIP-seq experiments that were consistent among three resources, and 10 TFs from 13 independent ChIP-seq experiments were consistent between Plant-DTI and PWM model (Figure S9). To increase the number of comparison cases, the other 57 DAP-seq datasets corresponded to 57 Arabidopsis TFs were further explored and compared (Figure S10). TF binding regions from these ChIP-seq and DAP-seq experiments were considered as positive binding regions while the number of binding regions was various in each TF as shown in Figure S9 and S10.

To evaluate the performance of models, binding regions of ChIP-seq and DAP-seq data were pre-processed to gain the feature vectors for Plant-DTI prediction using the same procedure as Plant-DTI model validation. The predicted DBD-TFBS interactions were considered as true positive (TP) if it can predict as 'interact' within the binding regions of ChIP-seq or DAP-seq data. On the other hand, if model does not predict DBD-TFBS interactions as 'interact' within these binding regions, it will be considered as false negative (FN).

1.2.2 Negative data

To estimate models' specificity, negative datasets were constructed for each ChIP-seq or DAP-seq binding region dataset. It is notably known that it is hard to construct the negative dataset so, in this work we tried to remove regions which contain high possibility to be the TF binding regions based on the information from experimental data. Firstly, the intergenic regions in *Arabidopsis thaliana* genome were subtracted by *Arabidopsis thaliana* DNA hypersensitivity sites (DHSs) from PlantDHS database (Zhang et al., 2016). DHSs are regulatory protein-accessible genomic regions that indicate cis-regulatory enriched areas. Then, to remove the regions that TF can interact to DNA regions, ChIP-seq, DAP-seq data and all TFBS sequences that used in training data of particular TF were used to subtract. The remaining regions were sampled in the same proportion as positive binding regions data and used as negative data for prediction in each model. The number of sampled negative sequences were different due to the different requirements of input in different methods as shown in Table S9 and S10. For Plant-DTI model, the sampled negative regions were pre- processed to gain the feature vectors for Plant-DTI prediction as described previously. The predicted DBD-TFBS interactions were considered as true negative (TN) if it does not predict as 'interact' within the

negative regions. Meanwhile, if model predicts DBD-TFBS interactions as 'interact' within negative region, it will be considered as false positive (FP).

1.2.3 PWM model prediction

The 619 *Arabiodopsis thalina* PWM models were obtained from the PlantTFDB databases and used as input to scan on the ChIP-seq, DAP-seq binding regions and the negative regions (as explained in the previous section) using MEME FIMO with default p-value less than 0.0001 (Grant et al., 2011). If PWM of considered TF found TFBS on its corresponding TF binding regions in ChIP-seq or DAP-seq experiments, the predicted result from PWM model is considered as true positive (TP), whereas if PWM of considered TF found TFBS on negative binding regions, the predicted result from PWM model is considered as false positive (FP).

1.2.4 TSPTFBS model prediction

ChIP-seq, DAP-seq binding region (positive region), and negative regions were used to evaluate TSPTFBS model performance. Since the required query of TSPTFBS was DNA with length at 201 bp, these positive and negative regions were pre-processed by sliding window to 201 bp as described previously. The DNA query obtaining predicted result with probability > 0.5 was considered as 'interact' from TSPTFBS model.

2 Supplementary Figures

TFBS feature representation: **TFBS base-preference**

The probability of the nucleotide base at each position of specific TFBS length for each DBD type, ***Pj*(*X*)**, was calculated based on Equation 1 in **MATERIALS AND METHODS**. For example, at TFBS length 7 of 2 DBD types, HLH and zf-C2H2, the probability of A,T,C,G base at each TFBS position was shown below.

DBD type	A1	T1	C1	G1	...	A7	T7	C7	G7
**HLH**	0.21	0.11	0.54	0.14	...	0.18	0.17	0.37	0.28
**zf-C2H2**	0.25	0.23	0.24	0.28	...	0.26	0.27	0.24	0.23

TFBS feature construction: **TFBS base-preference**

Experimental data

DBD type	TFBS	TFBS length
**HLH**	**CACGTGG**	**7**
**zf-C2H2**	**CATGTGA**	**7**

Conversion to be a training data using TFBS base-preference, ***Pj*(*X*)**

TFBS	A1	T1	C1	G1	...	A7	T7	C7	G7
**CACGTGG**	0	0	0.54	0	...	0	0	0	0.28
**CATGTGA**	0	0	0.24	0	...	0.26	0	0	0

Figure S1. An example of TFBS feature construction, TFBS-base preference.

.

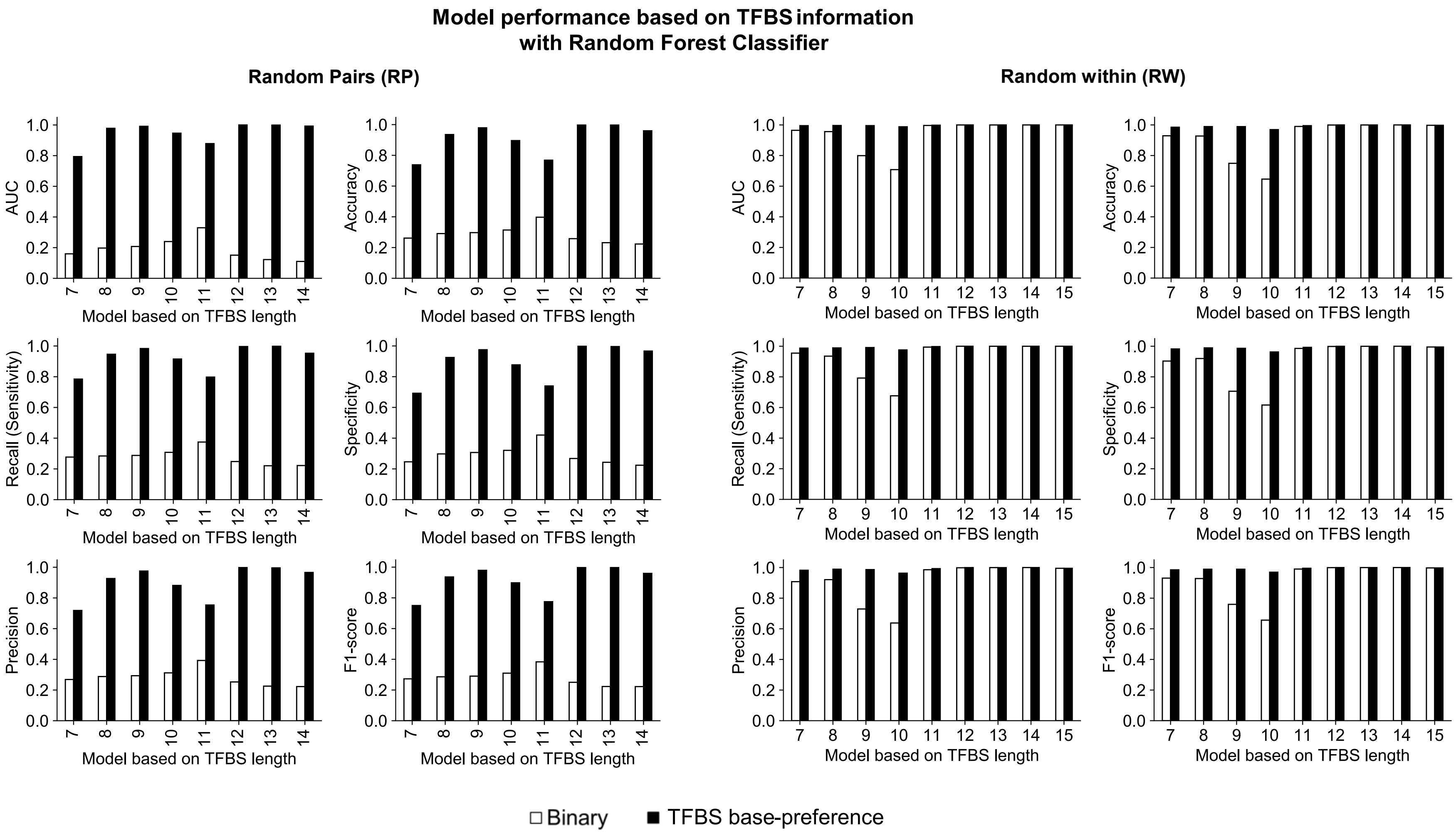

Figure S2. The effect of TFBS feature representations on model performances, AUC, Accuracy, Recall (Sensitivity), Specificity, Precision, and F1-score. Models were constructed based on TFBS information and both DBD-TFBS information with different classifiers, Random Forest, Naïve Bayes, and k-nearest neighbors.

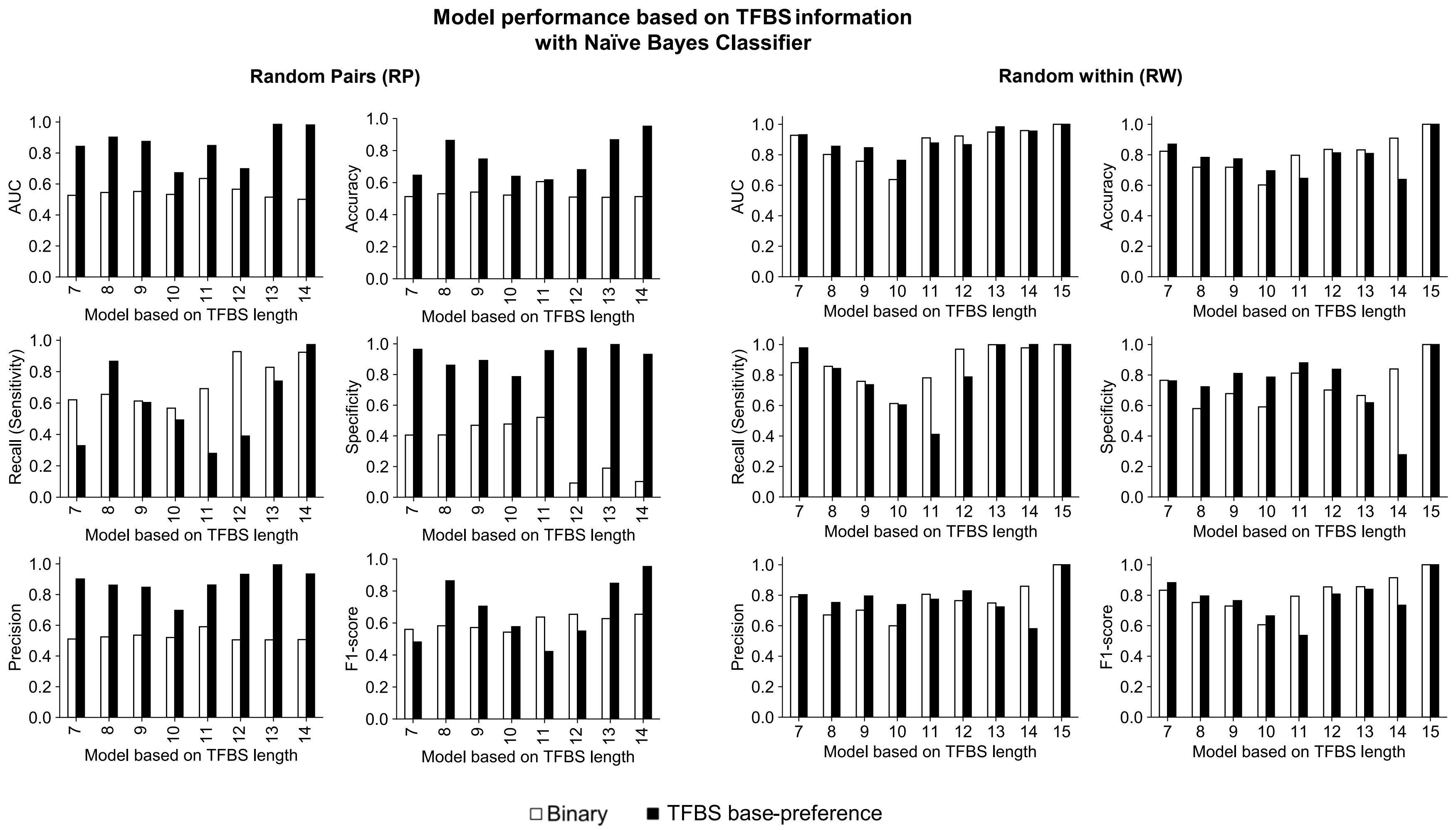

Figure S2 (cont.) The effect of TFBS feature representations on model performances, AUC, Accuracy, Recall (Sensitivity), Specificity, Precision, and F1-score. Models were constructed based on TFBS information and both DBD-TFBS information with different classifiers, Random Forest, Naïve Bayes, and k-nearest neighbors.

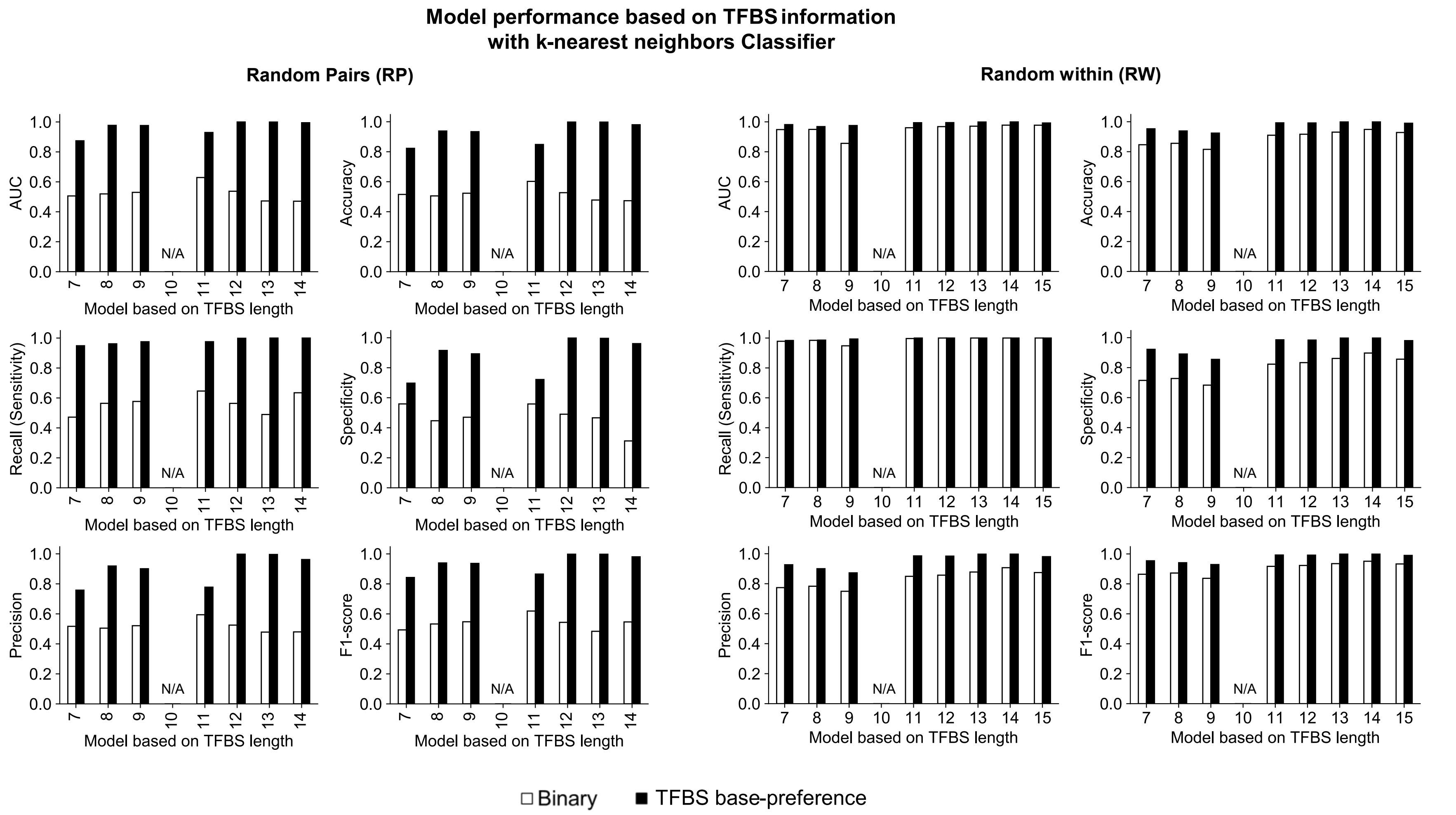

Figure S2 (cont.) The effect of TFBS feature representations on model performances, AUC, Accuracy, Recall (Sensitivity), Specificity, Precision, and F1-score. Models were constructed based on TFBS information and both DBD-TFBS information with different classifiers, Random Forest, Naïve Bayes, and k-nearest neighbors.

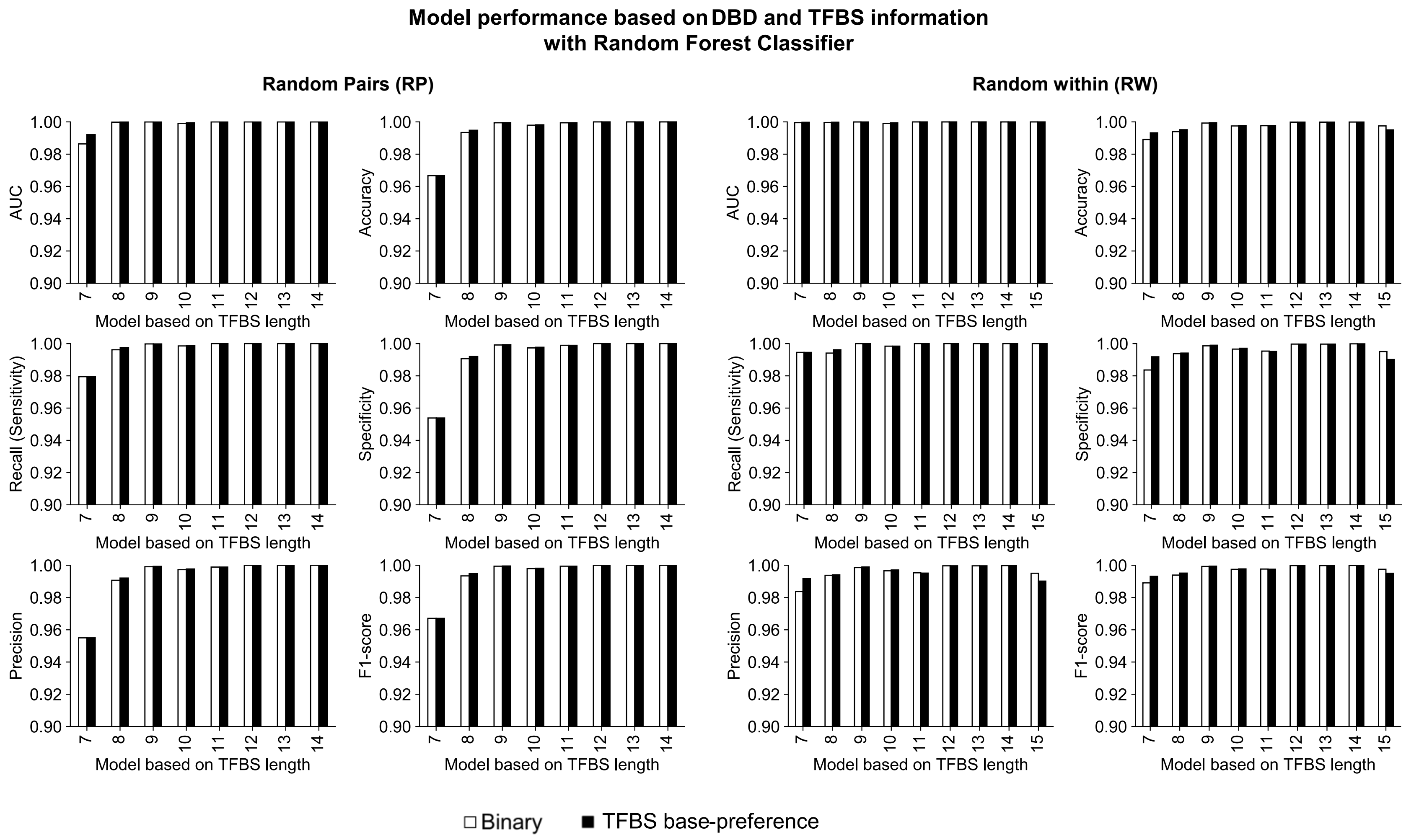

Figure S2 (cont.) The effect of TFBS feature representations on model performances, AUC, Accuracy, Recall (Sensitivity), Specificity, Precision, and F1-score. Models were constructed based on TFBS information and both DBD-TFBS information with different classifiers, Random Forest, Naïve Bayes, and k-nearest neighbors.

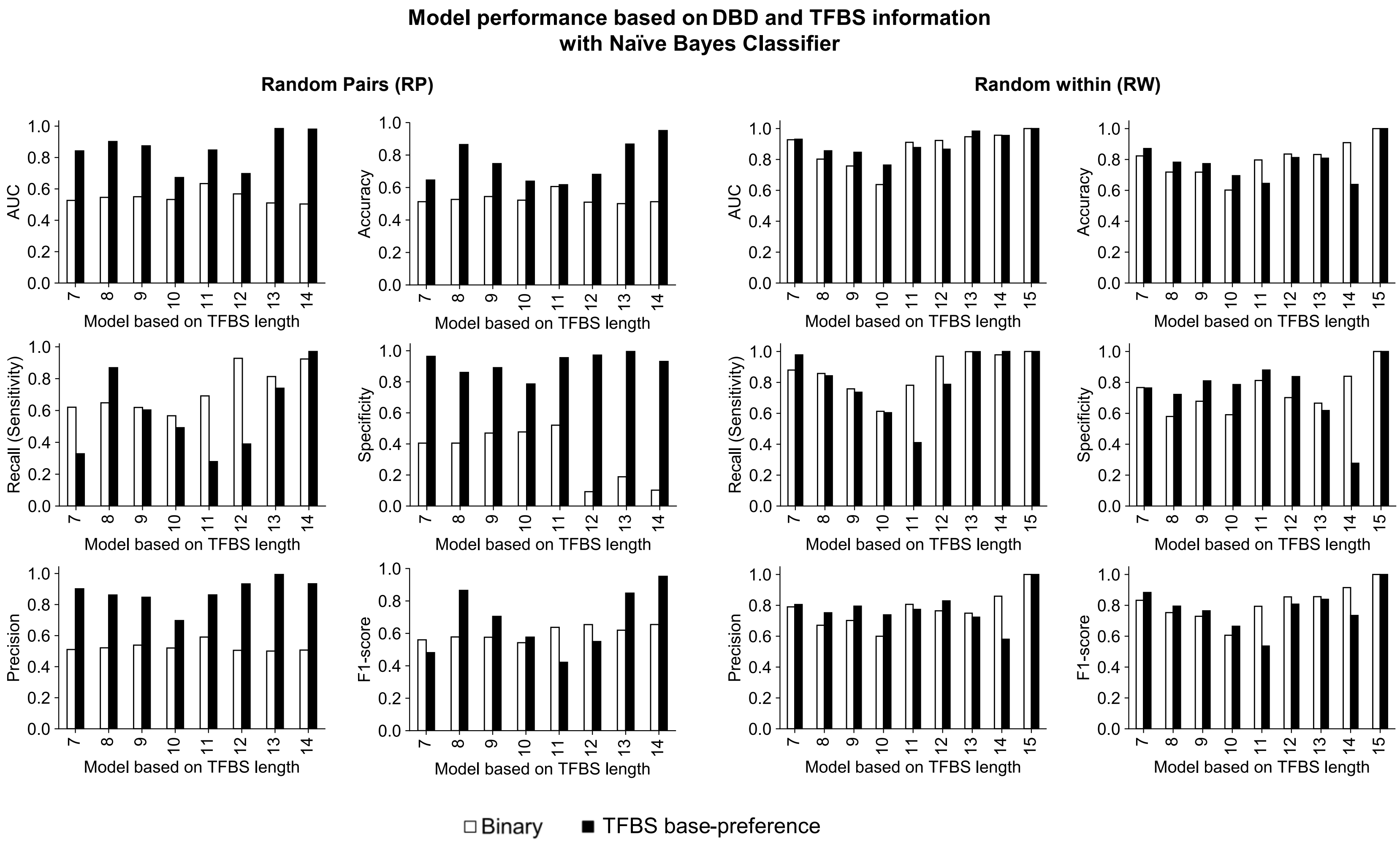

Figure S2 (cont.) The effect of TFBS feature representations on model performances, AUC, Accuracy, Recall (Sensitivity), Specificity, Precision, and F1-score. Models were constructed based on TFBS information and both DBD-TFBS information with different classifiers, Random Forest, Naïve Bayes, and k-nearest neighbors.

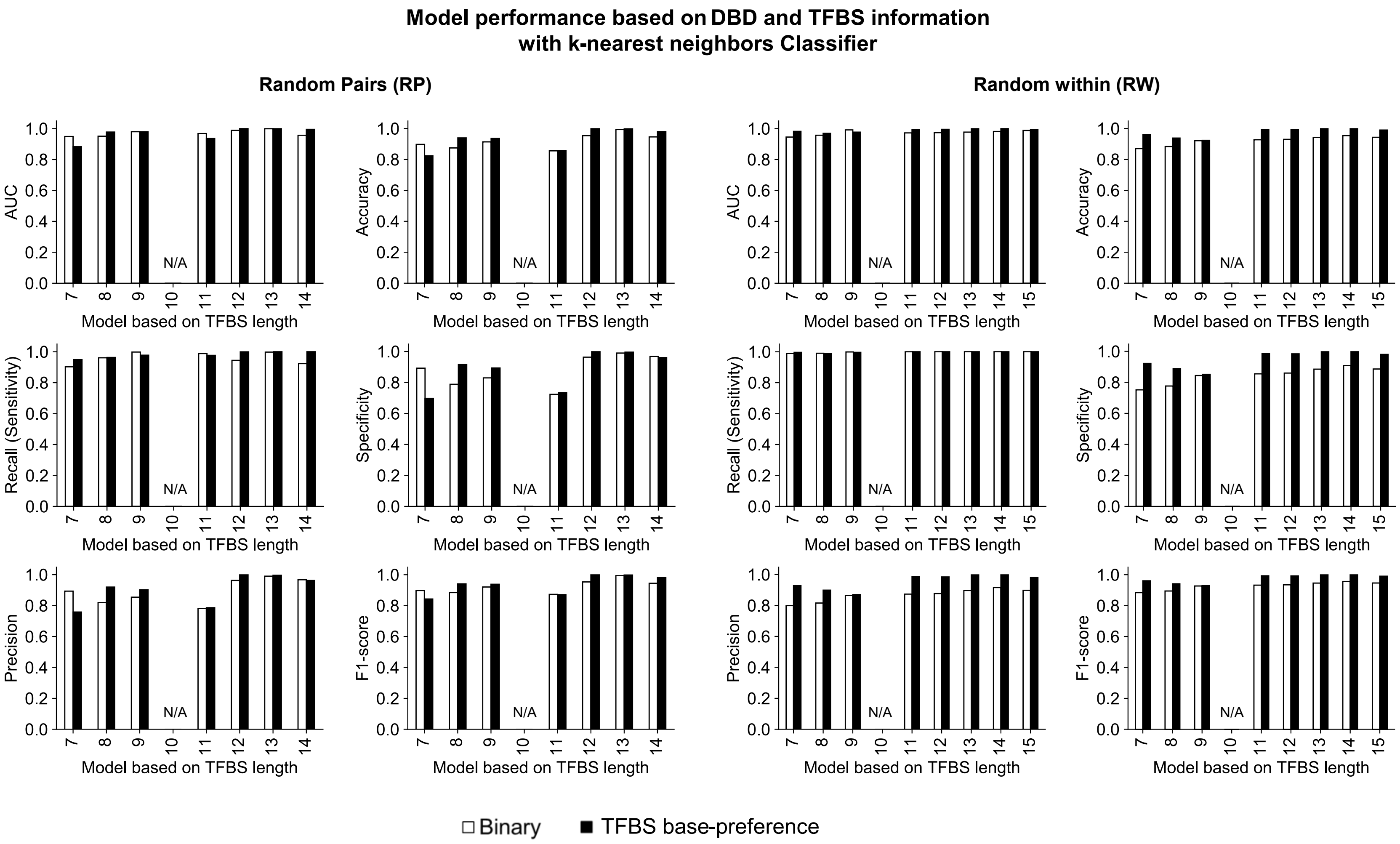

Figure S2 (cont.) The effect of TFBS feature representations on model performances, AUC, Accuracy, Recall (Sensitivity), Specificity, Precision, and F1-score. Models were constructed based on TFBS information and both DBD-TFBS information with different classifiers, Random Forest, Naïve Bayes, and k-nearest neighbors.

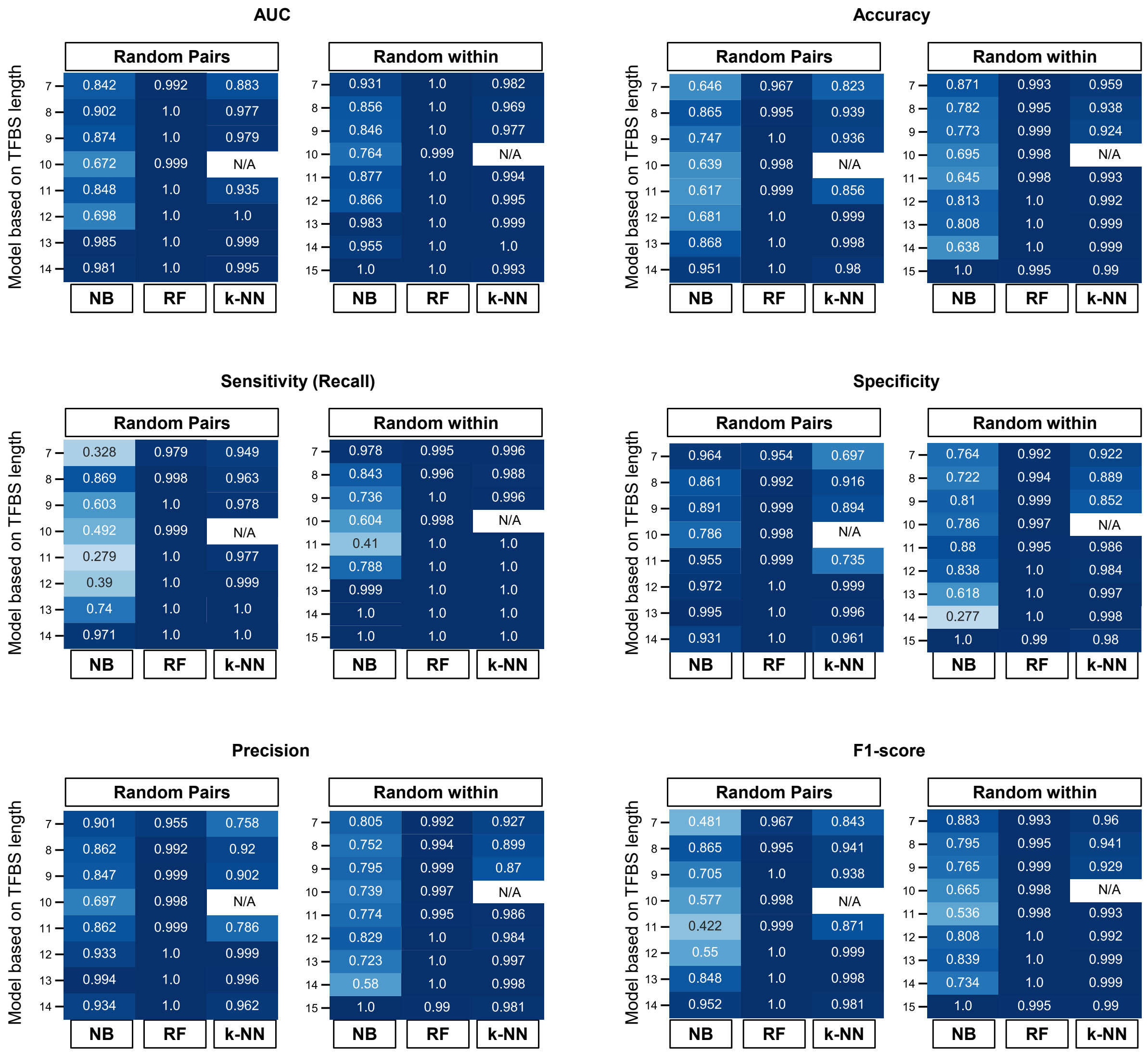

Figure S3. Heatmap showing model performances, AUC, Accuracy, Recall (Sensitivity), Specificity, Precision, and F1-score. Three different classifiers: Naïve Bayes (NB), Random Forest (RF), and k-Nearest Neighbor (k-NN), were used for constructing the model, using DBD and TFBS information, TFBS base-preference to DNA, and amino acid mode of preference to DNA. Each row represents each TFBS length of the model. A darker color shows better model performance.

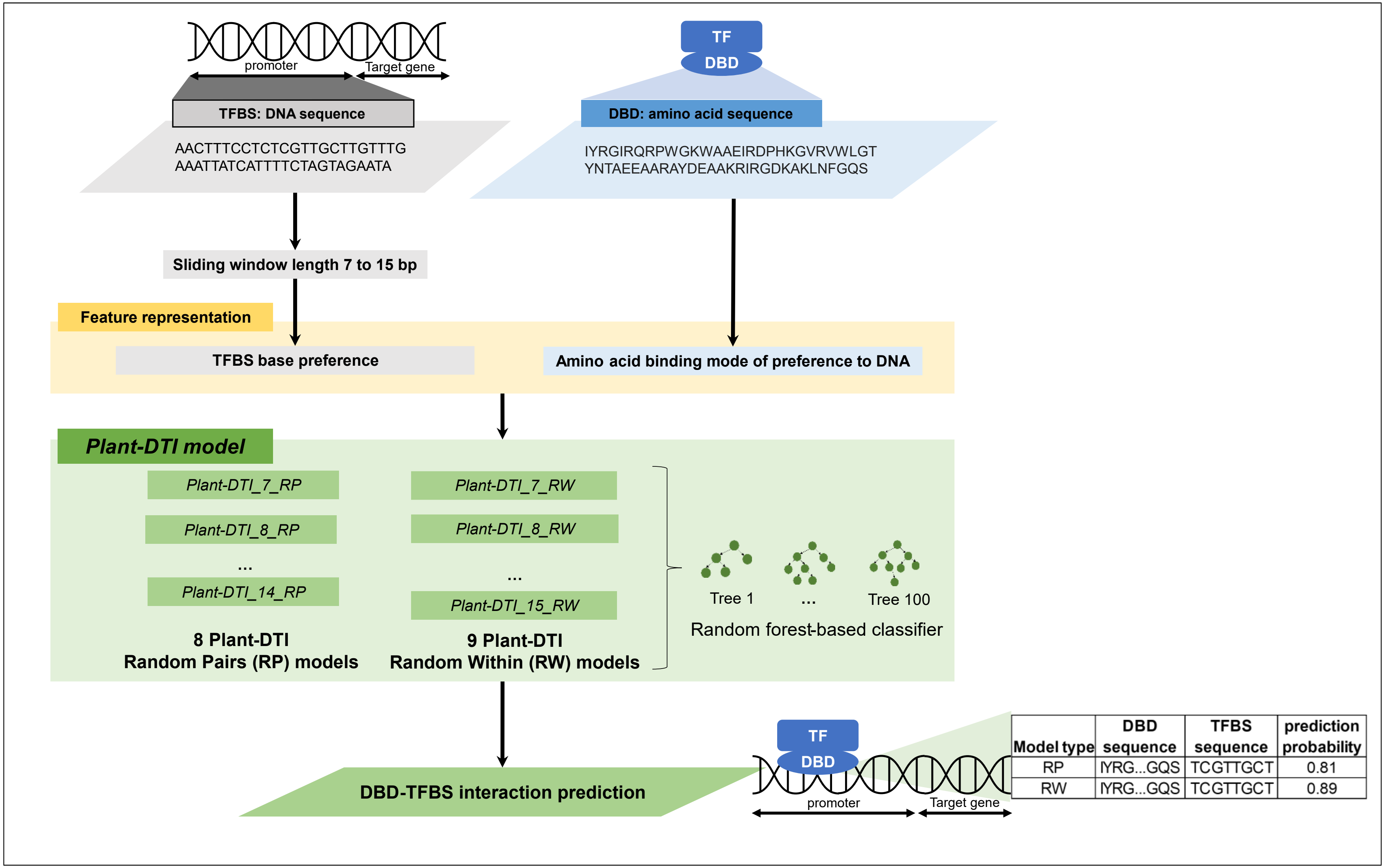

Figure S4. Process of Plant DTI model prediction.

A

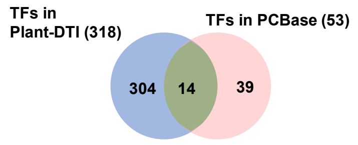

- There are 14 TFs corresponded to 17 ChIP-seq experiments.
- 3 TFs corresponded to 3 ChIP-seq experiments couldn't identify overrepresented sequence.
- Thus **11 TFs** corresponded to **14 ChIP-seq experiments** were considered.

B

ChIP-seq experiment						Plant-DTI model at threshold > 0.7					
No. of ChIP-seq experiment	Gene ID	TF name	experiment ID	DBD type	No. of overrepresented motifs	No. of prediction case	Model based on TFBS length	Interaction consistence		TFBS pattern consistence	
								RP	RW	RP	RW
1	AT1G49720	ABF1	GSE80564	bZIP_1	6	1	10	✓	✓	×	×
						2	11	✓	✓	✓	✓
2	AT4G34000	ABF3	GSE80564	bZIP_1	6	3	8	✓	✓	✓	✓
3	AT3G22170	FHY3	GSE69425	FAR1	6	4	12	✓	✓	✓	✓
4	AT4G01120	GBF2	GSE80564	bZIP_1	6	5	9	✓	✓	×	×
5	AT2G46270	GBF3	GSE80564	bZIP_1	6	6	10	✓	✓	×	×
6	AT5G65310	HB5	GSE80564	Homeobox	6	7	9	✓	✓	✓	✓
						8	13	✓	✓	✓	✓
7	AT2G22430	HB6	GSE80564	Homeobox	6	9	9	✓	✓	✓	✓
8	AT2G46680	HB7	GSE80564	Homeobox	6	10	10	✓	✓	✓	×
9	AT2G43010	PIF4	GSE35315	HLH	6	11	8	✓	✓	✓	✓
10	AT2G43010	PIF4	GSE68193	HLH	6	12	8	✓	✓	×	×
11	AT3G59060	PIF5	GSE68193	HLH	6	13	8	✓	✓	✓	✓
12	AT1G24260	SEP3_2day	GSE46987	SRF-TF	6	14	11	✓	✓	✓	✓
13	AT1G24260	SEP3_4day	GSE46987	SRF-TF	6	15	11	✓	✓	✓	✓
14	AT1G24260	SEP3_8day	GSE46987	SRF-TF	6	16	11	✓	✓	✓	✓

Figure S5. List of 11 TFs (same TF ID) in ChIP-seq experiments used for validating Plant-DTI model. A) Comparison of TFs using as training data in Plant-DTI, and TFs contained in PCBase, B) The results show consistency between Plant-DTI model prediction (at probability threshold > 0.7) and ChIP-seq data analysis using MEME-ChIP (Machanick and Bailey 2011) in terms of interaction and TFBS motif pattern. RP and RW stand for random pairs and random within negative data formation, respectively. Tick (✓) represents consistent prediction of Plant-DTI to ChIP-seq experiment, while cross (×) denotes disagreement.

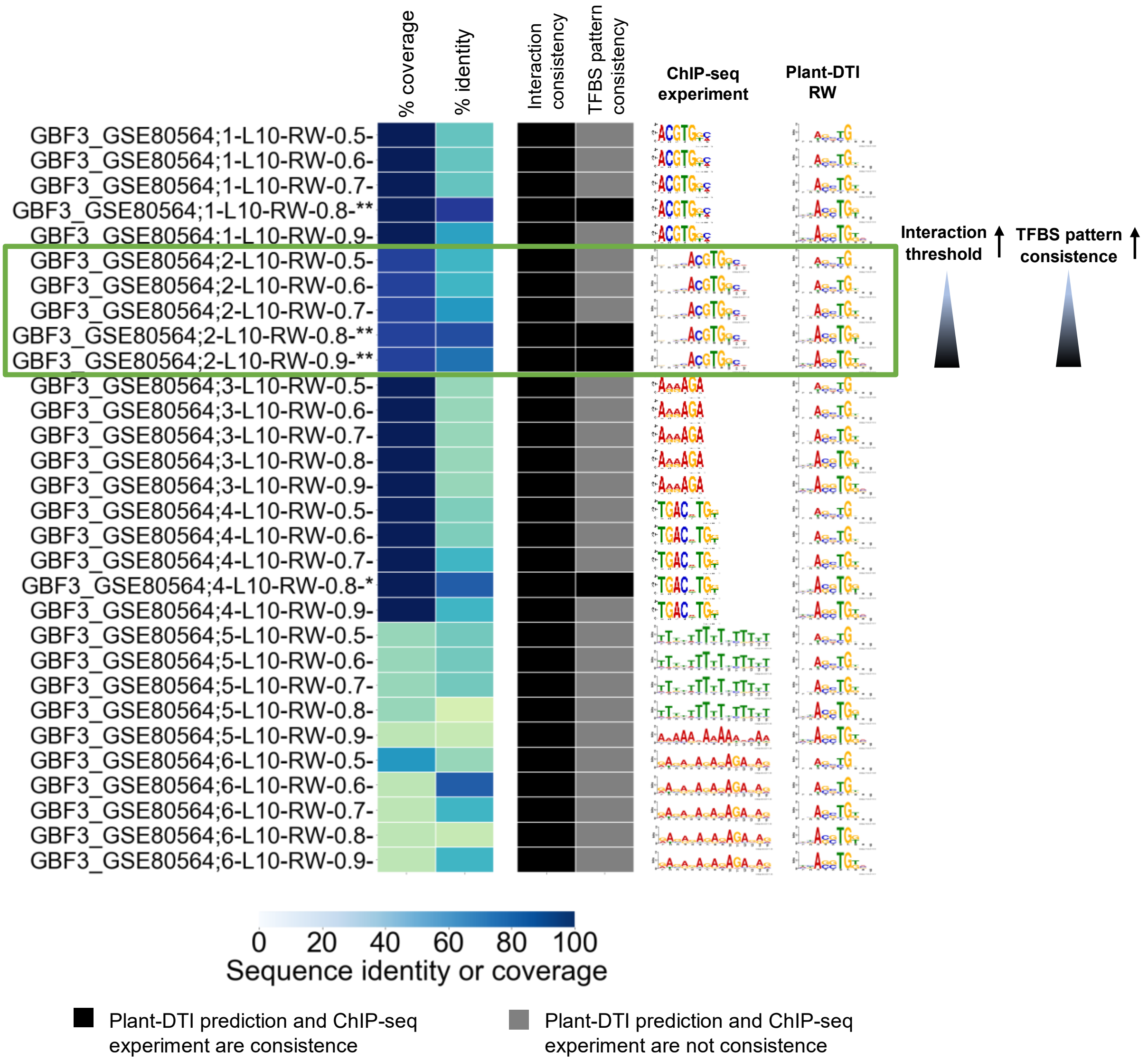

Figure S6. A demonstration of the effect of probability thresholds on DBD-TFBS interaction predicted by Plant-DTI model. The results, regarding sequence identity, coverage, and consistency of predictions, were demonstrated based on GBF3 TF using RW-based model. For Plant-DTI validation, TFBS motifs from the ChIP-seq experiments (maximum of 6 TFBS motifs) were compared to the predicted TFBS motifs for each DBD-TFBS interaction threshold. Matching DBD-TFBS interaction and TFBS motifs pattern were considered consistent. Asterisks show the statistical significance level of matched motifs from experimentally derived DBD-TFBS interactions and Plant-DTI predictions, * represents q-value ≤ 0.05 and ** represents q-value ≤ 0.01. Each prediction (row) was labeled as ChIP-seq motif name (Table S7), model length (L), negative data model, and probability thresholds. Heatmap in blue represents the coverage and identity percentages of the motif sequence predicted by Plant-DTI comparing to the ChIP-seq data. Heatmap in black represents Plant-DTI predictions consistent with the ChIP-seq experiment, while the gray color represents predictions that differ.

A

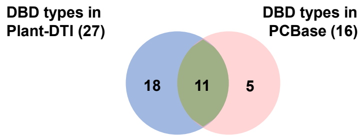

- There are 11 DBD types corresponded to 11 ChIP-seq experiments.
- 1 DBD types corresponded to 1 ChIP-seq experiment couldn't identify overrepresented sequence and 2 DBD types corresponded 2 ChIP-seq experiments were used in dataset of same TF gene as training data used for model construction.
- Thus, **8 DBD types** corresponded to **8 ChIP-seq experiments** were considered.

B

ChIP-seq experiment						Plant-DTI model at threshold > 0.7					
**No. of ChIP-seq experiment**	**Gene ID**	**TF name**	**experiment ID**	**DBD type**	**No. of overrepresented motifs**	**No. of prediction case**	**Model based on TFBS length**	**Interaction consistence**		**TFBS pattern consistence**	
								**RP**	**RW**	**RP**	**RW**
1	AT5G63790	ANAC102	GSE80564	NAM	6	1	8	✓	×	×	×
						2	9	✓	✓	×	×
						3	10	✓	✓	×	×
						4	11	✓	✓	✓	✓
						5	12	-	✓	-	×
2	AT5G67450	AZF1	GSE84483	zf-C2H2	3	6	7	×	✓	×	×
						7	8	✓	×	×	×
3	AT3G10800	BZIP28	GSE94146	bZIP_1	5	8	7	✓	✓	×	×
						9	8	✓	✓	×	×
						10	9	✓	✓	×	×
						11	10	✓	✓	×	×
						12	11	✓	✓	✓	✓
						13	12	✓	✓	✓	✓
						14	13	✓	✓	✓	✓
4	AT5G05410	DREB2A	GSE80564	AP2	6	15	8	✓	✓	×	×
						16	9	✓	✓	✓	✓
						16	10	×	✓	×	✓
						18	13	✓	✓	✓	✓
5	AT1G51140	FBH3	GSE80564	HLH	2	19	7	✓	✓	×	×
						20	8	✓	✓	✓	✓
						21	9	✓	✓	✓	✓
						22	10	✓	✓	✓	✓
						23	12	✓	✓	✓	✓
						24	14	✓	✓	✓	✓
6	AT4G37790	HAT22	GSE80564	Homeobox	6	25	8	✓	✓	×	×
						26	9	✓	✓	✓	✓
						27	10	✓	✓	×	×
						28	11	✓	✓	×	×
						29	12	✓	✓	✓	✓
						30	13	✓	✓	✓	✓
						31	14	✓	✓	✓	✓
7	AT2G01570	RGA	GSE94926	GRAS	5	32	9	✓	✓	×	×
8	AT2G45660	SOC1	GSE45846	SRF-TF	4	33	11	✓	✓	✓	✓
						34	14	✓	✓	×	✓
						35	15	-	✓	-	✓

Figure S7 List of 8 TFs (same DBD type) in ChIP-seq experiments used for validating Plant-DTI model. A) Comparison of TF DBDs covered in Plant-DTI, and TF DBDs contained in PCBase, B) The results show consistency between Plant-DTI model prediction (at probability threshold > 0.7) and ChIP-seq data analysis using MEME-ChIP (Machanick and Bailey 2011) in terms of interaction and TFBS motif pattern. RP and RW stand for random pairs and random within approaches used for negative data formation, respectively. Tick (✓) represents consistent prediction of Plant-DTI to ChIP-seq experiment, while cross (×) denotes disagreement. Dash (-) represents no predicted TFBSs since out of scope of Plant-DTI.

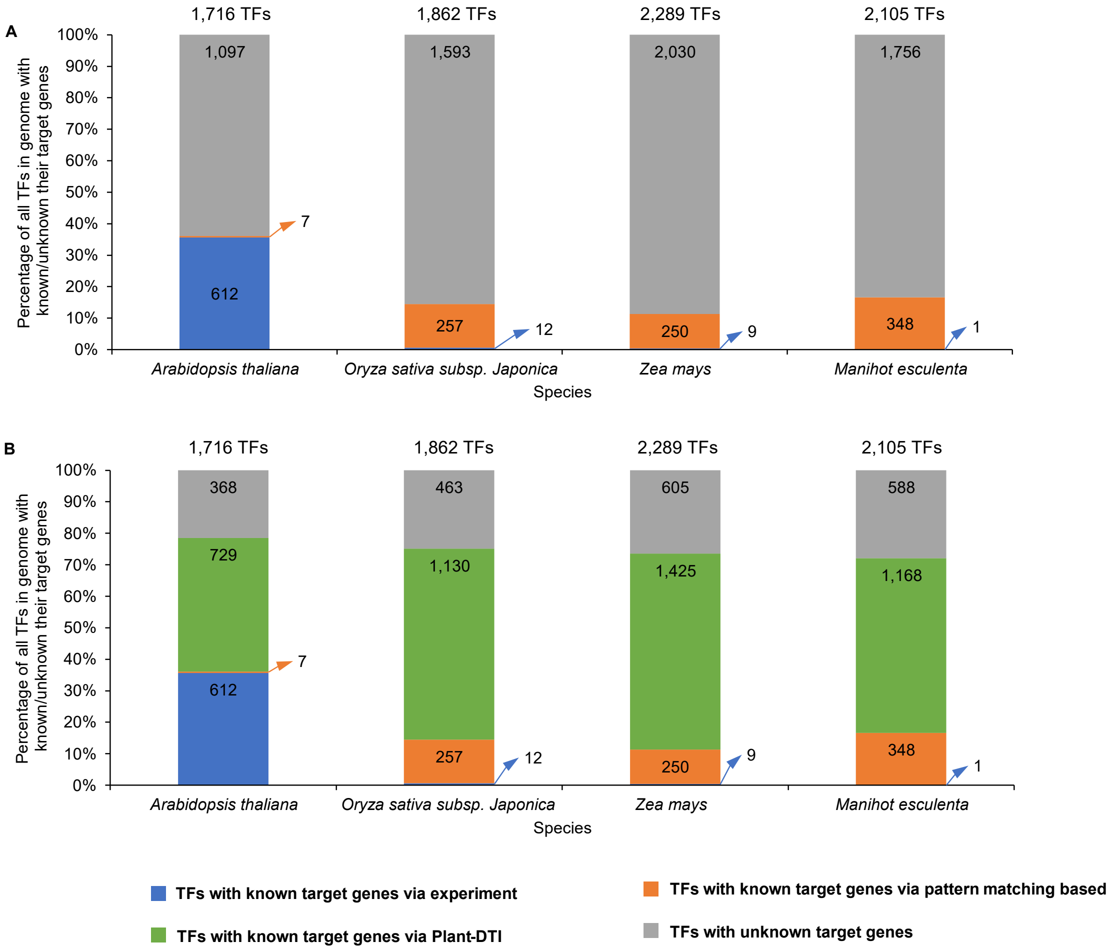

Figure S8 The percentage of all TFs in *Arabidopsis thaliana*, *Oryza sativa* subsp. Japonica, *Zea mays*, and *Manihot esculenta* genomes with known target genes derived by experiments (blue), pattern matching-based approach, TFBS scanning from PlantTFDB (orange), Plant-DTI (green), and with unknown target genes (gray), before Plant-DTI construction (A) and after Plant-DTI construction (B).

A

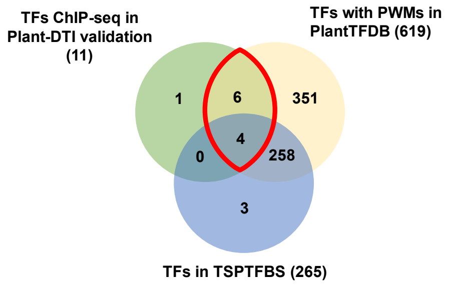

B

Number of TFs	Gene ID	TF name	TF Family	DBD type	experiment ID	Number of ChIP-seq replicates	Plant-DTI based on TFBS length	PWM	TSPTFBS	Number of binding regions
1	AT1G49720	ABF1	bZIP	bZIP_1	GSE80564	3	10<br>11	✓	-	2,925
2	AT4G34000	ABF3	bZIP	bZIP_1	GSE80564	3	8	✓	-	5,954
3	AT3G22170	FHY3	FAR1	FAR1	GSE69425	2	12	✓	-	119
4	AT2G46270	GBF3	bZIP	bZIP_1	GSE80564	3	10	✓	✓	5,949
5	AT5G65310	HB5	HD-ZIP	Homeobox	GSE80564	3	9<br>13	✓<br>✓	✓<br>✓	4,037
6	AT2G22430	HB6	HD-ZIP	Homeobox	GSE80564	3	9	✓	✓	5,934
7	AT2G46680	HB7	HD-ZIP	Homeobox	GSE80564	3	10	✓	✓	5,222
8	AT2G43010	PIF4	bHLH	HLH	GSE35315	1	8	✓	-	3,847
				HLH	GSE68193	3	8	✓	-	2,101
9	AT3G59060	PIF5	bHLH	HLH	GSE68193	2	8	✓	-	661
10	AT1G24260	SEP3_2day	MIKC_MADS	SRF-TF	GSE46987	2	11	✓	-	543
		SEP3_4day		SRF-TF	GSE46987	1	11	✓	-	7,577
		SEP3_8day		SRF-TF	GSE46987	1	11	✓	-	8,430

Figure S9. List of 10 TFs in ChIP-seq experiments used for Plant-DTI model comparison with state-of-art methods. A) Comparison of TFs trained in Plant-DTI, TFs trained in TSPTFBS model, and TFs with PWMs in PlantTFDB, B) Information of TFs in ChIP-seq experiments used for Plant-DTI model comparison with state-of-art methods. Tick (✓) represents TF contained in that method, while dash (-) denotes TF not contained in that method.

A

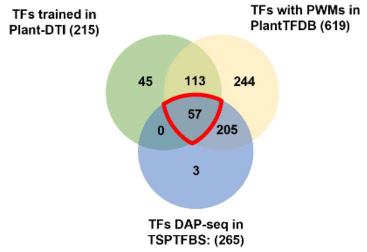

B

Number of TF	Gene ID	TF family	DBD type	TF Name in TSPTFBS	Plant-DTI based on TFBS length	Number of binding regions
1	AT1G45249	bZIP	bZIP_1	ABF2	13	1,902
2	AT5G13180	NAC	NAM	ANAC083	10	3,254
3	AT5G39610	NAC	NAM	ANAC092	9	2,333
4	AT4G00730	HD-ZIP	Homeobox	ANL2	11	3,515
5	AT1G19000	MYB_related	Myb_DNA-binding	AT1G19000	10	3,989
6	AT1G25550	MYB_related	Myb_DNA-binding	AT1G25550	10	1,211
7	AT1G64620	Dof	zf-Dof	AT1G64620	10	5,447
8	AT1G74840	MYB_related	Myb_DNA-binding	AT1G74840	11	3,309
9	AT2G01060	MYB_related	Myb_DNA-binding	AT2G01060	10	2,817
10	AT2G33710	ERF	AP2	AT2G33710	10	6,059
11	AT3G04030	MYB_related	Myb_DNA-binding	AT3G04030	9	6,120
12	AT3G24120	MYB_related	Myb_DNA-binding	AT3G24120	10	10,185
13	AT4G32800	ERF	AP2	AT4G32800	8	1,007
14	AT5G29000	MYB_related	Myb_DNA-binding	AT5G29000	10	1,812
15	AT5G62940	Dof	zf-Dof	AT5G62940	8	12,931
16	AT5G65310	HD-ZIP	Homeobox	ATHB5	9	10,119
16	AT5G65310	HD-ZIP	Homeobox	ATHB5	13	10,119
17	AT2G22430	HD-ZIP	Homeobox	ATHB6	9	3,259
18	AT2G46680	HD-ZIP	Homeobox	ATHB7	10	3,835
19	AT1G59640	bHLH	HLH	bHLH31	9	1,342
20	AT1G69010	bHLH	HLH	BIM2	10	7,691
21	AT1G32150	bZIP	bZIP_1	bZIP68	9	5,497
22	AT4G25470	ERF	AP2	CBF2	8	1,555
23	AT4G25480	ERF	AP2	CBF3	10	2,764
24	AT3G47500	Dof	zf-Dof	cdf3	9	3,570
25	AT1G29160	Dof	zf-Dof	COG1	8	4,873
26	AT4G27950	ERF	AP2	CRF4	8	1,503
27	AT1G18330	MYB_related	Myb_DNA-binding	EPR1	9	8,863
28	AT3G23240	ERF	AP2	ERF1	8	1,346
28	AT3G23240	ERF	AP2	ERF1	10	1,346
29	AT5G51190	ERF	AP2	ERF105	8	5,552
30	AT1G28370	ERF	AP2	ERF11	10	1,541
31	AT2G44840	ERF	AP2	ERF13	8	3,942
32	AT2G31230	ERF	AP2	ERF15	8	6,378
33	AT1G50640	ERF	AP2	ERF3	8	2,064
34	AT3G15210	ERF	AP2	ERF4	8	3,421
35	AT3G20310	ERF	AP2	ERF7	10	6,398
36	AT1G53170	ERF	AP2	ERF8	10	4,004
37	AT1G08010	GATA	GATA	GATA11	8	1,491
38	AT3G06740	GATA	GATA	GATA15	9	1,043
39	AT2G46270	bZIP	bZIP_1	GBF3	10	4,343
40	AT5G04410	NAC	NAM	NAC2	12	3,361
41	AT2G46770	NAC	NAM	NST1	10	3,891
42	AT5G60850	Dof	zf-Dof	OBP4	10	5,063
42	AT5G60850	Dof	zf-Dof	OBP4	11	5,063
43	AT1G43160	ERF	AP2	RAP2.6	8	1,564
44	AT1G20980	SBP	SBP	SPL14	9	1,211
45	AT3G15270	SBP	SBP	SPL5	9	2,127
46	AT5G67580	MYB_related	Myb_DNA-binding	TBP3	8	2,618
47	AT5G65210	bZIP	bZIP_1	TGA1	10	3,574
47	AT5G65210	bZIP	bZIP_1	TGA1	11	3,574
48	AT5G06950	bZIP	bZIP_1	TGA2	10	5,456
49	AT5G10030	bZIP	bZIP_1	TGA4	10	1,868
50	AT3G12250	bZIP	bZIP_1	TGA6	10	5,679
51	AT1G30650	WRKY	WRKY	WRKY14	10	2,399
52	AT2G23320	WRKY	WRKY	WRKY15	10	2,944
53	AT4G31800	WRKY	WRKY	WRKY18	10	6,642
54	AT4G01250	WRKY	WRKY	WRKY22	10	6,105
55	AT5G52830	WRKY	WRKY	WRKY27	9	1,432
56	AT5G24110	WRKY	WRKY	WRKY30	10	1,994
57	AT5G13080	WRKY	WRKY	WRKY75	8	8,136

Figure S10 List of 57 TFs in DAP-seq experiments used for Plant-DTI model comparison with state-of-art methods. A) Comparison of TFs trained in Plant-DTI, TFs with PWMs in PlantTFDB, and TFs DAP-seq experiment trained in TSPTFBS model, B) Information of TFs in DAP-seq experiments used for Plant-DTI model comparison with state-of-art methods.

3 Supplementary Tables

Table S1. Details of plant DBD-TFBS interaction data in Plant-DTI model retrieved from CIS-BP version 1.02.

TFBS length	DBD type	Number of TFs gene	Number of TFBSs	Number of positive interactions	Number of interactions data for model (positive + negative interactions data)	Number of training set	Number of test set
	Random DBD-TFBS pairs (RP)						
**7**	HLH, bZIP_1, zf-C2H2, zf-Dof, GATA, SBP	9	9	649	1,298	908	390
**8**	Homeobox, AP2, bZIP_1, B3, HLH, AT_hook, zf-C2H2, CSD, zf-Dof, EIN3, GATA, MADF_DNA_bdg, Myb_DNA-binding,NAM, SBP, DUF573, TCP, WRKY	85	85	9,631	19,262	13,483	5,779
**9**	Homeobox, SBP, AP2, AT_hook, B3, HLH, bZIP_1, CG-1, zf-Dof, EIN3, GATA, GRAS, Myb_DNA-binding, NAM, HMG_box, DUF573, TCP, WRKY	48	48	151,485	302,970	212,079	90,891
**10**	HLH, AP2, B3, bZIP_1, zf-Dof, MADF_DNA_bdg, E2F_TDP, EIN3, GATA, Homeobox, DUF260, Myb_DNA-binding, NAM, SBP, HMG_box, DUF573, TCP, WRKY	144	146	1,734,704	3,469,408	2,428,585	1,040,823
**11**	bZIP_1, zf-Dof, B3, EIN3, GATA, Homeobox, SRF-TF,Myb_DNA-binding, NAM, DUF822, WRKY	21	21	30,810	61,620	43,134	18,486
**12**	Homeobox, bZIP_1, HLH, FAR1, SBP	8	10	4,654	9,308	6,515	2,793
**13**	Homeobox, AP2, bZIP_1, MADF_DNA_bdg, Myb_DNA-binding	6	6	31,906	63,812	44,668	19,144
**14**	Homeobox, HLH, SRF-TF	4	4	1,696	3,392	2,374	1,018
	**Total**	313	330	1,965,535	3,931,070	2,751,746	1,179,324

Table S1. (Cont.) Details of plant DBD-TFBS interaction data in Plant-DTI model retrieved from CIS-BP version 1.02.

TFBS length	DBD type	Number of TFs gene	Number of TFBSs	Number of positive interactions	Number of interactions data for model (positive + negative interactions data)	Number of training set	Number of test set
			Random nucleotides within TFBS sequence (RW)				
**7**	HLH, bZIP_1, zf-Dof, GATA, SBP	11	11	2,441	4,882	3,417	1,465
**8**	Homeobox, AP2, bZIP_1, B3, HLH, AT_hook, zf-C2H2, zf-Dof, EIN3, GATA, MADF_DNA_bdg, Myb_DNA-binding, NAM, SBP, DUF573, TCP, WRKY	84	84	9,627	19,254	13,477	5,777
**9**	Homeobox, SBP, AP2, B3, HLH, bZIP_1, CG-1, zf-Dof, EIN3, GATA, GRAS, Myb_DNA-binding, NAM, HMG_box, DUF573, TCP, WRKY	47	47	133,989	267,978	187,584	80,394
**10**	HLH, AP2, B3, bZIP_1, zf-Dof, MADF_DNA_bdg, E2F_TDP, EIN3, GATA, Homeobox, DUF260, Myb_DNA-binding, NAM, SBP, HMG_box, DUF573, TCP, WRKY	139	141	999,216	1,998,432	1,398,902	599,530
**11**	bZIP_1, zf-Dof, B3, EIN3, GATA, Homeobox, SRF-TF,Myb_DNA-binding, NAM, DUF822, WRKY	21	21	30,810	61,620	43,134	18,486
**12**	Homeobox, bZIP_1, HLH, FAR1, NAM, SBP	9	11	10,798	21,596	15,117	6,479
**13**	Homeobox, AP2, bZIP_1, MADF_DNA_bdg, Myb_DNA-binding	6	6	31,906	63,811	44,667	19,144
**14**	Homeobox, HLH, SRF-TF, DUF822	6	6	21,856	43,712	30,598	13,114
**15**	SRF-TF	2	2	672	1,344	940	404
	**Total**	310	328	1,241,315	2,482,629	1,737,836	744,793

Table S2. Details of TFBS motifs retrieved from CIS-BP version 1.02 categorized by plant species.

**Plant species**	**Plant species Name**	**Number of TFBS motifs**
Arabidopsis thaliana	Mouse-ear cress	230
Physcomitrella patens	Moss	23
Oryza sativa	Rice	21
Cannabis sativa	Marijuana	15
Arabidopsis lyrata	Mustard	10
Zea mays	Maize	8
Antirrhinum majus	Snapdragon	7
Medicago truncatula	Barrel Clover (legume)	3
Glycine max	Soybean	2
Lotus japonicus	Wild legume	2
Malus x domestica	Domesticated apple	2
Manihot esculenta	Cassava (woody shrub)	2
Vitis vinifera	Common grape vine	2
Brachypodiumdistachyon	Purple false brome (grass)	1
Nicotiana sp.	Tobacco	1
Nicotiana tabacum	Tobacco	1
Petroselinum crispum	Garden parsley	1
Pisum sativum	Pea	1
Populus trichocarpa	Black cottonwood/California Poplar	1
Prunus persica	Peach tree	1
Ricinus communis	Castor oil plant	1
Triticum aestivum	Common wheat	1
**Grand Total**		336

Table S3 Logo plot showing the probability of TFBS-base binding preferences for specific DBD types. The boxplot shows the P(x) of specific TFBS length for each DBD type (the probability of the nucleotide base at each position). Only TFBS base-preferences obtained from more than one experimental data are shown with boxplot. .

TFBS length	DBD type	Number of PWMs	TFBS base-preference pattern	Boxplot of all PWMs used in TFBS base-preference
7	bZIP_1	2	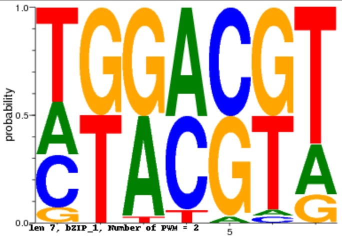	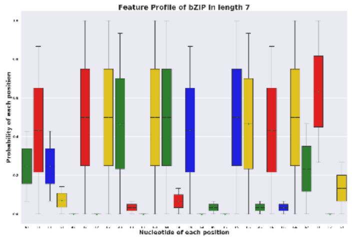
7	GATA	2	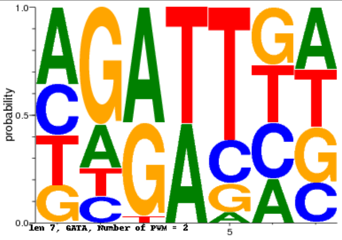	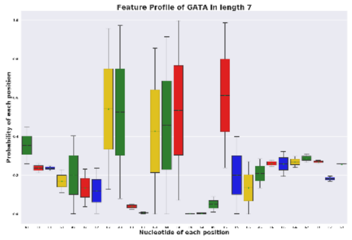
7	HLH	2	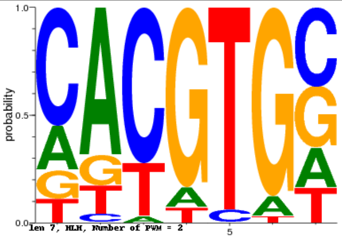	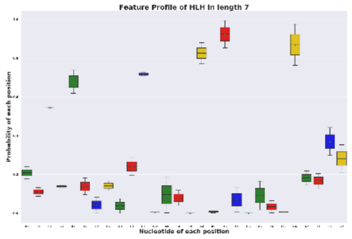
7	HMG_box	1	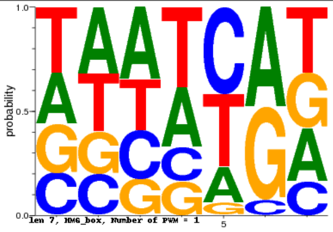	-
7	SBP	1	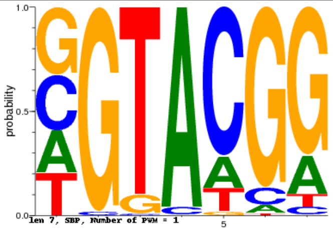	-
7	zf-C2H2	2	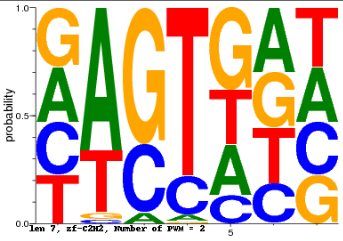	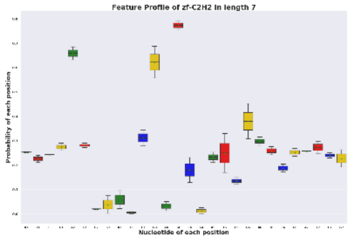

Table S3 (cont.) Logo plot showing the probability of TFBS-base binding preference for specific DBD types. The boxplot shows the P(x) of specific TFBS length for each DBD type (the probability of the nucleotide base at each position). Only TFBS base-preferences obtained from more than one experimental data are shown with boxplot.

TFBS length	DBD type	Number of PWMs	TFBS base-preference pattern	Boxplot of all PWMs used in TFBS base-preference
7	zf-Dof	3	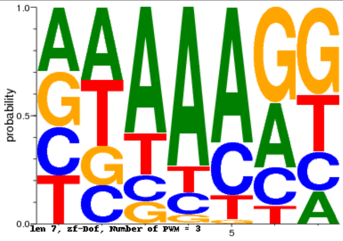	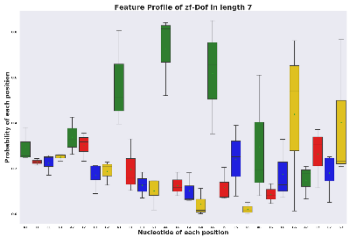
8	AP2	22	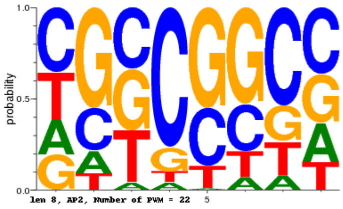	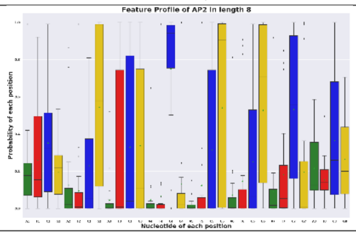
8	AT_hook	1	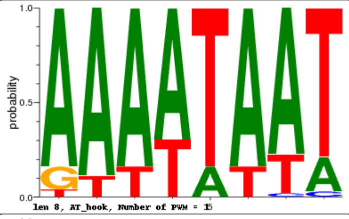	-
8	B3	1	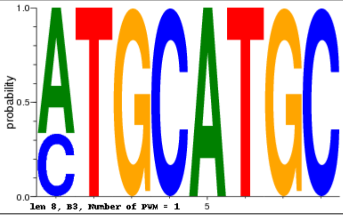	-
8	bZIP_1	5	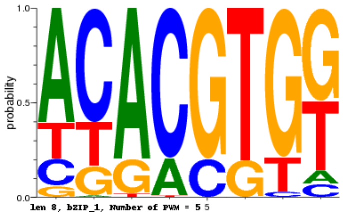	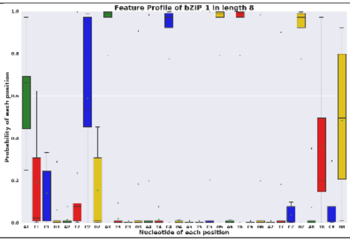
8	CSD	1	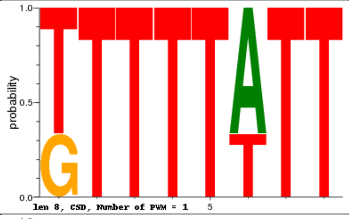	-
8	DUF573	1	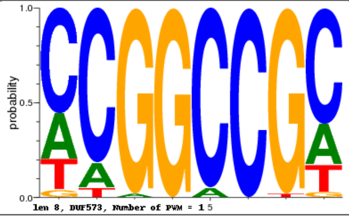	-

Table S3 (cont.) Logo plot showing the probability of TFBS-base binding preference for specific DBD types. The boxplot shows the P(x) of specific TFBS length for each DBD type (the probability of the nucleotide base at each position). Only TFBS base-preferences obtained from more than one experimental data are shown with boxplot.

TFBS length	DBD type	Number of PWMs	TFBS base-preference pattern	Boxplot of all PWMs used in TFBS base-preference
8	EIN3	1	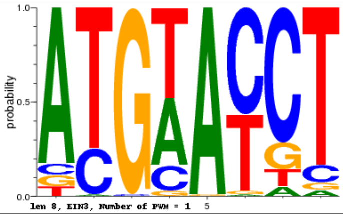	-
8	GATA	2	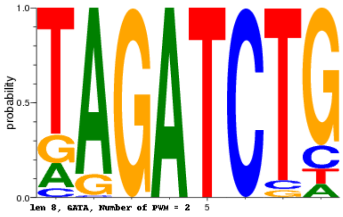	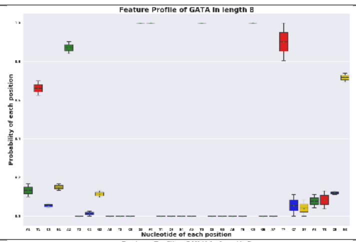
8	HLH	10	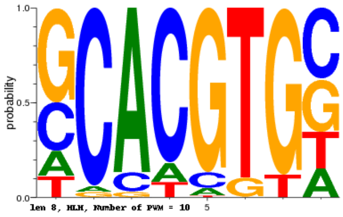	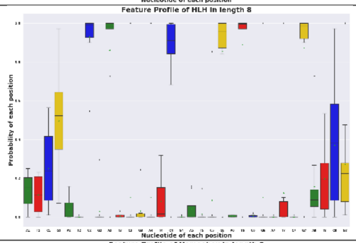
8	Homeobox	9	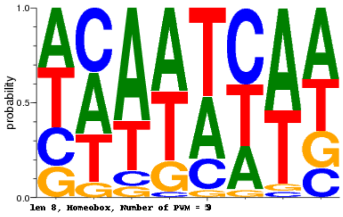	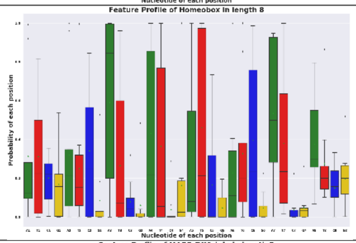
8	MADF_DNA_bdg	4	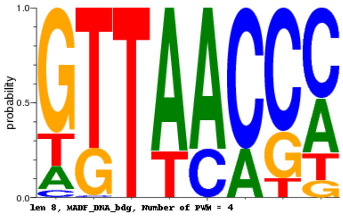	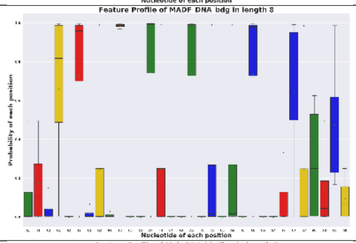
8	Myb_DNA-binding	7	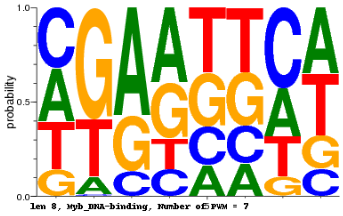	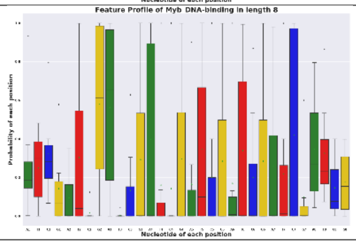

Table S3 (cont.) Logo plot showing the probability of TFBS-base binding preference for specific DBD types. The boxplot shows the P(x) of specific TFBS length for each DBD type (the probability of the nucleotide base at each position). Only TFBS base-preferences obtained from more than one experimental data are shown with boxplot.

TFBS length	DBD type	Number of PWMs	TFBS base-preference pattern	Boxplot of all PWMs used in TFBS base-preference
8	NAM	2	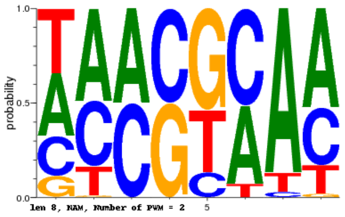	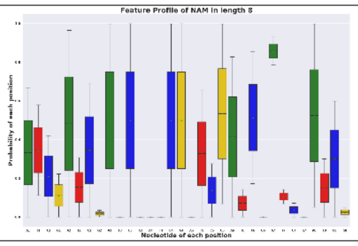
8	SBP	1	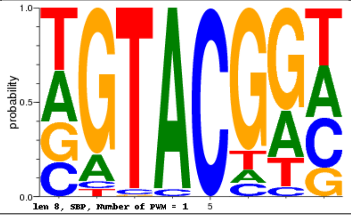	-
8	TCP	9	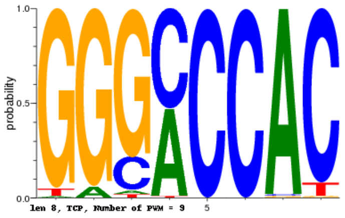	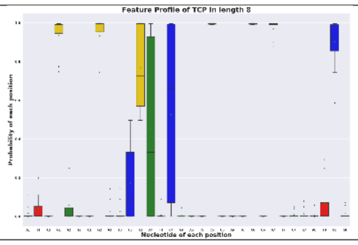
8	WRC	1	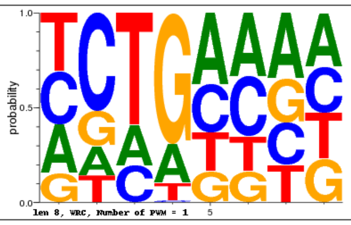	-
8	WRKY	5	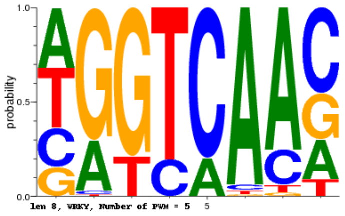	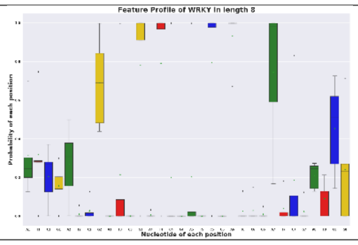
8	zf-C2H2	1	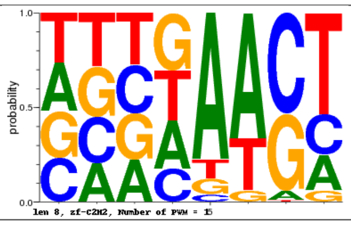	-

Table S3 (cont.) Logo plot showing the probability of TFBS-base binding preference for specific DBD types. The boxplot shows the P(x) of specific TFBS length for each DBD type (the probability of the nucleotide base at each position). Only TFBS base-preferences obtained from more than one experimental data are shown with boxplot.

TFBS length	DBD type	Number of PWMs	TFBS base-preference pattern	Boxplot of all PWMs used in TFBS base-preference
8	zf-Dof	5	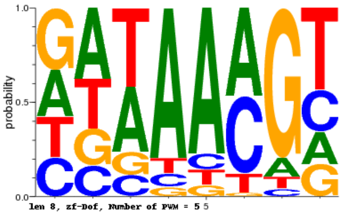	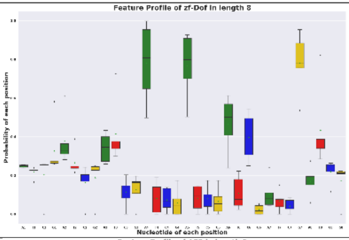
9	AP2	7	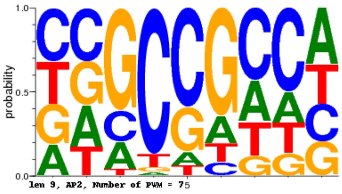	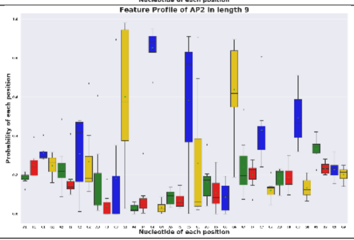
9	AT_hook	1	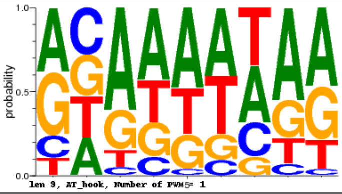	-
9	B3	3	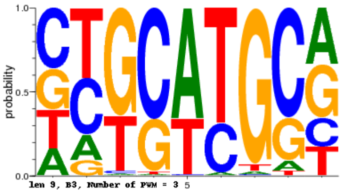	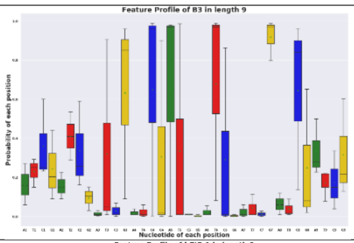
9	bZIP_1	2	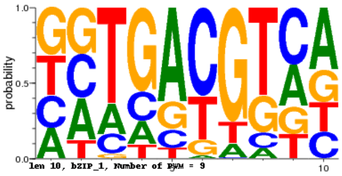	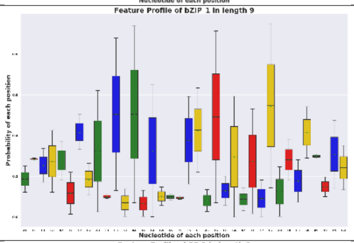
9	CG-1	2	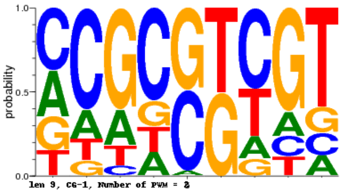	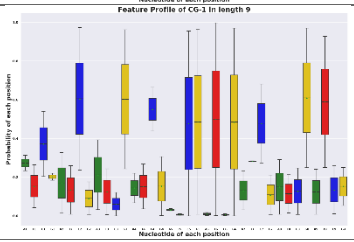
9	DUF573	1	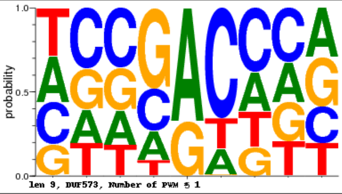	-

Table S3 (cont.) Logo plot showing the probability of TFBS-base binding preference for specific DBD types. The boxplot shows the P(x) of specific TFBS length for each DBD type (the probability of the nucleotide base at each position). Only TFBS base-preferences obtained from more than one experimental data are shown with boxplot.

TFBS length	DBD type	Number of PWMs	TFBS base-preference pattern	Boxplot of all PWMs used in TFBS base-preference
9	EIN3	1	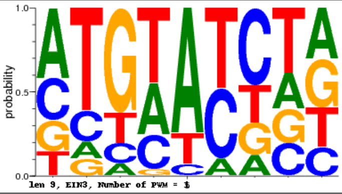	-
9	GATA	4	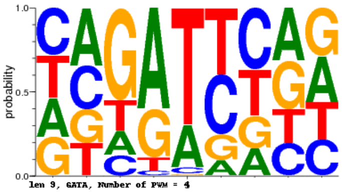	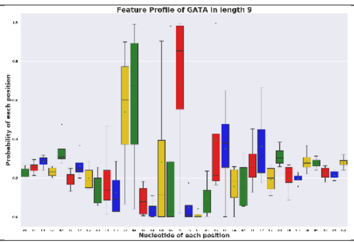
9	GRAS	1	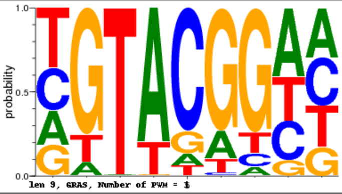	-
9	HLH	3	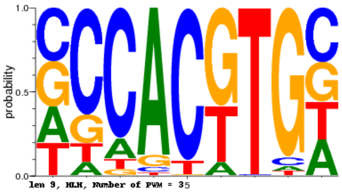	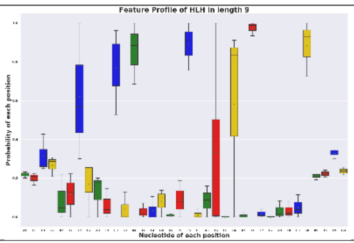
9	HMG_box	1	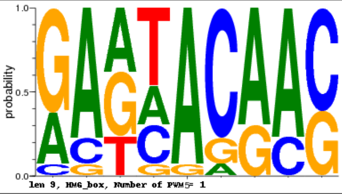	-
9	Homeobox	2	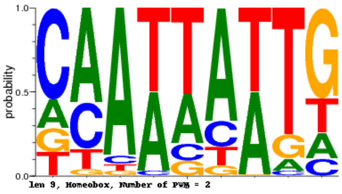	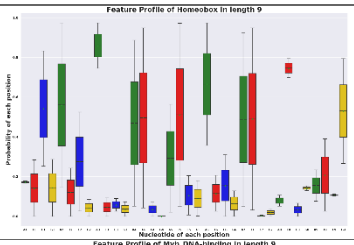
9	Myb_DNA-binding	5	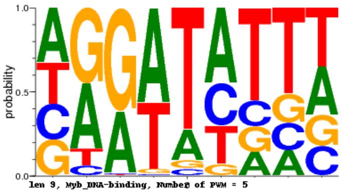	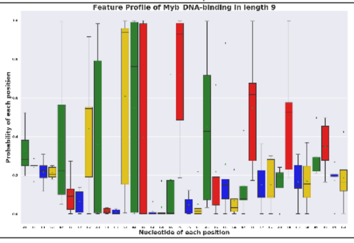

Table S3 (cont.) Logo plot showing the probability of TFBS-base binding preference for specific DBD types. The boxplot shows the P(x) of specific TFBS length for each DBD type (the probability of the nucleotide base at each position). Only TFBS base-preferences obtained from more than one experimental data are shown with boxplot.

TFBS length	DBD type	Number of PWMs	TFBS base-preference pattern	Boxplot of all PWMs used in TFBS base-preference
9	NAM	3	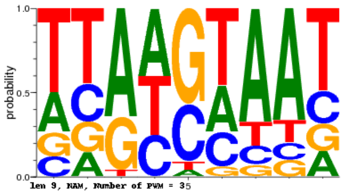	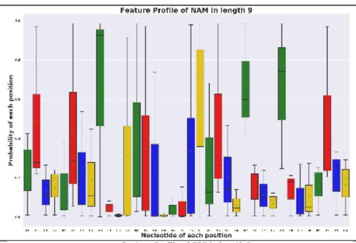
9	SBP	6	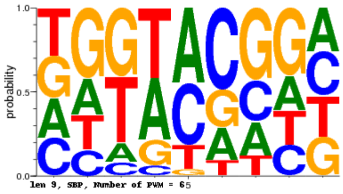	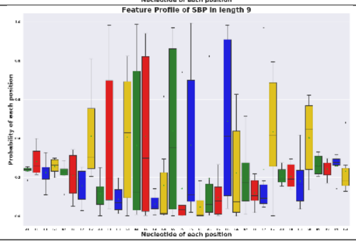
9	TCP	1	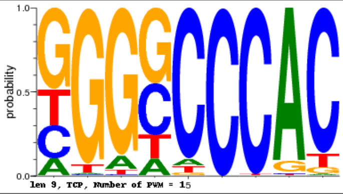	-
9	WRKY	2	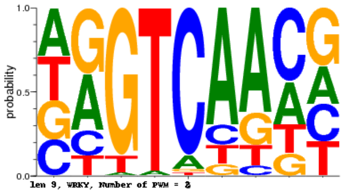	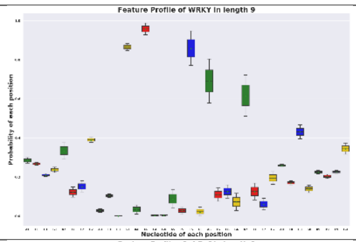
9	zf-Dof	3	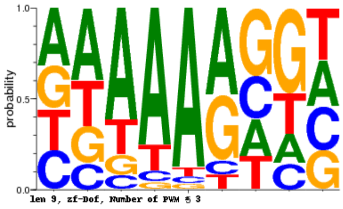	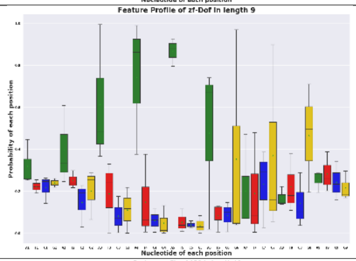
10	AP2	20	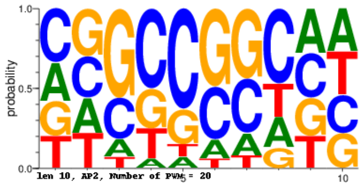	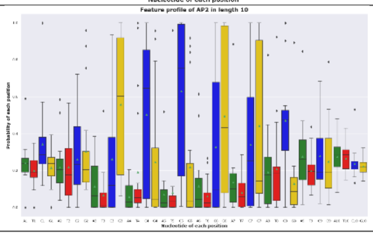
10	B3	1	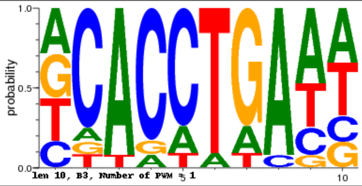	-

Table S3 (cont.) Logo plot showing the probability of TFBS-base binding preference for specific DBD types. The boxplot shows the P(x) of specific TFBS length for each DBD type (the probability of the nucleotide base at each position). Only TFBS base-preferences obtained from more than one experimental data are shown with boxplot.

TFBS length	DBD type	Number of PWMs	TFBS base-preference pattern	Boxplot of all PWMs used in TFBS base-preference
10	bZIP_1	9	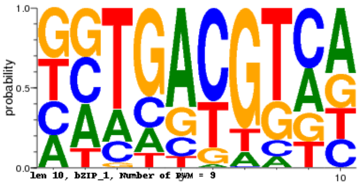	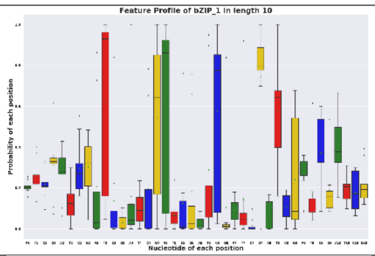
10	DUF260	2	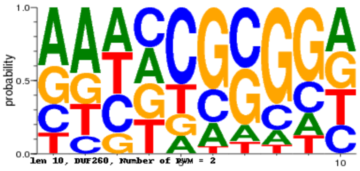	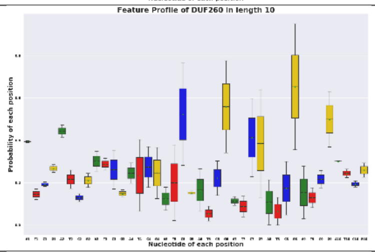
10	DUF573	2	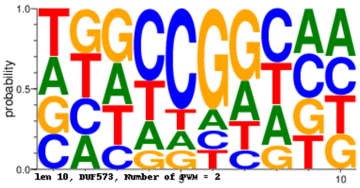	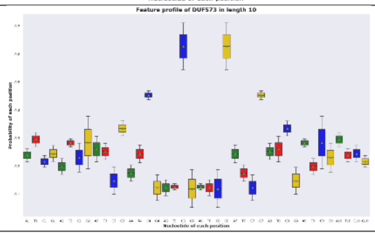
10	E2F_TDP	1	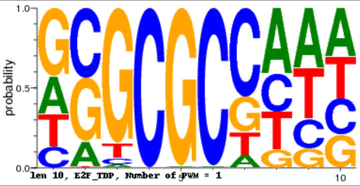	-
10	EIN3	2	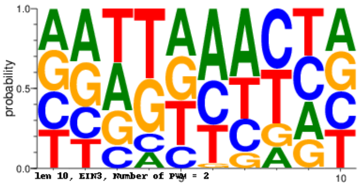	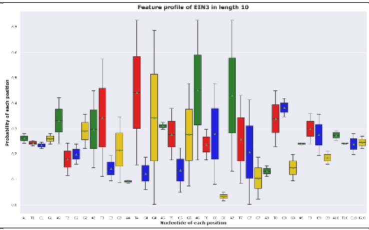
10	GATA	6	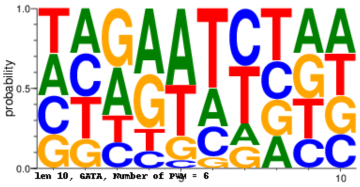	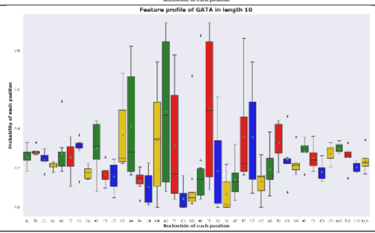

Table S3 (cont.) Logo plot showing the probability of TFBS-base binding preference for specific DBD types. The boxplot shows the P(x) of specific TFBS length for each DBD type (the probability of the nucleotide base at each position). Only TFBS base-preferences obtained from more than one experimental data are shown with boxplot.

TFBS length	DBD type	Number of PWMs	TFBS base-preference pattern	Boxplot of all PWMs used in TFBS base-preference
10	HLH	13	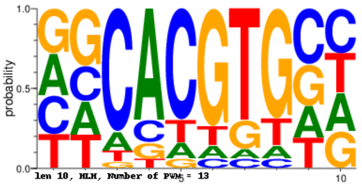	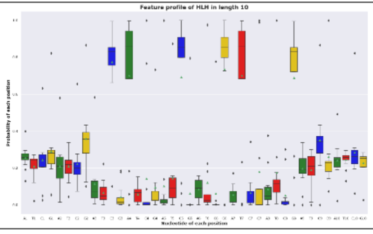
10	HMG_box	1	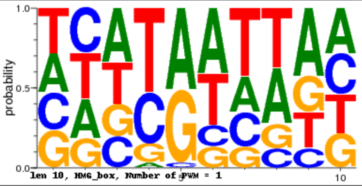	-
10	Homeobox	14	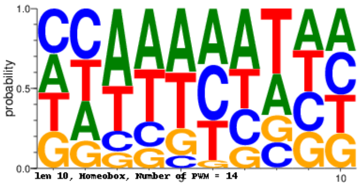	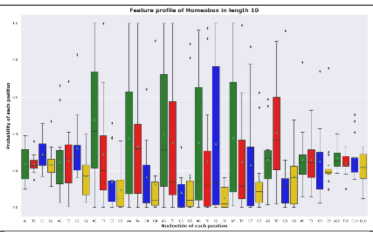
10	MADF_DNA_bdg	8	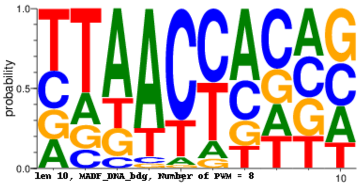	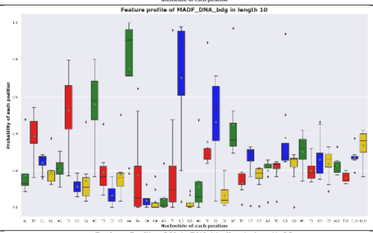
10	Myb_DNA-binding	24	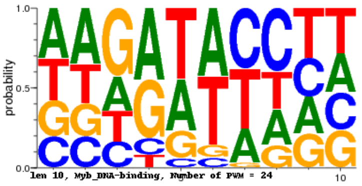	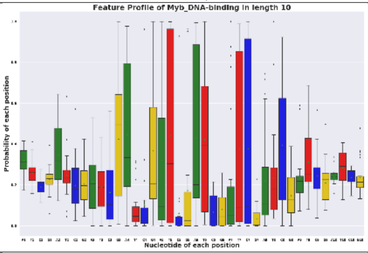
10	NAM	10	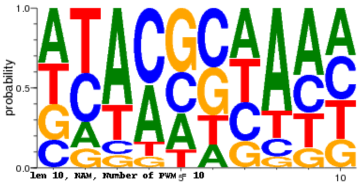	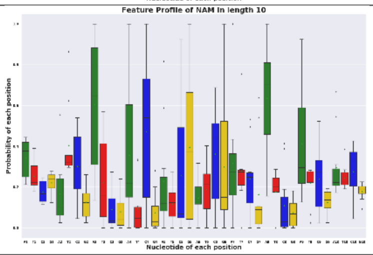

Table S3 (cont.) Logo plot showing the probability of TFBS-base binding preference for specific DBD types. The boxplot shows the P(x) of specific TFBS length for each DBD type (the probability of the nucleotide base at each position). Only TFBS base-preferences obtained from more than one experimental data are shown with boxplot.

TFBS length	DBD type	Number of PWMs	TFBS base-preference pattern	Boxplot of all PWMs used in TFBS base-preference
10	SBP	4	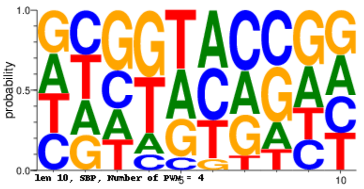	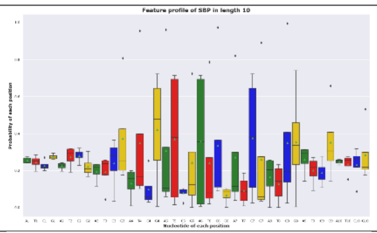
10	TCP	12	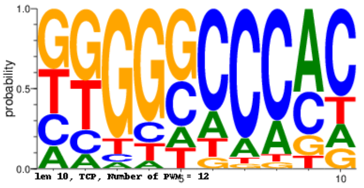	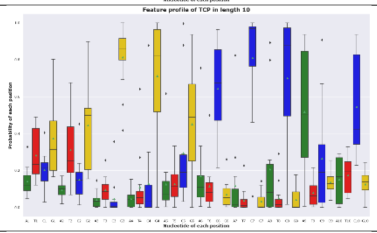
10	WRKY	13	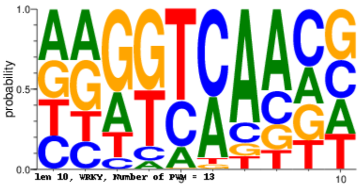	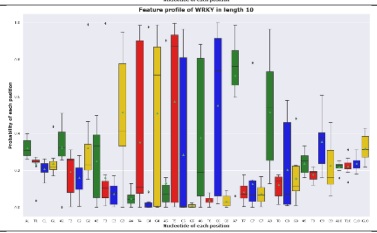
10	zf-Dof	6	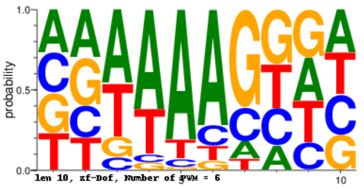	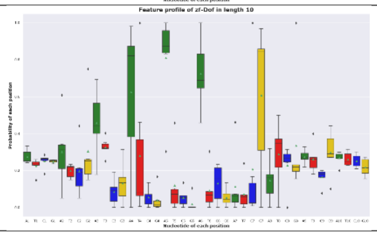
11	B3	1	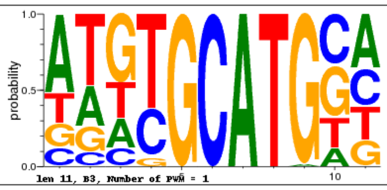	-
11	bZIP_1	3	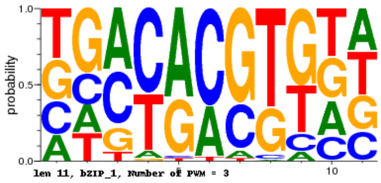	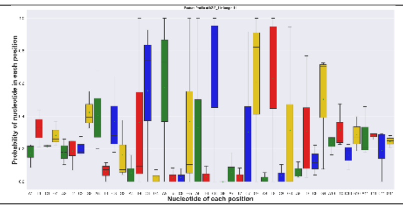
11	DUF822	1	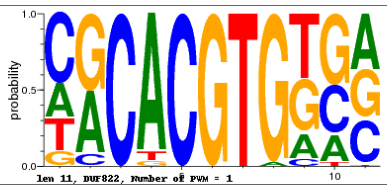	-

Table S3 (cont.) Logo plot showing the probability of TFBS-base binding preference for specific DBD types. The boxplot shows the P(x) of specific TFBS length for each DBD type (the probability of the nucleotide base at each position). Only TFBS base-preferences obtained from more than one experimental data are shown with boxplot.

TFBS length	DBD type	Number of PWMs	TFBS base-preference pattern	Boxplot of all PWMs used in TFBS base-preference
11	EIN3	2	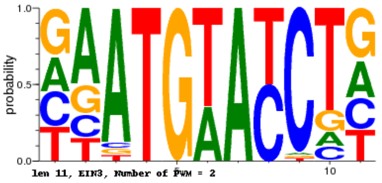	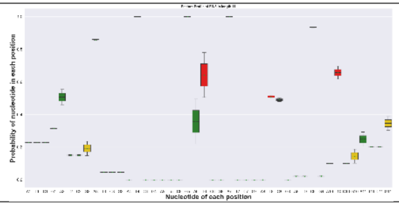
11	GATA	1	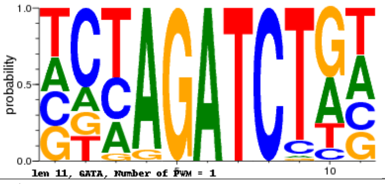	-
11	Homeobox	1	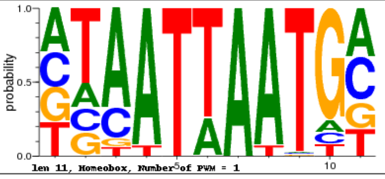	-
11	Myb_DNA-binding	3	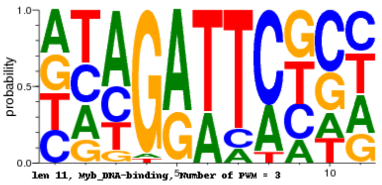	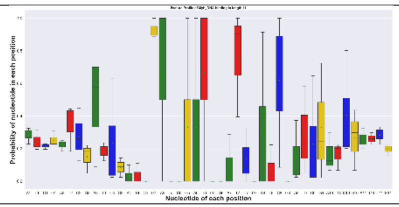
11	NAM	1	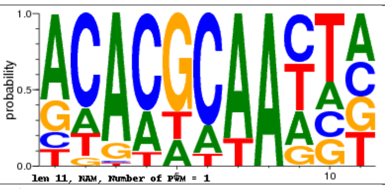	-
11	SRF-TF	1	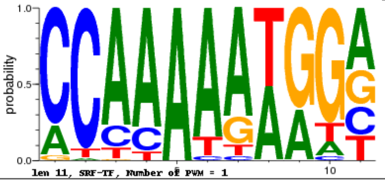	-
11	WRKY	2	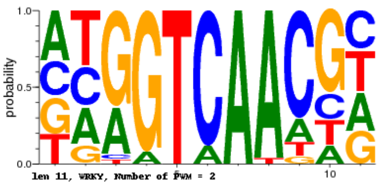	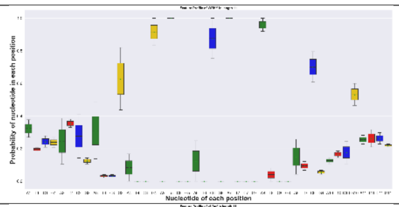
11	zf-Dof	5	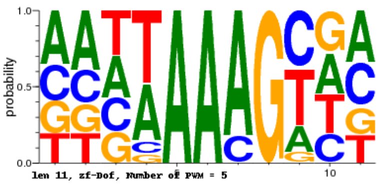	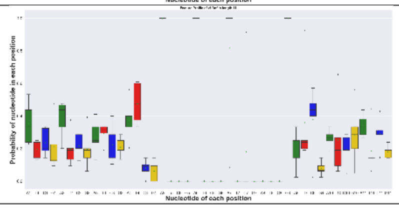

Table S3 (cont.) Logo plot showing the probability of TFBS-base binding preference for specific DBD types. The boxplot shows the P(x) of specific TFBS length for each DBD type (the probability of the nucleotide base at each position). Only TFBS base-preferences obtained from more than one experimental data are shown with boxplot.

TFBS length	DBD type	Number of PWMs	TFBS base-preference pattern	Boxplot of all PWMs used in TFBS base-preference
12	FAR1	1	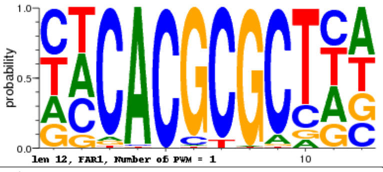	-
12	HLH	1	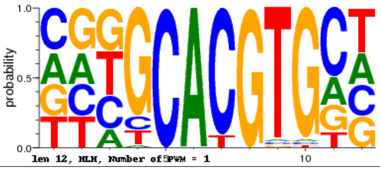	-
12	Homeobox	1	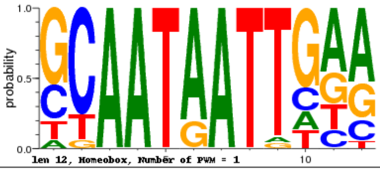	-
12	NAM	1	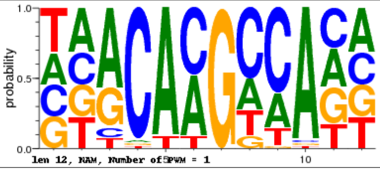	-
12	SBP	1	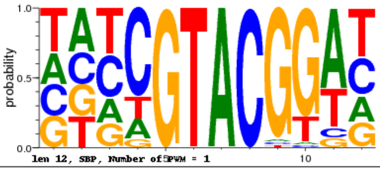	-
12	bZIP_1	6	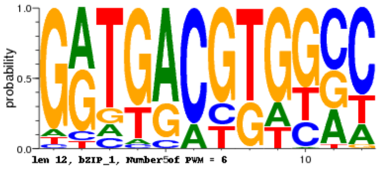	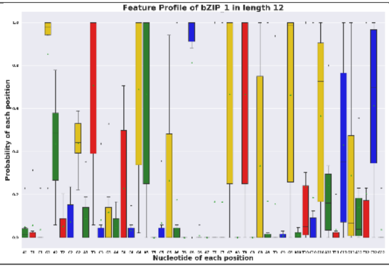
13	AP2	1	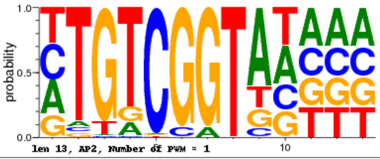	-
13	MADF_DNA_bdg	1	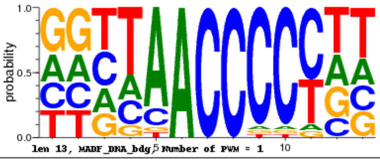	-

Table S3 (cont.) Logo plot showing the probability of TFBS-base binding preference for specific DBD types. The boxplot shows the P(x) of specific TFBS length for each DBD type (the probability of the nucleotide base at each position). Only TFBS base-preferences obtained from more than one experimental data are shown with boxplot.

TFBS length	DBD type	Number of PWMs	TFBS base-preference pattern	Boxplot of all PWMs used in TFBS base-preference
13	Myb_DNA-binding	1	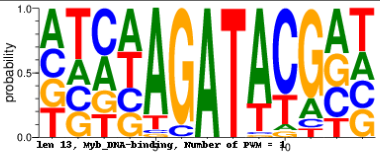	-
13	bZIP_1	1	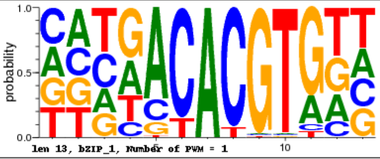	-
13	Homeobox	2	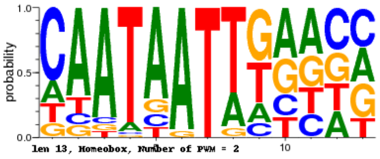	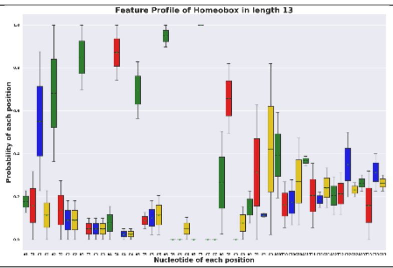
14	DUF822	1	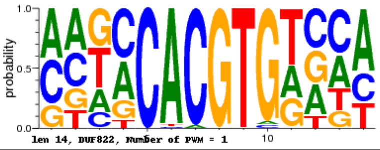	-
14	Homeobox	1	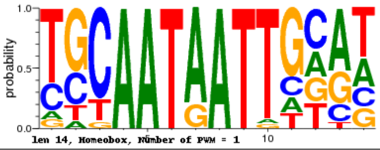	-
14	HLH	2	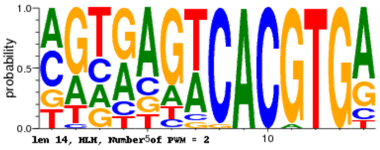	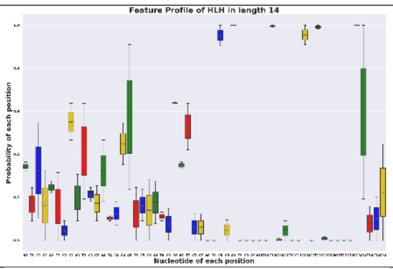
14	SRF-TF	2	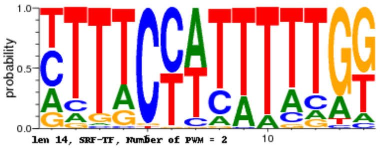	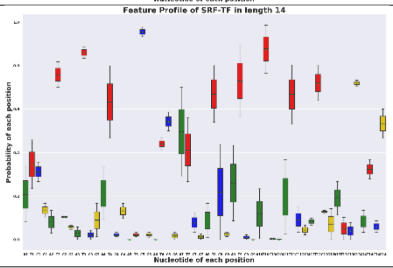

Table S3 (cont.) Logo plot showing the probability of TFBS-base binding preference for specific DBD types. The boxplot shows the P(x) of specific TFBS length for each DBD type (the probability of the nucleotide base at each position). Only TFBS base-preferences obtained from more than one experimental data are shown with boxplot.

TFBS length	DBD type	Number of PWMs	TFBS base-preference pattern	Boxplot of all PWMs used in TFBS base-preference
15	SRF-TF	2	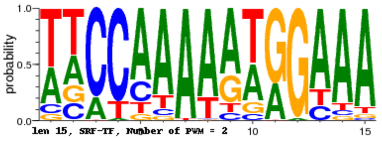	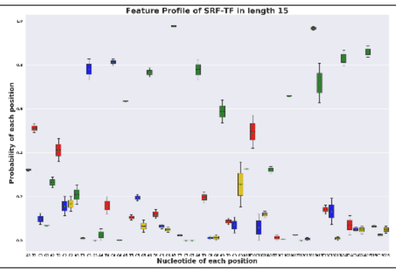

Table S4 Hyperparameter tuning of RF and k-NN models trained using TFBS information from the binary and TFBS base-preference representations to optimize the number of trees and neighbors. (Some model results do not show a trend line because the performance is very close to 1.)

TFBS length	Negative data generation	Classifier	Binary	TFBS base-preference
7	RW	RF	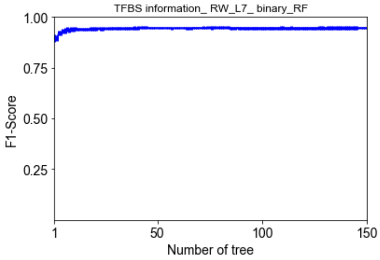	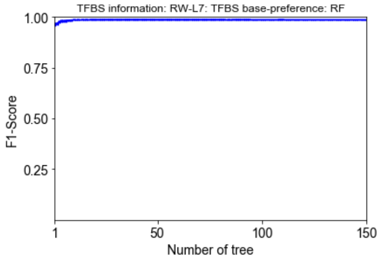
8	RW	RF	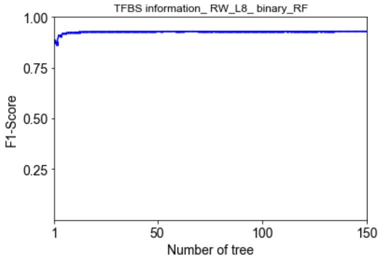	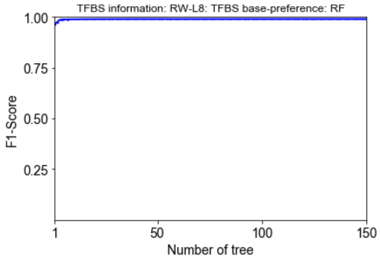
9	RW	RF	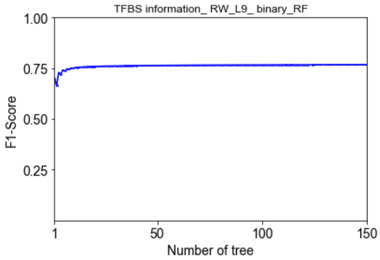	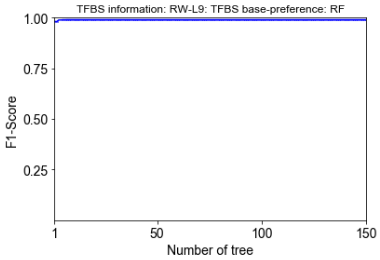
10	RW	RF	N/A	N/A
11	RW	RF	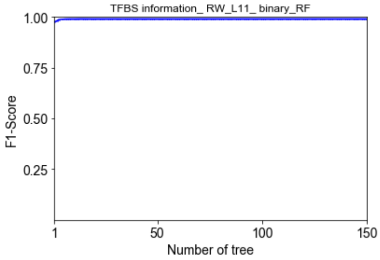	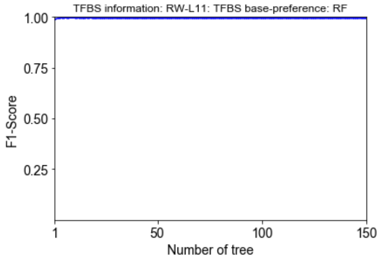
12	RW	RF	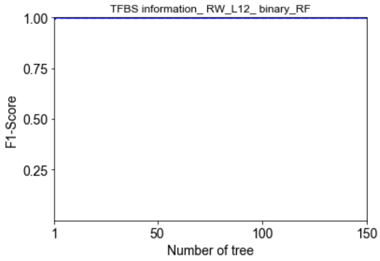	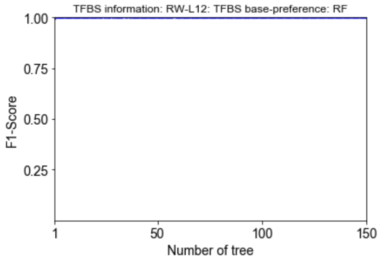

Table S4 (cont.) Hyperparameter tuning of RF and k-NN models trained using TFBS information from the binary and TFBS base-preference representations to optimize the number of trees and neighbors. (Some model results do not show a trend line because the performance is very close to 1.)

TFBS length	Negative data generation	Classifier	Binary	TFBS base-preference
13	RW	RF	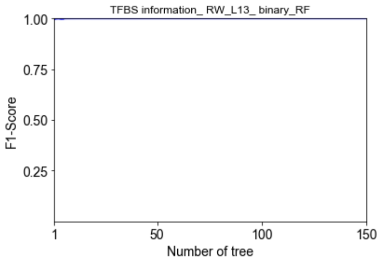	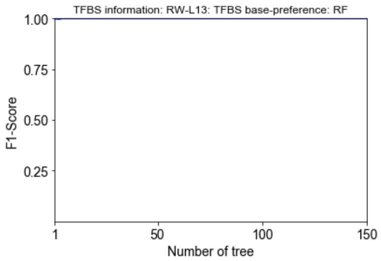
14	RW	RF	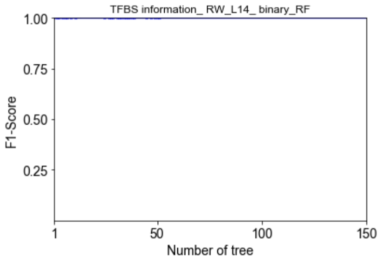	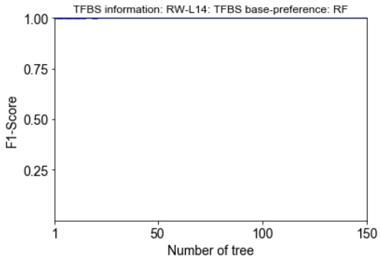
15	RW	RF	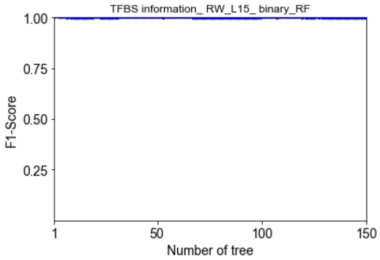	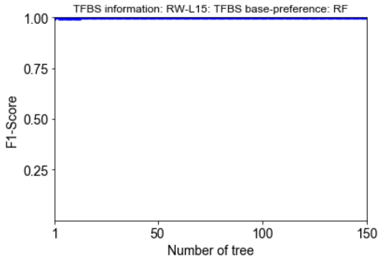
7	RP	RF	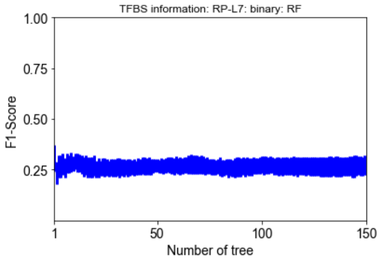	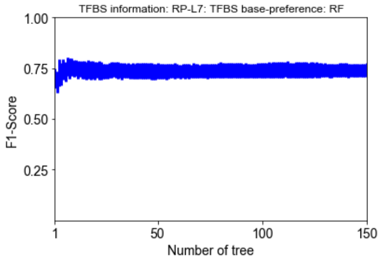
8	RP	RF	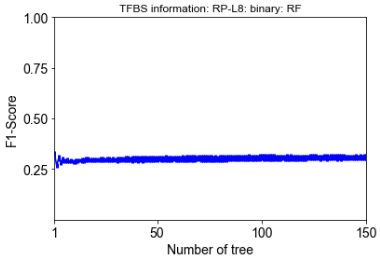	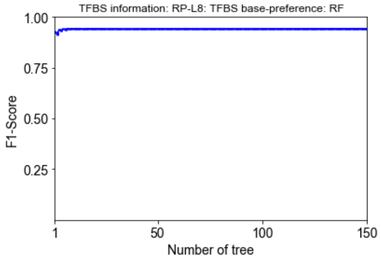
9	RP	RF	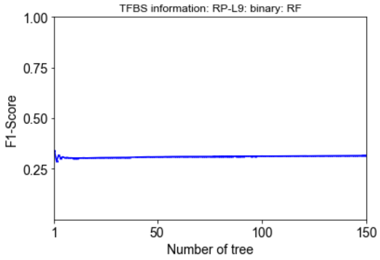	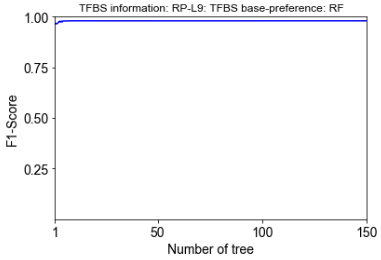

Table S4 (cont.) Hyperparameter tuning of RF and k-NN models trained using TFBS information from the binary and TFBS base-preference representations to optimize the number of trees and neighbors. (Some model results do not show a trend line because the performance is very close to 1.)

TFBS length	Negative data generation	Classifier	Binary	TFBS base-preference
10	RP	RF	N/A	N/A
11	RP	RF	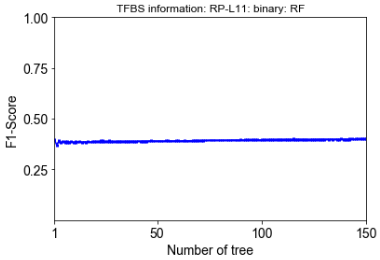	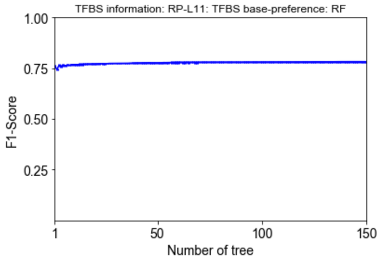
12	RP	RF	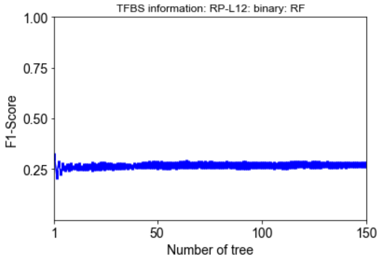	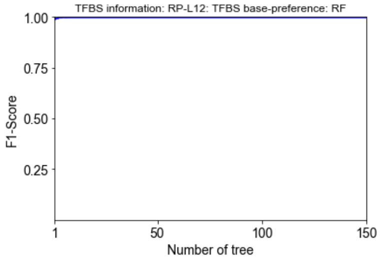
13	RP	RF	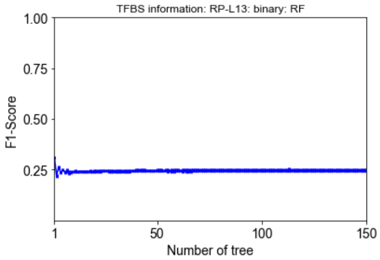	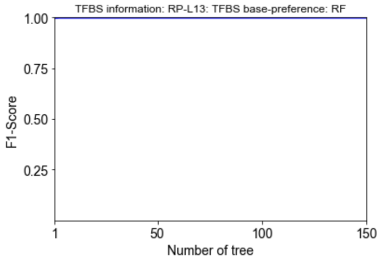
14	RP	RF	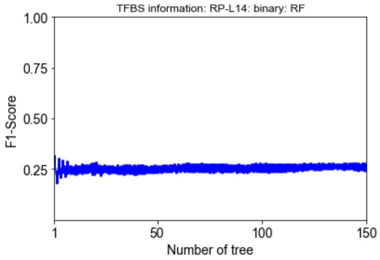	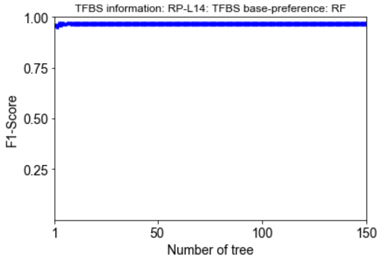
7	RW	k-NN	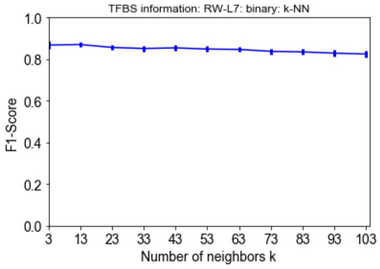<br>*Selected number of neighbors = 13	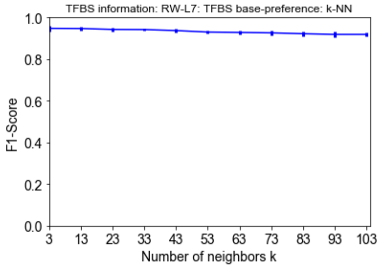<br>*Selected number of neighbors = 3

Table S4 (cont.) Hyperparameter tuning of RF and k-NN models trained using TFBS information from the binary and TFBS base-preference representations to optimize the number of trees and neighbors. (Some model results do not show a trend line because the performance is very close to 1.)

TFBS length	Negative data generation	Classifier	Binary	TFBS base-preference
8	RW	k-NN	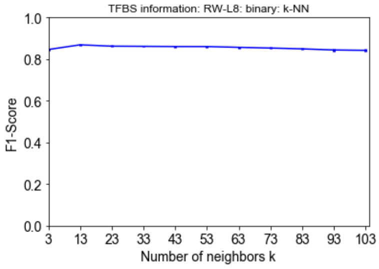<br>*Selected number of neighbors = 13	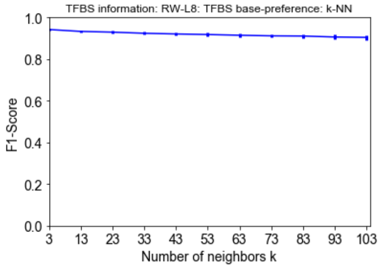<br>*Selected number of neighbors = 3
9	RW	k-NN	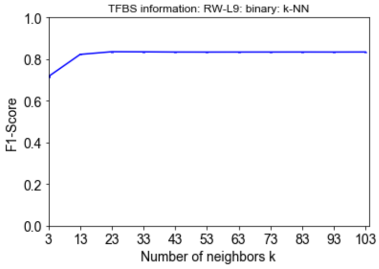<br>*Selected number of neighbors = 13	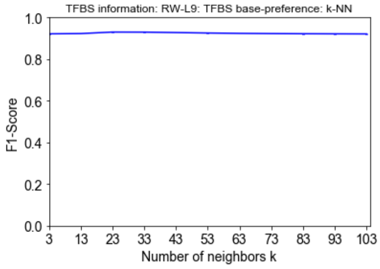<br>*Selected number of neighbors = 23
10	RW	k-NN	N/A	N/A
11	RW	k-NN	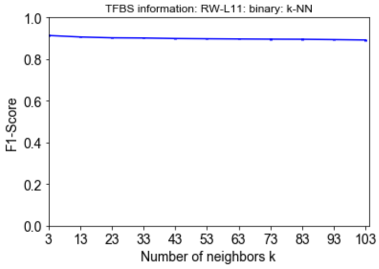<br>*Selected number of neighbors = 3	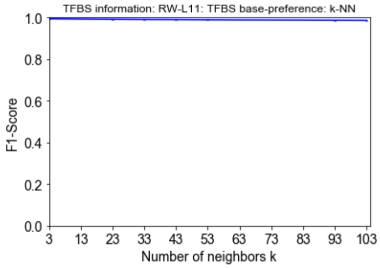<br>*Selected number of neighbors = 3
12	RW	k-NN	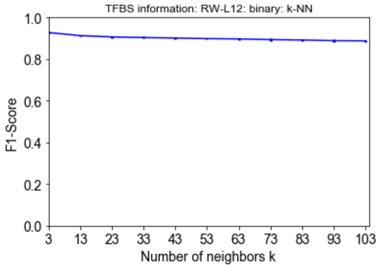<br>*Selected number of neighbors = 3	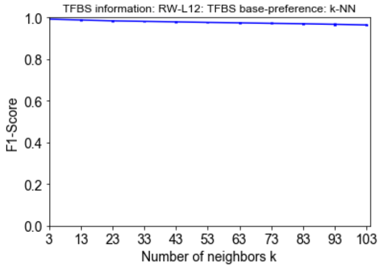<br>*Selected number of neighbors = 3
13	RW	k-NN	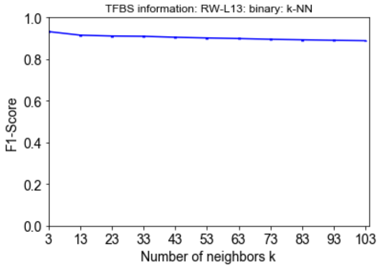<br>*Selected number of neighbors = 3	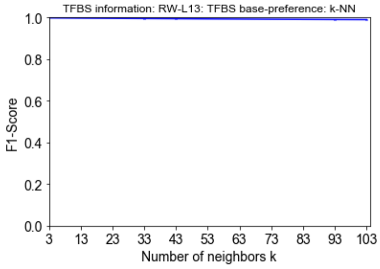<br>*Selected number of neighbors = 3

Table S4 (cont.) Hyperparameter tuning of RF and k-NN models trained using TFBS information from the binary and TFBS base-preference representations to optimize the number of trees and neighbors. (Some model results do not show a trend line because the performance is very close to 1.)

TFBS length	Negative data generation	Classifier	Binary	TFBS base-preference
14	RW	k-NN	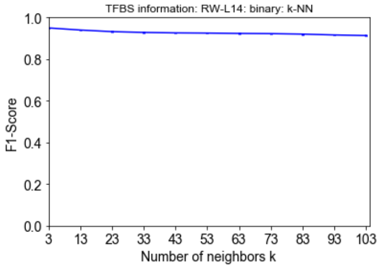<br>*Selected number of neighbors = 3	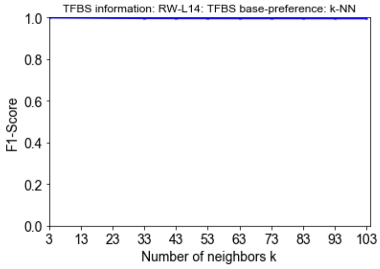<br>*Selected number of neighbors = 3
15	RW	k-NN	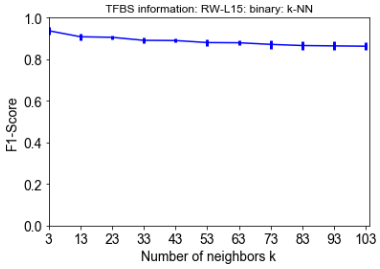<br>*Selected number of neighbors = 3	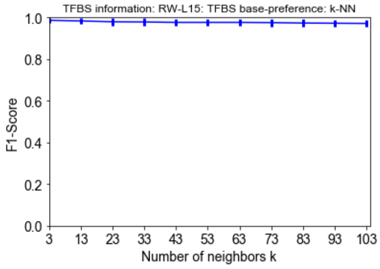<br>*Selected number of neighbors = 3
7	RP	k-NN	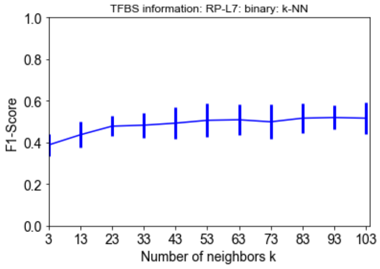<br>*Selected number of neighbors = 93	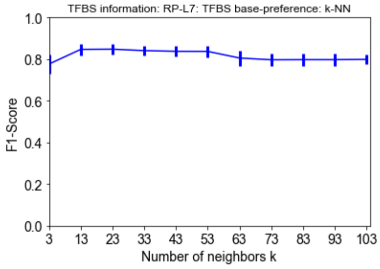<br>*Selected number of neighbors = 23
8	RP	k-NN	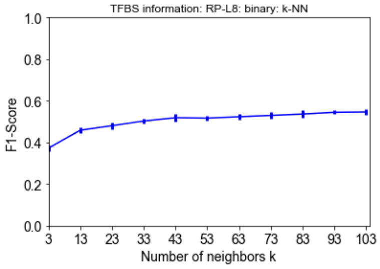<br>*Selected number of neighbors = 103	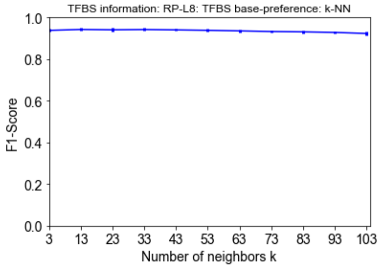<br>*Selected number of neighbors = 13
9	RP	k-NN	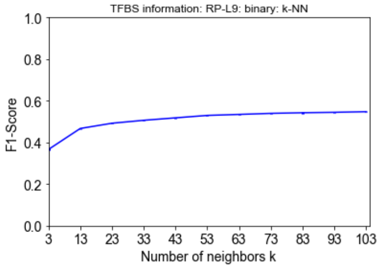<br>*Selected number of neighbors = 103	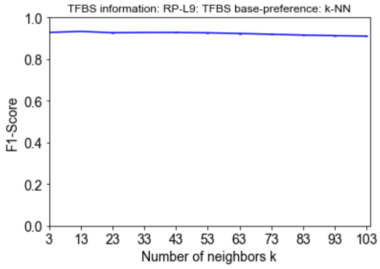<br>*Selected number of neighbors = 13
10	RP	k-NN	N/A	N/A

Table S4 (cont.) Hyperparameter tuning of RF and k-NN models trained using TFBS information from the binary and TFBS base-preference representations to optimize the number of trees and neighbors. (Some model results do not show a trend line because the performance is very close to 1.)

TFBS length	Negative data generation	Classifier	Binary	TFBS base-preference
11	RP	k-NN	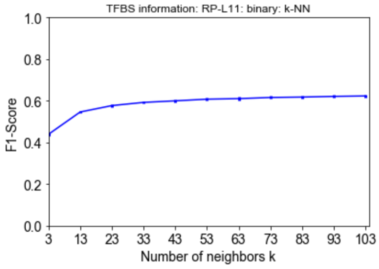<br>*Selected number of neighbors = 103	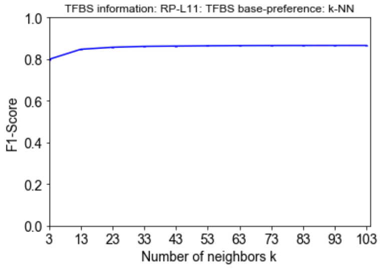<br>*Selected number of neighbors = 103
12	RP	k-NN	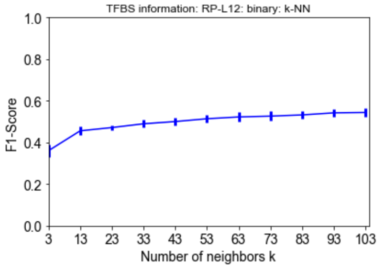<br>*Selected number of neighbors = 103	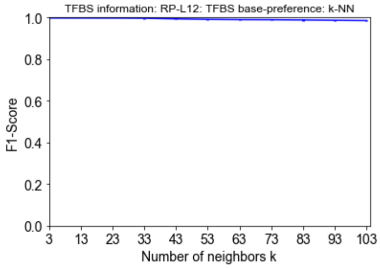<br>*Selected number of neighbors = 3
13	RP	k-NN	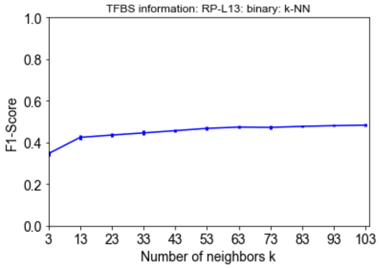<br>*Selected number of neighbors = 103	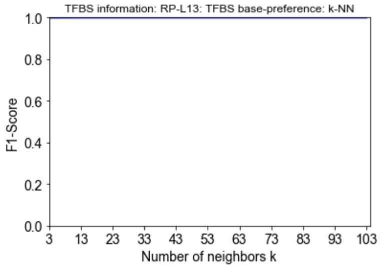<br>*Selected number of neighbors = 23
14	RP	k-NN	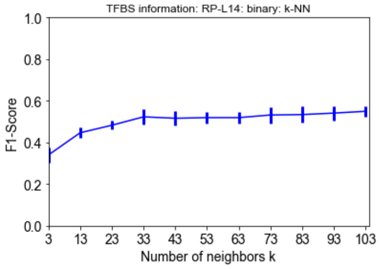<br>*Selected number of neighbors = 103	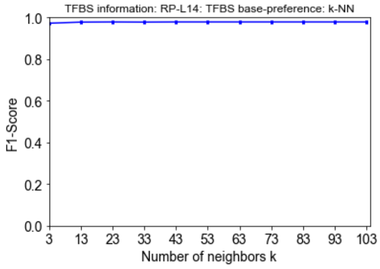<br>*Selected number of neighbors = 43

Table S5 Hyperparameter tuning of RF and k-NN models trained using DBD and TFBS information from the binary and TFBS base-preference representations to optimize the number of trees and neighbors. (Some model results do not show a trend line because the performance is very close to 1.)

TFBS length	Negative data generation	Classifier	DBD + Binary	DBD + TFBS base-preference
7	RW	RF	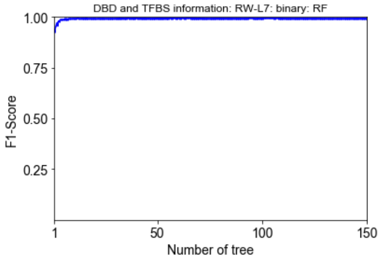	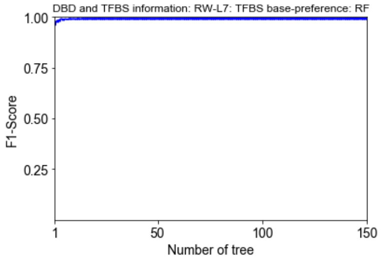
8	RW	RF	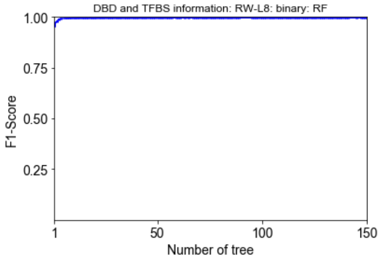	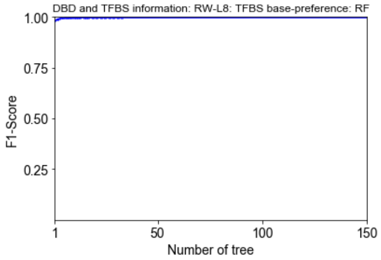
9	RW	RF	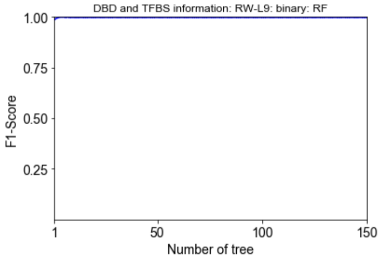	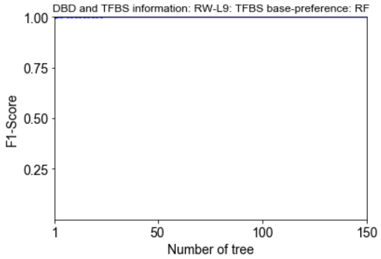
10	RW	RF	N/A	N/A
11	RW	RF	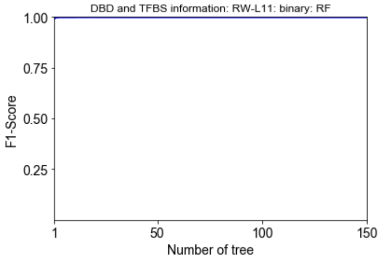	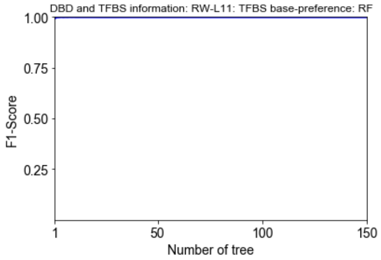
12	RW	RF	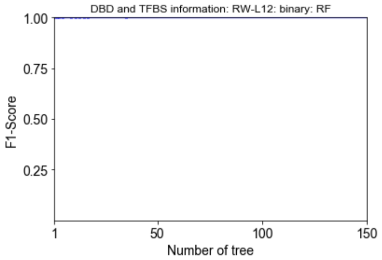	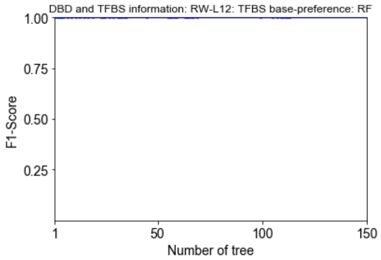

Table S5 (cont.) Hyperparameter tuning of RF and k-NN models trained using DBD and TFBS information from the binary and TFBS base-preference representations to optimize the number of trees and neighbors. (Some model results do not show a trend line because the performance is very close to 1.)

<table>
<tr><th>TFBS length</th><th>Negative data generation</th><th>Classifier</th><th>DBD + Binary</th><th>DBD + TFBS base-preference</th></tr>
<tr><td>13</td><td>RW</td><td>RF</td><td>
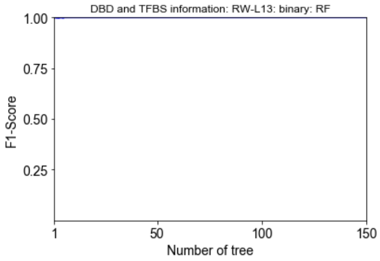

</td><td>
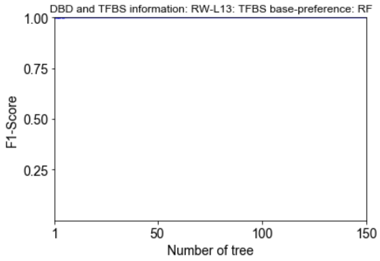

</td></tr>
<tr><td>14</td><td>RW</td><td>RF</td><td>
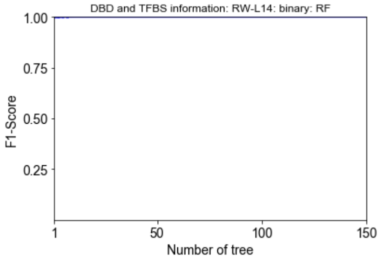

</td><td>
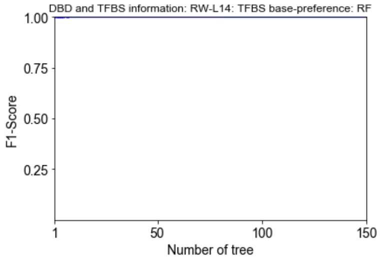

</td></tr>
<tr><td>15</td><td>RW</td><td>RF</td><td>
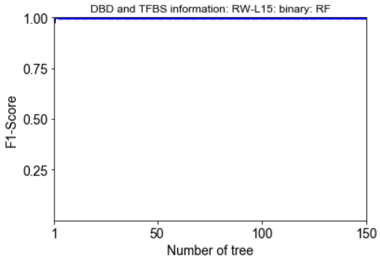

</td><td>
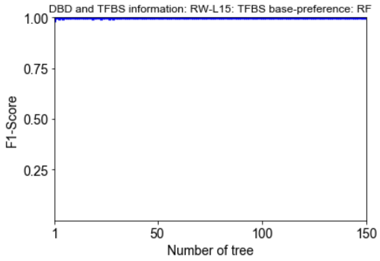

</td></tr>
<tr><td>7</td><td>RP</td><td>RF</td><td>
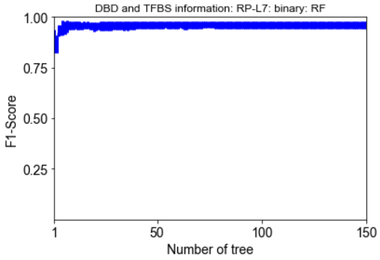

</td><td>
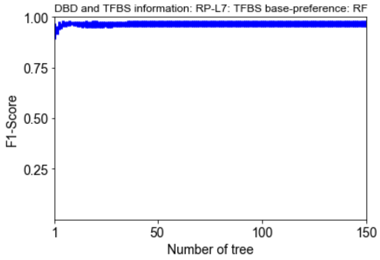

</td></tr>
<tr><td>8</td><td>RP</td><td>RF</td><td>
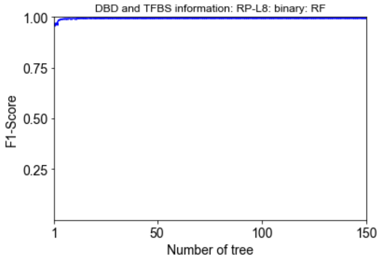

</td><td>
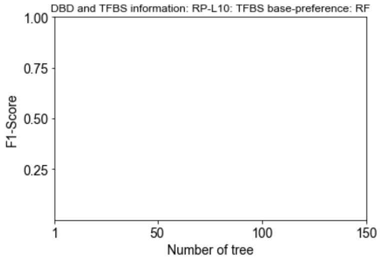

</td></tr>
<tr><td>9</td><td>RP</td><td>RF</td><td>
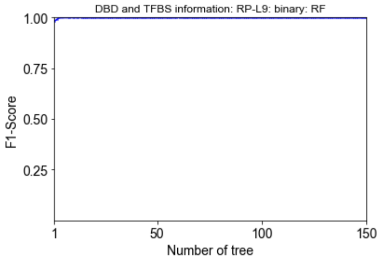

</td><td>
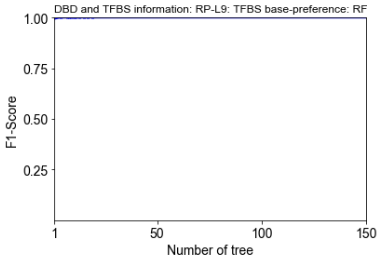

</td></tr>
</table>

Table S5 (cont.) Hyperparameter tuning of RF and k-NN models trained using DBD and TFBS information from the binary and TFBS base-preference representations to optimize the number of trees and neighbors. (Some model results do not show a trend line because the performance is very close to 1.)

TFBS length	Negative data generation	Classifier	DBD + Binary	DBD + TFBS base-preference
10	RP	RF	N/A	N/A
11	RP	RF	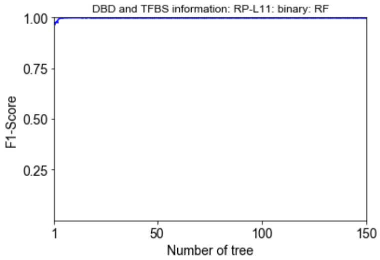	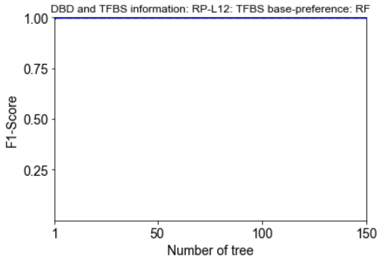
12	RP	RF	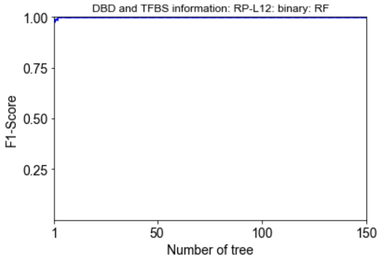	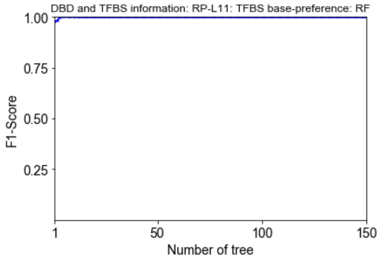
13	RP	RF	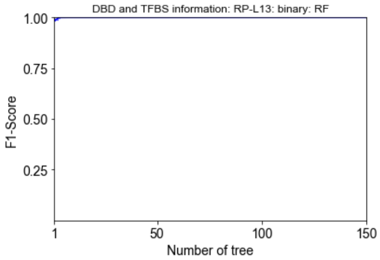	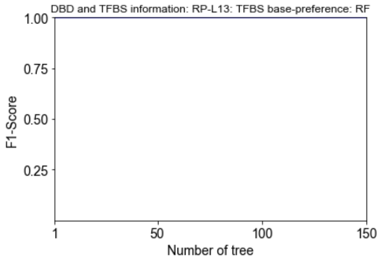
14	RP	RF	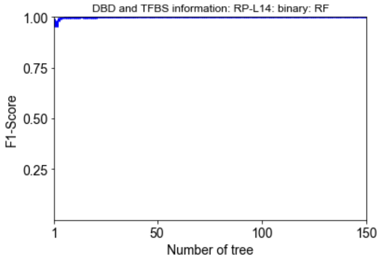	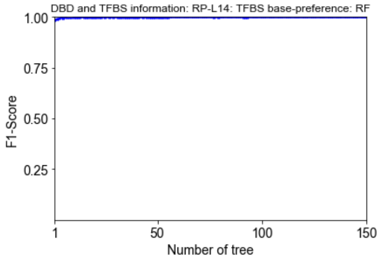
7	RW	k-NN	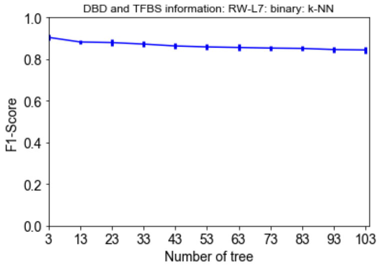 *Selected number of neighbors = 3	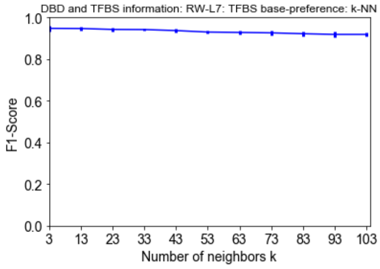 *Selected number of neighbors = 3

Table S5 (cont.) Hyperparameter tuning of RF and k-NN models trained using DBD and TFBS information from the binary and TFBS base-preference representations to optimize the number of trees and neighbors. (Some model results do not show a trend line because the performance is very close to 1.)

TFBS length	Negative data generation	Classifier	DBD + Binary	DBD + TFBS base-preference
8	RW	k-NN	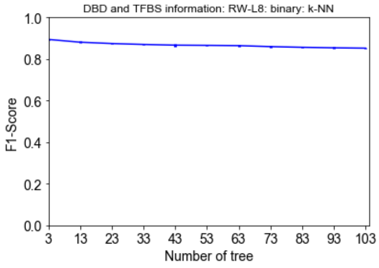<br>*Selected number of neighbors = 3	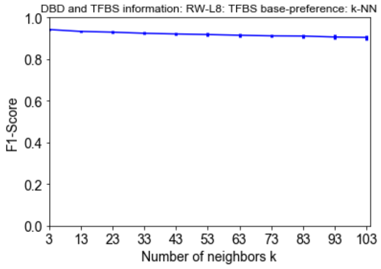<br>*Selected number of neighbors = 3
9	RW	k-NN	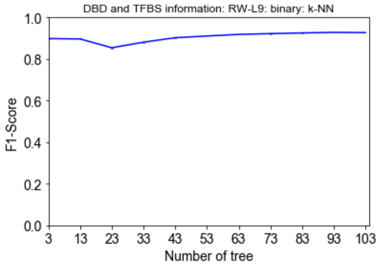<br>*Selected number of neighbors = 93	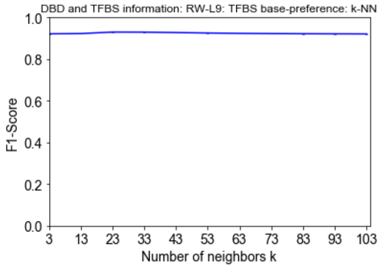<br>*Selected number of neighbors = 23
10	RW	k-NN	N/A	N/A
11	RW	k-NN	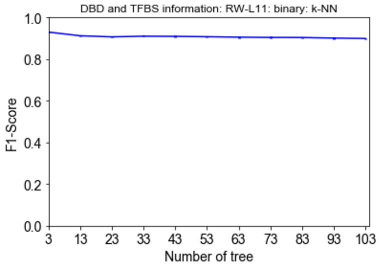<br>*Selected number of neighbors = 3	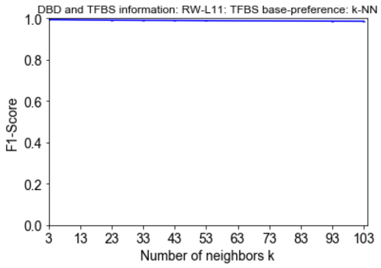<br>*Selected number of neighbors = 3
12	RW	k-NN	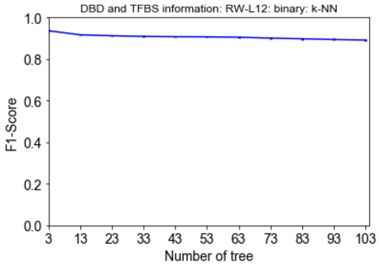<br>*Selected number of neighbors = 3	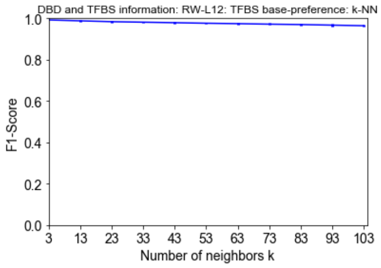<br>*Selected number of neighbors = 3
13	RW	k-NN	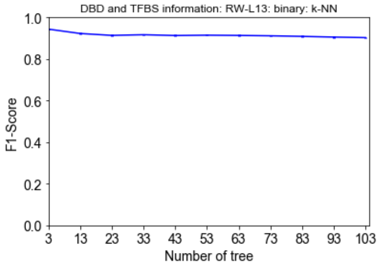<br>*Selected number of neighbors = 3	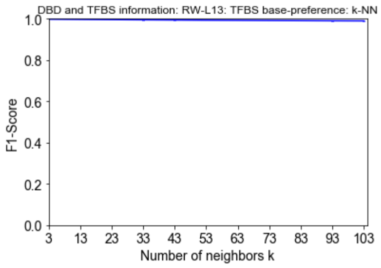<br>*Selected number of neighbors = 3

Table S5 (cont.) Hyperparameter tuning of RF and k-NN models trained using DBD and TFBS information from the binary and TFBS base-preference representations to optimize the number of trees and neighbors. (Some model results do not show a trend line because the performance is very close to 1.)

TFBS length	Negative data generation	Classifier	DBD + Binary	DBD + TFBS base-preference
14	RW	k-NN	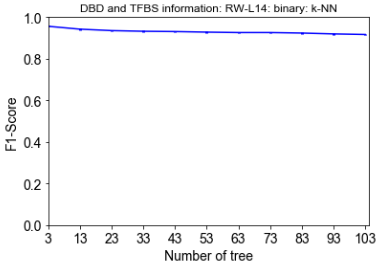<br>*Selected number of neighbors = 3	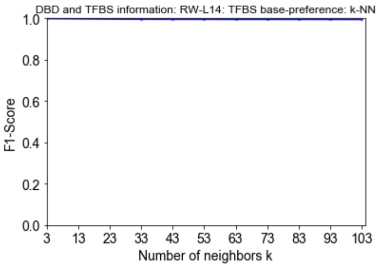<br>*Selected number of neighbors = 3
15	RW	k-NN	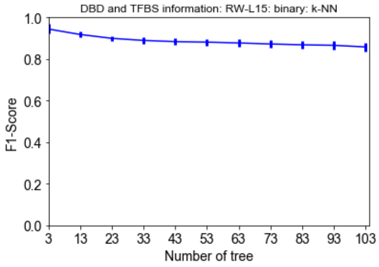<br>*Selected number of neighbors = 3	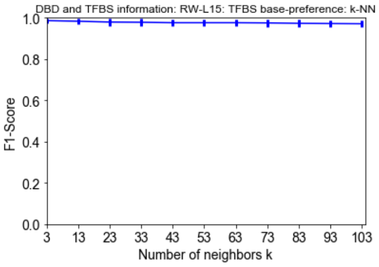<br>*Selected number of neighbors = 3
7	RP	k-NN	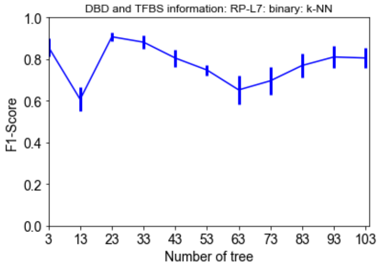<br>*Selected number of neighbors = 23	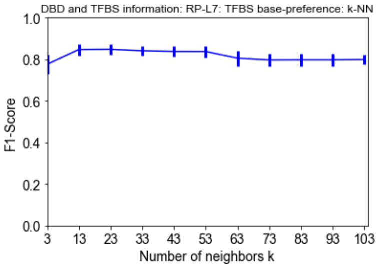<br>*Selected number of neighbors = 13
8	RP	k-NN	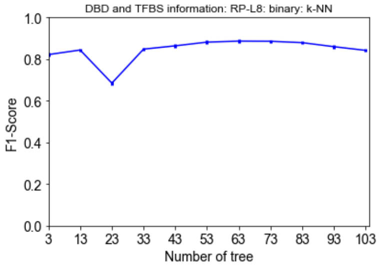<br>*Selected number of neighbors = 63	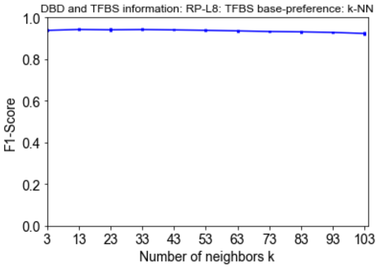<br>*Selected number of neighbors = 13
9	RP	k-NN	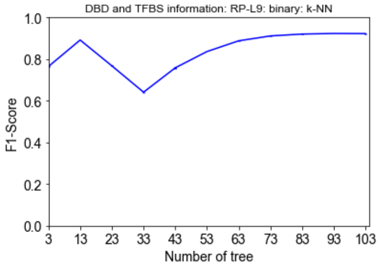<br>*Selected number of neighbors = 93	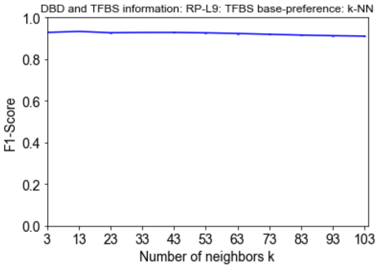<br>*Selected number of neighbors = 43
10	RP	k-NN	N/A	N/A

Table S5 (cont.) Hyperparameter tuning of RF and k-NN models trained using DBD and TFBS information from the binary and TFBS base-preference representations to optimize the number of trees and neighbors. (Some model results do not show a trend line because the performance is very close to 1.)

TFBS length	Negative data generation	Classifier	DBD + Binary	DBD + TFBS base-preference
11	RP	k-NN	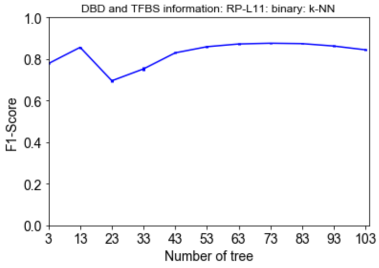<br>*Selected number of neighbors = 73	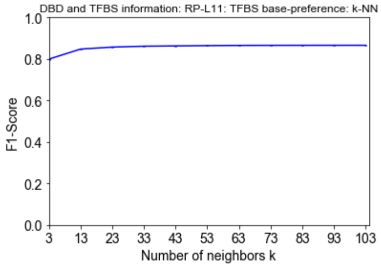<br>*Selected number of neighbors = 43
12	RP	k-NN	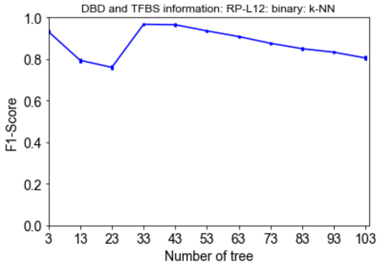<br>*Selected number of neighbors = 33	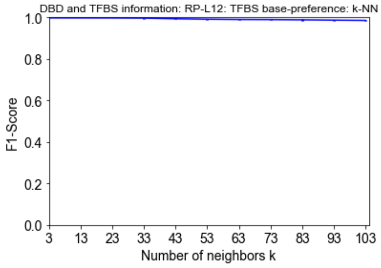<br>*Selected number of neighbors = 3
13	RP	k-NN	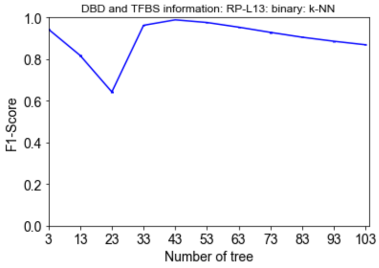<br>*Selected number of neighbors = 43	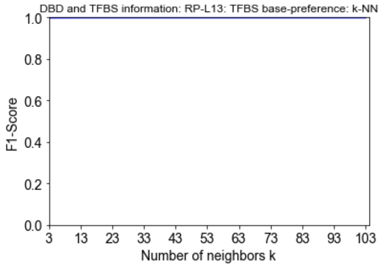<br>*Selected number of neighbors = 13
14	RP	k-NN	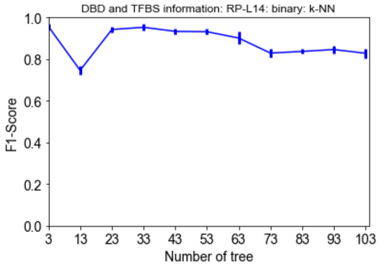<br>*Selected number of neighbors = 3	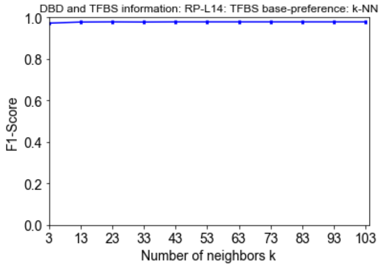<br>*Selected number of neighbors = 43

Table S6 Performance of Plant-DTI model on the 30 percent hold-out test set.

Plant-DTI model							
**TFBS-length Models**	**Accuracy**	**Precision**	**Recall (Sensitivity)**	**Specificity**	**F1-score**	**False Positive Rate (FPR)**	**AUROC**
**Random pairs (RP)**							
**7**	0.9667	0.955	0.9795	0.9538	0.9671	0.0462	0.9921
**8**	0.9946	0.9921	0.9972	0.992	0.9946	0.0080	0.9999
**9**	0.9995	0.9993	0.9998	0.9993	0.9995	0.0007	1
**10**	0.9982	0.9978	0.9986	0.9978	0.9982	0.0023	0.9994
**11**	0.9997	0.9994	1	0.9994	0.9997	0.0011	1
**12**	1	1	1	1	1	0	1
**13**	1	1	1	1	1	0	1
**14**	1	1	1	1	1	0	1
**15**	N/A	N/A	N/A	N/A	N/A	N/A	N/A
**Random within (RW)**							
**7**	0.9932	0.9918	0.9945	0.9918	0.9932	0.0082	0.9998
**8**	0.9952	0.9941	0.9962	0.9941	0.9952	0.0059	0.9998
**9**	0.9994	0.9989	1	0.9989	0.9994	0.0010	1
**10**	0.9978	0.9971	0.9984	0.9971	0.9978	0.0029	0.9993
**11**	0.9978	0.9957	1	0.9957	0.9978	0.0049	1
**12**	0.9998	0.9996	1	0.9996	0.9998	0.0003	1
**13**	0.9998	0.9997	1	0.9997	0.9998	0.0003	1
**14**	0.9999	0.9998	1	0.9998	0.9999	0.0002	1
**15**	0.995	0.9902	1	0.9901	0.9951	0.0099	1

Table S7 84 putative TFBS motif patterns of 11 TFs from 14 independent ChIP-seq experiments in PCBase with the same TFs as training data for Plant-DTI.

ChIP-seq motif name	E-value	Consensus TFBS sequence	PWM
ABF1_GSE80564;1	0.000000e+00	TGMCACGTGTHH	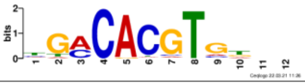
ABF1_GSE80564;2	0.000000e+00	ACGTGKM	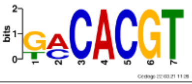
ABF1_GSE80564;3	1.500000e-47	CANGTGK	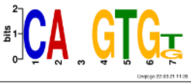
ABF1_GSE80564;4	3.700000e-39	AGARRA	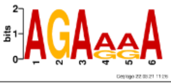
ABF1_GSE80564;5	1.000000e-25	AGARRAAGAVARAGA	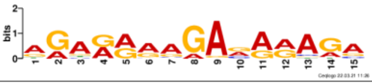
ABF1_GSE80564;6	1.800000e-23	AWYCAACGGYYDAGA	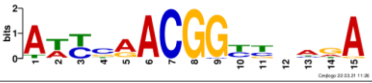
ABF3_GSE80564;1	0.000000e+00	ACGTGKM	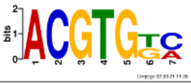
ABF3_GSE80564;2	0.000000e+00	TGMCACGTG	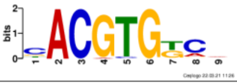
ABF3_GSE80564;3	3.500000e-69	GGYCCM	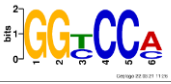
ABF3_GSE80564;4	9.900000e-65	ARAGAVA	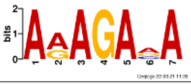
ABF3_GSE80564;5	2.900000e-34	TTTBTYTTYTTYTTT	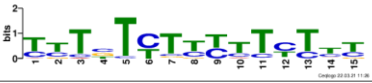
ABF3_GSE80564;6	8.400000e-09	RGRCCCA	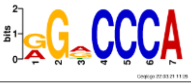
FHY3_GSE69425;1	2.600000e-91	TGNNNTHCACGCGC	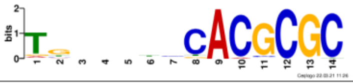
FHY3_GSE69425;2	7.900000e-40	GAWGACACAAAGYCA	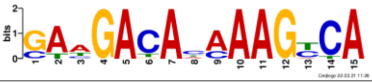
FHY3_GSE69425;3	9.500000e-23	GAAGCAAGAARAAGG	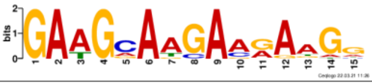
FHY3_GSE69425;4	4.100000e-16	CACKCGC	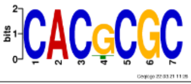
FHY3_GSE69425;5	1.300000e-02	AMTCATA	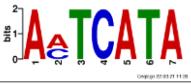
FHY3_GSE69425;6	2.000000e-02	AGCCMA	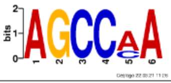

Table S7 (cont.) 84 putative TFBS motif patterns of 11 TFs from 14 independent ChIP-seq experiments in PCBase with the same TFs as training data for Plant-DTI.

ChIP-seq motif name	E-value	Consensus TFBS sequence	PWM
GBF2_GSE80564;1	0.000000e+00	ACGTGKM	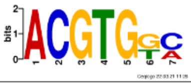
GBF2_GSE80564;2	0.000000e+00	DNHSACGTGKCA	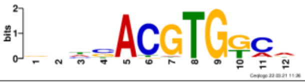
GBF2_GSE80564;3	2.500000e-52	AAKAARA	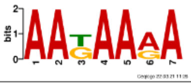
GBF2_GSE80564;4	1.300000e-47	AAAAAAAAAAARAAA	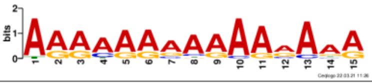
GBF2_GSE80564;5	1.100000e-41	GCTGDC	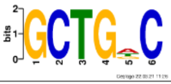
GBF2_GSE80564;6	3.200000e-34	RGAGAGAGAGAGAGR	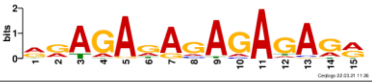
GBF3_GSE80564;1	0.000000e+00	ACGTGKM	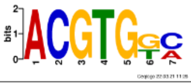
GBF3_GSE80564;2	0.000000e+00	DNHSACGTGKCA	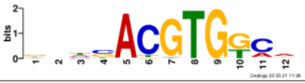
GBF3_GSE80564;3	3.100000e-56	ARRAGA	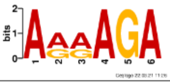
GBF3_GSE80564;4	6.100000e-49	MCAHGTCA	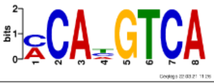
GBF3_GSE80564;5	4.000000e-36	AAAAAAAAAAAAAAA	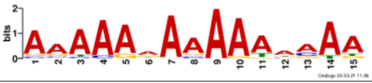
GBF3_GSE80564;6	2.500000e-23	CTCTCTCTCTCTCTY	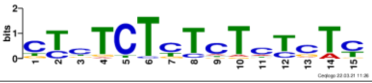
HB5_GSE80564;1	0.000000e+00	ATNATTG	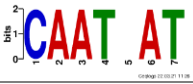
HB5_GSE80564;2	1.700000e-165	AATDATTGR	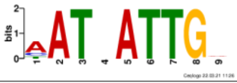
HB5_GSE80564;3	1.900000e-84	GGHCCM	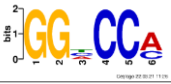
HB5_GSE80564;4	2.500000e-77	TYTYTYTYTYTYTYT	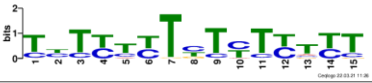
HB5_GSE80564;5	5.800000e-69	CACRYG	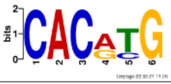

Table S7 (cont.) 84 putative TFBS motif patterns of 11 TFs from 14 independent ChIP-seq experiments in PCBase with the same TFs as training data for Plant-DTI.

ChIP-seq motif name	E-value	Consensus TFBS sequence	PWM
HB5_GSE80564;6	1.800000e-36	GGCCCAY	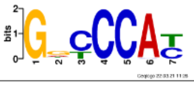
HB6_GSE80564;1	2.500000e-125	BACGTGK	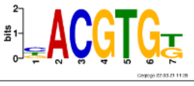
HB6_GSE80564;2	1.700000e-82	RGYCAA	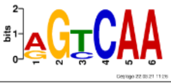
HB6_GSE80564;3	5.800000e-56	GMCACGTGD	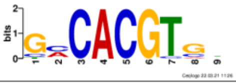
HB6_GSE80564;4	6.200000e-52	ARAGAVA	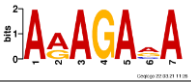
HB6_GSE80564;5	3.200000e-47	YTCTYTYTCTYYYTY	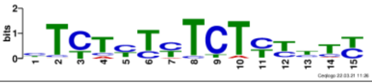
HB6_GSE80564;6	1.700000e-15	TTTTGTYTTYTT	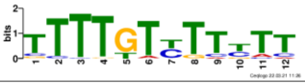
HB7_GSE80564;1	7.500000e-214	ATKATTR	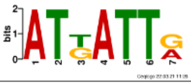
HB7_GSE80564;2	5.900000e-96	BACGTGK	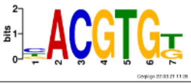
HB7_GSE80564;3	1.400000e-85	BCAATCA	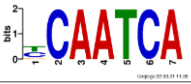
HB7_GSE80564;4	8.200000e-65	YCAATCA	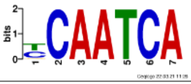
HB7_GSE80564;5	1.300000e-37	MCACGTGKC	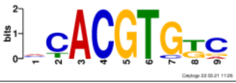
HB7_GSE80564;6	2.400000e-32	RARARARAGARARAG	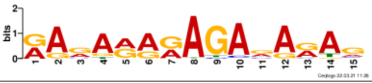
PIF4_GSE35315;1	3.900000e-300	CACRTGSMN	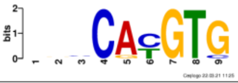
PIF4_GSE35315;2	1.900000e-254	BCACRTG	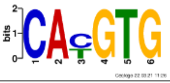
PIF4_GSE35315;3	8.900000e-184	CCCATNHHH	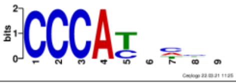
PIF4_GSE35315;4	3.400000e-153	CCCAY	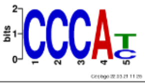

Table S7 (cont.) 84 putative TFBS motif patterns of 11 TFs from 14 independent ChIP-seq experiments in PCBase with the same TFs as training data for Plant-DTI.

ChIP-seq motif name	E-value	Consensus TFBS sequence	PWM
PIF4_GSE35315;5	3.000000e-58	TCTCTCTCTYYYTCT	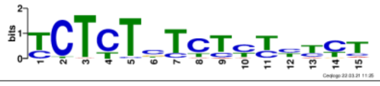
PIF4_GSE35315;6	3.700000e-57	ARAGAVA	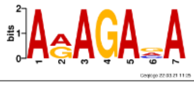
PIF4_GSE68193;1	0.000000e+00	DNSCAYGTGANN	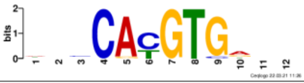
PIF4_GSE68193;2	4.800000e-311	CACRTG	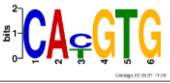
PIF4_GSE68193;3	2.300000e-79	RGAGAGAGARARRGR	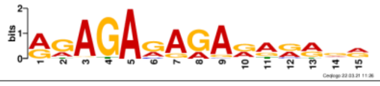
PIF4_GSE68193;4	1.600000e-39	DRTGGGHCC	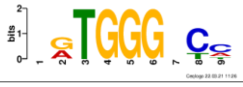
PIF4_GSE68193;5	4.400000e-29	AGAGAG	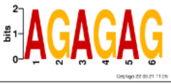
PIF4_GSE68193;6	1.300000e-23	GRCCCAY	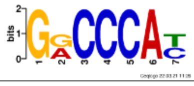
PIF5_GSE68193;1	0.000000e+00	YCACGTGGMHHHNNY	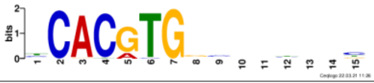
PIF5_GSE68193;2	1.900000e-82	BCACRTG	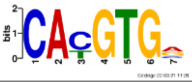
PIF5_GSE68193;3	7.000000e-43	RAGARARARAGA	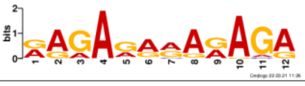
PIF5_GSE68193;4	4.300000e-09	ACGTGKM	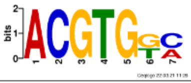
PIF5_GSE68193;5	2.500000e-08	ACGYG	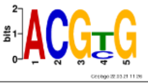
PIF5_GSE68193;6	3.400000e-05	AGAGARR	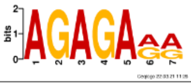
SEP3-2days_GSE46987;1	6.300000e-280	CCAAAAAWGGAAARW	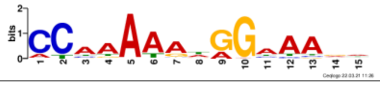
SEP3-2days_GSE46987;2	3.000000e-75	AGARARAGARRRARR	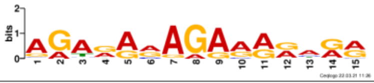
SEP3-2days_GSE46987;3	7.900000e-14	AAAKRG	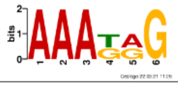

Table S7 (cont.) 84 putative TFBS motif patterns of 11 TFs from 14 independent ChIP-seq experiments in PCBase with the same TFs as training data for Plant-DTI.

ChIP-seq motif name	E-value	Consensus TFBS sequence	PWM
SEP3-2days_GSE46987;4	3.500000e-05	CCAWA	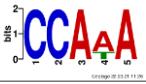
SEP3-2days_GSE46987;5	1.800000e-03	CGTTGACTTTTT	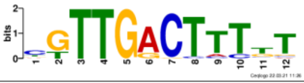
SEP3-2days_GSE46987;6	1.900000e-03	AGARAG	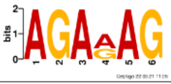
SEP3-4days_GSE46987;1	8.300000e-200	CCAAAAAWRGWAAWW	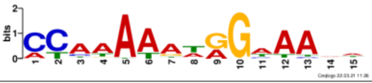
SEP3-4days_GSE46987;2	7.000000e-146	RARAGARARARARAR	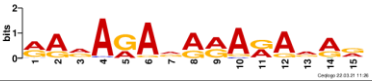
SEP3-4days_GSE46987;3	6.700000e-106	MCMAAA	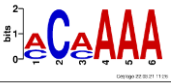
SEP3-4days_GSE46987;4	1.200000e-79	CACRTG	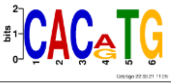
SEP3-4days_GSE46987;5	3.800000e-78	ADAKAGA	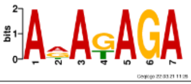
SEP3-4days_GSE46987;6	5.500000e-35	GMCACGTGD	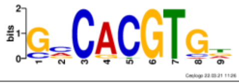
SEP3-8days_GSE46987;1	7.800000e-166	YYTYTYTYTYTYTYT	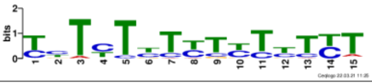
SEP3-8days_GSE46987;2	1.300000e-120	MCMAAA	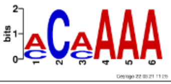
SEP3-8days_GSE46987;3	2.100000e-95	CACRTG	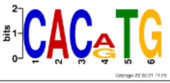
SEP3-8days_GSE46987;4	2.000000e-87	GGHCCAY	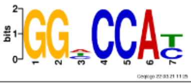
SEP3-8days_GSE46987;5	1.400000e-85	CCAAAAAWRGAAADW	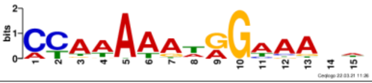
SEP3-8days_GSE46987;6	6.000000e-53	KKGGWCC	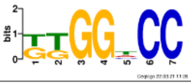

Table S8 37 putative TFBS motif patterns of 8 TFs from independent ChIP-seq experiments in PCBase which are different from TFs in the training data for Plant-DTI but contain the same DBDs.

ChIP-seq motif name	E-value	Consensus TFBS sequence	PWM
ANAC102_GSE80564;1	9.600000e-220	RARRRAGARRAARAR	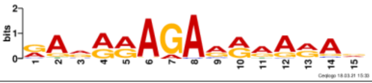
ANAC102_GSE80564;2	2.400000e-98	GWSACGTGTVNR	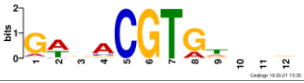
ANAC102_GSE80564;3	1.100000e-64	CACGB	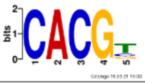
ANAC102_GSE80564;4	1.100000e-30	ARNCAAA	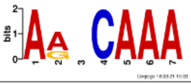
ANAC102_GSE80564;5	7.600000e-27	GWAGAAGA	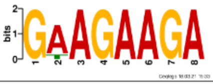
ANAC102_GSE80564;6	5.500000e-18	TTTTGYTTTBKY	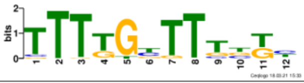
AZF1_GSE84483;1	3.100000e-23	AGGCTAGGACACCAC	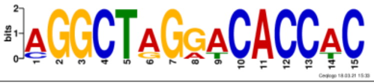
AZF1_GSE84483;2	3.000000e-21	CTTSATTGYGSTTGT	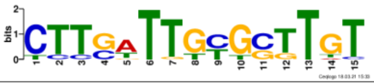
AZF1_GSE84483;3	4.300000e-21	GTTGTTGAACCTGTT	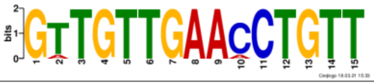
BZIP28_GSE94146;1	5.400000e-22	RRAAAGAAAGAARRV	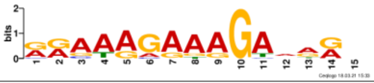
BZIP28_GSE94146;2	2.100000e-05	AWWATGMCACGTGKT	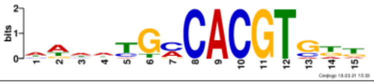
BZIP28_GSE94146;3	5.000000e-03	AAGAARG	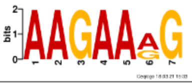
BZIP28_GSE94146;4	9.300000e-03	ACGTGGM	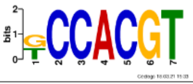
BZIP28_GSE94146;5	2.400000e-02	AACCAWT	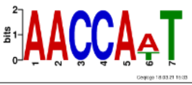
DREB2A_GSE80564;1	0.000000e+00	AWGKCGGTK	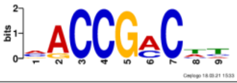
DREB2A_GSE80564;2	5.900000e-80	GKCGGY	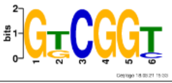
DREB2A_GSE80564;3	3.800000e-17	TYHBTCTTYTTYTYY	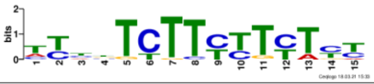

Table S8 (cont.) 37 putative TFBS motif patterns of 8 TFs from independent ChIP-seq experiments in PCBase which are different from TFs in the training data for Plant-DTI but contain the same DBDs.

ChIP-seq motif name	E-value	Consensus TFBS sequence	PWM
DREB2A_GSE80564;4	2.700000e-05	GMCACGTGT	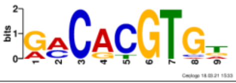
DREB2A_GSE80564;5	5.600000e-03	CACGTG	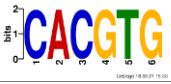
DREB2A_GSE80564;6	2.200000e-02	CTTYTTC	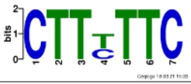
FBH3_GSE80564;1	4.500000e-07	GRBGWGAGAGAGAS	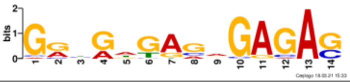
FBH3_GSE80564;2	2.600000e-04	RCRCGTGGV	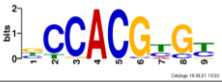
HAT22_GSE80564;1	0.000000e+00	ATBATTR	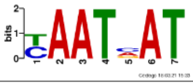
HAT22_GSE80564;2	4.100000e-148	HYAATCATTDD	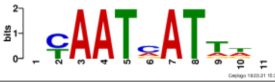
HAT22_GSE80564;3	2.200000e-58	BACGTGK	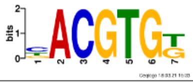
HAT22_GSE80564;4	3.200000e-56	ARAGAVA	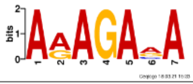
HAT22_GSE80564;5	2.100000e-22	ARRRAGARAGAGARR	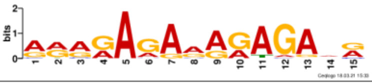
HAT22_GSE80564;6	4.500000e-15	BKMCACGTG	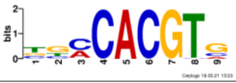
RGA_GSE94926;1	1.300000e-33	TYYTTTGTYTYTTTY	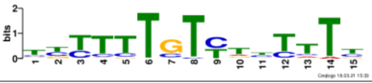
RGA_GSE94926;2	9.600000e-15	RTGGGRCCCAYD	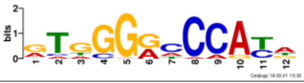
RGA_GSE94926;3	3.000000e-06	RSHVRCRCGTGDVR	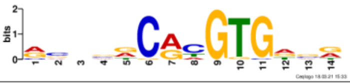
RGA_GSE94926;4	4.400000e-04	GACAM	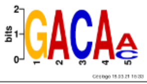
RGA_GSE94926;5	1.100000e-03	GGTCCM	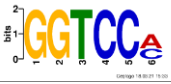

Table S8 (cont.) 37 putative TFBS motif patterns of 8 TFs from independent ChIP-seq experiments in PCBase which are different from TFs in the training data for Plant-DTI but contain the same DBDs.

ChIP-seq motif name	E-value	Consensus TFBS sequence	PWM
SOC1_GSE45846;1	2.900000e-201	CCAAAAAWGGAAAD	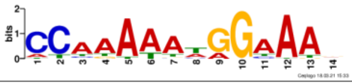
SOC1_GSE45846;2	2.500000e-07	TGGWAA	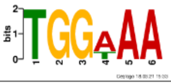
SOC1_GSE45846;3	6.400000e-04	TYYTYTBYTTTTTYT	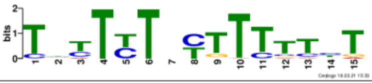
SOC1_GSE45846;4	1.100000e-03	CCATTTTTGGWA	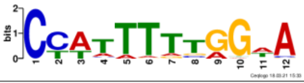

Table S9 The information of positive and negative data corresponded to TFs ChIP-seq data. The number of intergenic regions in *Arabidopsis thaliana* genome subtracted by *Arabidopsis thaliana* DNA hypersensitivity sites (DHSs) (A), number of the regions that TF can interact to DNA regions, ChIP-seq, DAP-seq data were removed (B and C), negative regions were collected only regions with > 201 bp and it do not contain ambiguous nucleotides (D and E) and, all TFBS sequences that used in training data of particular TF found in negative regions were used to subtract and the remaining regions were sampled in the same proportion as positive binding regions data and used as negative data for prediction in each model (F to H).

TF name	Gene ID	TF family	DBD type	Model length of Plant-DTI	No. of positive binding region	No. of negative region (A)	No. of negative region after subtracted by ChIP-seq of same TFs (B)	No. of negative region after subtracted by DAP-seq of same TFs (C)	No. of negative region with length > 201 bp (D)	No. of negative region sequences with no ambiguous nucleotides (E)	Negative sequences as input for PWM (F)	Negative sequences as input for TSPTFBS (G)	Negative sequences as input for Plant-DTI (H)
ABF1	AT1G49720	bZIP	bZIP_1	11<br>10	2,925	40,673	39,732	39,732	28,900	28,835	2,925	5,850	1,104,806<br>1,105,667
ABF3	AT4G34000	bZIP	bZIP_1	8	5,954	40,673	38,633	38,633	28,093	28,029	5,954	11,908	2,219,126
FHY3	AT3G22170	FAR1	FAR1	12	119	40,673	40,687	40,687	29,667	29,601	119	238	45,008
GBF3	AT2G46270	bZIP	bZIP_1	10	5,949	40,673	40,261	40,257	29,157	29,092	5,949	11,898	2,248,464
HB5	AT5G65310	HD-ZIP	Homeobox	13<br>9	4,037	40,673	39,371	40,095	27,775	27,710	4,037	8,074	1,516,548<br>1,524,368
HB6	AT2G22430	HD-ZIP	Homeobox	9	5,934	40,673	39,239	39,387	28,118	28,053	5,934	11,868	2,239,028
HB7	AT2G46680	HD-ZIP	Homeobox	10	5,222	40,673	38,920	39,078	28,021	27,956	5,222	10,444	1,973,941
PIF4	AT2G43010	bHLH	HLH	8	3,847	40,673	39,422	39,422	28,744	28,674	3,847	7,694	1,435,751
PIF5	AT3G59060	bHLH	HLH	8	661	40,673	40,431	40,431	29,498	29,432	661	1,322	245,561
SEP3	AT1G24260	MIKC	SRF-TF	11	8,430	40,673	39,956	39,956	28,951	28,885	8,430	16,860	3,184,612

Table S10 The information of positive and negative data corresponded to TFs DAP-seq data. The number of intergenic regions in *Arabidopsis thaliana* genome subtracted by *Arabidopsis thaliana* DNA hypersensitivity sites (DHSs) (A), number of the regions that TF can interact to DNA regions of DAP-seq data were removed (B), negative regions were collected only regions with > 201 bp and it do not contain ambiguous nucleotides (C and D) and, all TFBS sequences that used in training data of particular TF found in negative regions were used to subtract and the remaining regions were sampled in the same proportion as positive binding regions data and used as negative data for prediction in each model (E to G).

TF name	Gene ID	TF family	DBD type	Model length of Plant-DTI	No. of positive binding region	No. of negative region (A)	No. of negative region after subtracted by DAP-seq of same TFs (B)	No. of negative region with length > 201 bp (C)	No. of negative region sequences with no ambiguous nucleotides (D)	Negative sequences as input for PWM (E)	Negative sequences as input for TSPTFBS (F)	Negative sequences as input for Plant-DTI (G)
ABF2	AT1G45249	bZIP	bZIP_1	13	1,902	40,673	40,664	29,642	29,576	1,902	3,804	714,001
ANAC083	AT5G13180	NAC	NAM	10	3,254	40,673	40,776	29,505	29,439	3,254	6,508	1,231,130
ANAC092	AT5G39610	NAC	NAM	9	2,333	40,673	40,717	29,551	29,485	2,333	4,666	879,659
ANL2	AT4G00730	HD-ZIP	Homeobox	11	3,515	40,673	41,098	29,269	29,203	3,515	7,030	1,329,213
AT1G19000	AT1G19000	MYB_related	Myb_DNA-binding	10	3,989	40,673	40,918	29,418	29,352	3,989	7,978	1,507,011
AT1G25550	AT1G25550	MYB_related	Myb_DNA-binding	10	1,211	40,673	40,703	29,611	29,545	1,211	2,422	458,289
AT1G64620	AT1G64620	Dof	zf-Dof	10	5,447	40,673	40,939	29,275	29,209	5,447	10,894	2,041,369
AT1G74840	AT1G74840	MYB_related	Myb_DNA-binding	11	3,309	40,673	40,932	29,445	29,379	3,309	6,618	1,251,093
AT2G01060	AT2G01060	MYB_related	Myb_DNA-binding	10	2,817	40,673	40,819	29,529	29,463	2,817	5,634	1,065,191
AT2G33710	AT2G33710	ERF	AP2	10	6,059	40,673	40,719	29,609	29,543	6,059	12,118	2,293,476
AT3G04030	AT3G04030	MYB_related	Myb_DNA-binding	9	6,120	40,673	40,877	29,349	29,283	6,120	12,240	2,290,537
AT3G24120	AT3G24120	MYB_related	Myb_DNA-binding	10	10,185	40,673	41,075	29,183	29,117	10,185	20,370	3,830,938
AT4G32800	AT4G32800	ERF	AP2	8	1,007	40,673	40,694	29,628	29,562	1,007	2,014	375,983
AT5G29000	AT5G29000	MYB_related	Myb_DNA-binding	10	1,812	40,673	40,752	29,573	29,507	1,812	3,624	682,954
AT5G62940	AT5G62940	Dof	zf-Dof	8	12,931	40,673	41,300	28,691	28,625	12,931	25,862	4,783,488

TF name	Gene ID	TF family	DBD type	Model length of Plant-DTI	No. of positive binding region	No. of negative region (A)	No. of negative region after subtracted by DAP-seq of same TFs (B)	No. of negative region with length > 201 bp (C)	No. of negative region sequences with no ambiguous nucleotides (D)	Negative sequences as input for PWM (E)	Negative sequences as input for TSPTFBS (F)	Negative sequences as input for Plant-DTI (G)
bHLH31	AT1G59640	bHLH	HLH	9	1,342	40,673	40,676	29,645	29,579	1,342	2,684	507,197
BIM2	AT1G69010	bHLH	HLH	10	7,691	40,673	40,699	29,544	29,478	7,691	15,382	2,909,293
bZIP68	AT1G32150	bZIP	bZIP_1	9	5,497	40,673	40,665	29,618	29,552	5,497	10,994	2,071,899
CBF2	AT4G25470	ERF	AP2	8	1,555	40,673	40,707	29,624	29,558	1,555	3,110	579,375
CBF3	AT4G25480	ERF	AP2	10	2,764	40,673	40,710	29,601	29,535	2,764	5,528	1,045,353
cdf3	AT3G47500	Dof	zf-Dof	9	3,570	40,673	40,791	29,435	29,369	3,570	7,140	1,343,391
COG1	AT1G29160	Dof	zf-Dof	8	4,873	40,673	40,851	29,356	29,290	4,873	9,746	1,820,701
CRF4	AT4G27950	ERF	AP2	8	1,503	40,673	40,678	29,647	29,581	1,503	3,006	561,632
EPR1	AT1G18330	MYB_related	Myb_DNA-binding	9	8,863	40,673	41,607	28,776	28,710	8,863	17,726	3,343,615
ERF1	AT3G23240	ERF	AP2	10	1,346	40,673	40,685	29,642	29,576	1,346	2,692	509,442
ERF1	AT3G23240	ERF	AP2	8	1,346	40,673	40,685	29,642	29,576	1,346	2,692	501,781
ERF105	AT5G51190	ERF	AP2	8	5,552	40,673	40,699	29,618	29,552	5,552	11,104	2,070,676
ERF11	AT1G28370	ERF	AP2	10	1,541	40,673	40,690	29,642	29,576	1,541	3,082	581,849
ERF13	AT2G44840	ERF	AP2	8	3,942	40,673	40,691	29,619	29,553	3,942	7,884	1,468,603
ERF15	AT2G31230	ERF	AP2	8	6,378	40,673	40,731	29,586	29,520	6,378	12,756	2,379,991
ERF3	AT1G50640	ERF	AP2	8	2,064	40,673	40,673	29,647	29,581	2,064	4,128	769,758
ERF4	AT3G15210	ERF	AP2	8	3,421	40,673	40,671	29,647	29,581	3,421	6,842	1,272,884
ERF7	AT3G20310	ERF	AP2	10	6,398	40,673	40,691	29,618	29,552	6,398	12,796	2,421,770
ERF8	AT1G53170	ERF	AP2	10	4,004	40,673	40,669	29,648	29,582	4,004	8,008	1,514,176
GATA11	AT1G08010	GATA	GATA	8	1,491	40,673	40,685	29,623	29,557	1,491	2,982	555,459
GATA15	AT3G06740	GATA	GATA	9	1,043	40,673	40,723	29,613	29,547	1,043	2,086	392,461
NAC2	AT5G04410	NAC	NAM	12	3,361	40,673	40,746	29,516	29,450	3,361	6,722	1,267,270
NST1	AT2G46770	NAC	NAM	10	3,891	40,673	40,831	29,436	29,370	3,891	7,782	1,471,176
OBP4	AT5G60850	Dof	zf-Dof	10	5,063	40,673	40,927	29,316	29,250	5,063	10,126	1,916,030
OBP4	AT5G60850	Dof	zf-Dof	11	5,063	40,673	40,927	29,316	29,250	5,063	10,126	1,914,687
RAP2.6	AT1G43160	ERF	AP2	8	1,564	40,673	40,675	29,650	29,584	1,564	3,128	581,080
SPL14	AT1G20980	SBP	SBP	9	1,211	40,673	40,712	29,606	29,540	1,211	2,422	451,437

TF name	Gene ID	TF family	DBD type	Model length of Plant-DTI	No. of positive binding region	No. of negative region (A)	No. of negative region after subtracted by DAP-seq of same TFs (B)	No. of negative region with length > 201 bp (C)	No. of negative region sequences with no ambiguous nucleotides (D)	Negative sequences as input for PWM (E)	Negative sequences as input for TSPTFBS (F)	Negative sequences as input for Plant-DTI (G)
SPL5	AT3G15270	SBP	SBP	9	2,127	40,673	40,721	29,595	29,529	2,127	4,254	802,459
TBP3	AT5G67580	MYB_related	Myb_DNA-binding	8	2,618	40,673	40,727	29,573	29,507	2,618	5,236	977,443
TGA1	AT5G65210	bZIP	bZIP_1	10	3,574	40,673	40,756	29,558	29,492	3,574	7,148	1,351,166
TGA1	AT5G65210	bZIP	bZIP_1	11	3,574	40,673	40,756	29,558	29,492	3,574	7,148	1,350,407
TGA2	AT5G06950	bZIP	bZIP_1	10	5,456	40,673	40,702	29,624	29,558	5,456	10,912	2,062,378
TGA4	AT5G10030	bZIP	bZIP_1	10	1,868	40,673	40,690	29,634	29,568	1,868	3,736	705,891
TGA6	AT3G12250	bZIP	bZIP_1	10	5,679	40,673	40,782	29,519	29,453	5,679	11,358	2,150,335
WRKY14	AT1G30650	WRKY	WRKY	10	2,399	40,673	40,711	29,576	29,510	2,399	4,798	907,387
WRKY15	AT2G23320	WRKY	WRKY	10	2,944	40,673	40,742	29,545	29,479	2,944	5,888	1,113,241
WRKY18	AT4G31800	WRKY	WRKY	10	6,642	40,673	40,772	29,458	29,392	6,642	13,284	2,509,413
*WRKY22	AT4G01250	WRKY	WRKY	10	6,105*	40,673	40,881	29,361	29,295	406*	812	55,960
WRKY27	AT5G52830	WRKY	WRKY	9	1,432	40,673	40,694	29,613	29,547	1,432	2,864	532,491
WRKY30	AT5G24110	WRKY	WRKY	10	1,994	40,673	40,724	29,594	29,528	1,994	3,988	754,114
WRKY75	AT5G13080	WRKY	WRKY	8	8,136	40,673	40,793	29,376	29,311	8,136	16,272	3,032,318

* The number of negative sequences of WRKY22 is not same proportions to number of positive binding region because the negative region sequences with no ambiguous nucleotides contain positive training data in their regions. Only 406 negative region sequences with no ambiguous nucleotides and positive training data in their regions were used as negative sequences.
